# Identification and Expression Analysis of Chemosensory Genes in the Antennal Transcriptome of Chrysanthemum Aphid *Macrosiphoniella sanborni*

**DOI:** 10.3390/insects13070597

**Published:** 2022-06-29

**Authors:** Jian Zhong, Yuxin Wang, Yufan Lu, Xiaoou Ma, Qian Zhang, Xiaoyue Wang, Qixiang Zhang, Ming Sun

**Affiliations:** 1 Beijing Key Laboratory of Ornamental Plants Germplasm Innovation and Molecular Breeding, National Engineering Research Center for Floriculture, Beijing Laboratory of Urban and Rural Ecological Environment, Key Laboratory of Genetics and Breeding in Forest Trees and Ornamental Plants of Ministry of Education, School of Landscape Architecture, Beijing Forestry University, Beijing 100083, China; zhongjianbjfu@163.com (J.Z.); wangyuxinbjfu@163.com (Y.W.); 18121069325@163.com (Y.L.); xiaoou0629@163.com (X.M.); zhangqian3494@163.com (Q.Z.); YY838379811@163.com (X.W.); zqxbjfu@126.com (Q.Z.); 2 Beijing Advanced Innovation Center for Tree Breeding by Molecular Design, Beijing Forestry University, Beijing 100083, China

**Keywords:** *Macrosiphoniella sanborni*, antennal transcriptome, chemosensory genes, aphid

## Abstract

**Simple Summary:**

The olfactory system is key for insects to receive external chemical signals, and various chemosensory genes are involved in this process. Thus, research focused on the olfactory mechanisms of insects can provide theoretical guidance for the development of effective green pest-control measures. In this study, an antennal transcriptome analysis of the chrysanthemum aphid (*Macrosiphoniella sanborni*) was conducted to identify putative chemosensory genes. The relative relationships of chemosensory genes between chrysanthemum aphids and other aphid species were analyzed. Then, the wing-specific and odor-specific expression profiles of these candidate genes were examined. This study contributes to our understanding of the olfactory system and lays the foundation for functional studies of the chemoreception mechanism in *M. sanborni*.

**Abstract:**

As one of the most destructive oligophagous pests, the chrysanthemum aphid (*Macrosiphoniella sanborni*) has seriously restricted the sustainable development of the chrysanthemum industry. Olfaction plays a critical role in the environmental perception of aphids, but very little is currently known about the chemosensory mechanism of *M. sanborni*. In this study, four MsanOBPs, four MsanCSPs, eight MsanORs, two MsanIRs and one MsanSNMP were identified among the 28,323 unigenes derived from the antennal transcriptome bioinformatic analysis of *M. sanborni* adults. Then, comprehensive phylogenetic analyses of these olfactory-related proteins in different aphid species were performed using multiple sequence alignment. Subsequently, the odor-specific and wing-specific expression profiles of these candidate chemosensory genes were investigated using quantitative real-time PCR. The data showed that most of these chemosensory genes exhibited higher expression levels in alate aphids. Among them, *MsanOBP9*, *MsanOR2*, *MsanOR4*, *MsanOR43b-1*, *MsanCSP1*, *MsanCSP2*, *MsanCSP4*, *MsanIR25a* and *MsanIR40a* in alate aphids showed remarkably higher expression levels than in apterous aphids under the effect of the host plant volatiles, indicating that these genes may take part in the specific behaviors of alate adults, such as host recognition, oviposition site selection and so on. This study lays the groundwork for future research into the molecular mechanism of olfactory recognition in *M. sanborni*.

## 1. Introduction

The chrysanthemum aphid *Macrosiphoniella sanborni* (Hemiptera: Aphididae) is a major destructive oligophagous pest for chrysanthemums. Chrysanthemum aphids not only rob nutrients and damage the host plants directly but also act as severe chrysanthemum virus vectors (e.g., vein mottle and virus B) [1]. Much time, money and energy have been invested in controlling this pest, and the utilization of pesticides has been the most critical and practical pathway until now. However, pesticide residues, environmental pollution and pesticide resistance resulting from the tremendous application of pesticides have become a severe restriction on the development of the chrysanthemum industry [2]. Thus, it is urgent to establish an efficient and environment-friendly control system for chrysanthemum aphids.

Olfaction-based behaviorally manipulated technology is a green control technology that explicitly regulates target pests’ behavior and has great application potential [3]. Antennae are the most crucial olfactory organs in insects for recognizing and sensing the environment’s chemical cues. Chemical cues emitted by congeneric species, natural enemies and host plants can be received by the antennal sensilla and result in various behavioral choices, including mate confirmation, identification of host location and discovery of natural enemies [4]. A considerable number of studies have shown that odorant receptors (ORs), ionotropic receptors (IRs), gustatory receptors (GRs) odorant binding proteins (OBPs), chemosensory proteins (CSPs) and sensory neuron membrane proteins (SNMPs) play crucial roles in the chemoreception of insects [5]. Compared with model species, such as *Drosophila melanogaster* and *Anopheles gambiae*, there has been comparatively little research progress on aphids recently. Several antennal transcriptome studies have been undertaken to identify the candidate chemosensory genes of several aphids, including *Acyrthosiphon pisum*, *Sitobion avenae*, *Aphis gossypii*, *Myzus persicae*, *Rhopalosiphum padi* and so on [6,7,8,9]. Furthermore, with the development of genome sequencing technology and bioinformatics, a growing number of chemosensory genes have been identified [10,11,12]. Moreover, growing evidence suggests that aphid chemosensory genes play a crucial role in detecting chemical cues, especially for some pheromones. (E)- β-farnesene has been proved to be detected by the pea aphid and green peach aphid with the help of ApisOR5, ApisOBP3, ApisOBP7, ApisOBP9, MperOBP3, MperOBP7 and MperOBP9 [13,14,15]. Furthermore, ApisOBP9 and SaveOBP9 were also found to bond with volatiles, such as tetradecane and 1-hexadecanol, which indicates that OBPs have poor specificity [16,17]. Other studies on MperOR23, RpadCSP4 and RpadCSP5 have provided important information on the function of aphids’ chemosensory genes [18,19]. So far, however, little attention has been paid to characterizing the function of aphids’ IRs, GRs and SNMPs.

The aphid genus *Macrosiphoniella* includes about 159 recognized species, which mainly feed on various Asteraceae, mostly *Artemisia*, *Achillea*, *Aster*, *Chrysanthemum* and *Helichrysum* [1]. No previous study has reported on the antennal transcriptome of this genus, and little is known about its olfactory molecular basis. In our previous study on the ultrastructure of *M. sanborni* antennal sensilla, significant differences in the secondary rhinaria and placoid sensillum between alate and apterous adults were found [20]. Some previous studies have suggested that the emergence of alate aphids occurs mainly for population expansion, helping in the search for new hosts and alleviating population pressure, and the olfactory sense plays a key role in the wing dimorphism of aphids [21,22]. Hence, it could conceivably be hypothesized that the differences in antenna structure lead to different expression profiles of chemosensory genes, which may be the cause of the role division between alate and apterous aphids. Therefore, identifying chemosensory gene families will help us reveal the olfactory recognition mechanism in *M. sanborni* in much more detail.

In this study, alate and apterous *M. sanborni* were chosen for antennal transcriptome sequencing to explore chemosensory genes in the two winged-type aphids. Then, the phylogenetic relationships of these candidate genes to other aphid species were analyzed. Furthermore, the expression profiles of these chemosensory genes were analyzed by combining DEG analysis of transcriptomes with odor- and wing-specific expression using quantitative real-time PCR. This is the first study to undertake a relatively systematic analysis of chemosensory genes of *M. sanborni*. These putative chemosensory genes and their specific expression profiles will greatly help in understanding the olfactory system and in functional studies of the chemoreception mechanism in *M. sanborni*.

## 2. Materials and Methods

### 2.1. Ethics Statement

Chrysanthemum aphids (*M. sanborni*) were from a parthenogenetic colony initially collected from chrysanthemums at the Chrysanthemum Germplasm Resource Preserving Centre, Beijing Forestry University, China, which is not privately owned or protected. The chrysanthemum aphid is neither endangered nor protected, so no specific permission was required for its collection.

### 2.2. Aphid Rearing and Tissue Collection

*M. sanborni* was reared on chrysanthemum plants (*C. morifolium* ”Jinglinqiuge”) at 22 ± 2 °C, 65 ± 5% relative humidity, with a photoperiod of 16:8 (light:dark) in the breeding greenhouse of the National Flower Engineering Center, Beijing, China. In order to obtain enough alate aphids, the feeding plants of the aphids were limited with transparent mesh to create a higher population density. After about eight generations, alate and apterous adults were separated into two different containers. Then, antennae were excised from aphids using microscopic ophthalmic scissors. Each sample had about 500 pairs of antennae with three replicates and was immediately frozen in liquid nitrogen and stored at −80 °C.

### 2.3. cDNA Library Construction and Illumina Sequencing

Total RNA of alate and apterous antennae were extracted from *M. sanborni* using TRIzol reagent (Invitrogen, Waltham, CA, USA) following the manufacturer’s procedure. The total RNA quantity and purity were analyzed using a Bioanalyzer 2100 and RNA 1000 Nano LabChip Kit (Agilent, Santa Clara, CA, USA), with RIN number >7.0. Poly (A) RNA was purified from total RNA (5 μg) using poly-T oligo-attached magnetic beads and two rounds of purification. Following purification, the mRNA was fragmented into small pieces using divalent cations under elevated temperature. Then, the cleaved RNA fragments were reverse-transcribed to create the final cDNA library in accordance with the protocol for the mRNASeq sample preparation kit (Illumina, San Diego, CA, USA). The average insert size for the paired-end libraries was 300 bp (±50 bp). Then, we performed the paired-end sequencing on an IlluminaHiseq4000 at LC Sciences (Houston, TX, USA) following the vendor’s recommended protocol.

### 2.4. De Novo Assembly

Firstly, Cutadapt [23] and perl scripts were used in-house to remove the reads that contained adaptor contamination, low quality bases and undetermined bases. Then, sequence quality was verified using FastQC (http://www.bioinformatics.babraham.ac.uk/projects/fastqc/) (accessed on 23 August 2020), including the Q20, Q30 and GC content for the clean data. All downstream analyses were based on clean data of high quality. De novo assembly of the transcriptome was performed with Trinity 2.4.0 [24]. Trinity groups transcripts into clusters based on shared sequence content. Such a transcript cluster is very loosely referred to as a ”gene”. The longest transcript in the cluster was chosen as the ”gene” sequence (also known as a unigene).

### 2.5. Identification of Chemosensory Genes

All assembled unigenes were aligned against the non-redundant (Nr) protein database (http://www.ncbi.nlm.nih.gov/) (accessed on 23 August 2020) and the Gene Ontology (GO) (http://www.geneontology.org) (accessed on 23 August 2020), SwissProt (http://www.expasy.ch/sprot/) (accessed on 23 August 2020), Kyoto Encyclopedia of Genes and Genomes (KEGG) (http://www.genome.jp/kegg/) (accessed on 23 August 2020) and eggNOG (http://eggnogdb.embl.de/) (accessed on 23 August 2020) databases using DIAMOND [25] with an Evalue threshold <0.00001. Candidate unigenes encoding putative ORs, IRs, OBPs, CSPs, SNMPs and GRs were selected according to these annotation results in the remote sever. Then, candidate chemosensory genes were manually checked using the blastx program against the Nr database. The open reading frames (ORFs) of all putative chemosensory proteins were predicted using the Expert Protein Analysis System (ExPASy) server (http://web.expasy.org/translate/) (accessed on 25 August 2020). The transmembrane domains (TMDs) of ORs, IRs, and GRs were predicted using TMHMM server, version 2.0 (http://www.cbs.dtu.dk/services/TMHMM/) (accessed on 25 August 2020). Putative N-terminal signal peptides of OBPs and CSPs were predicted using the SignalP 4.0 server (http://www.cbs.dtu.dk/services/SignalP/) (accessed on 25 August 2020) with default parameters.

### 2.6. Sequence and Phylogenetic Analysis

Alignments of amino acid sequences and construction of phylogenetic trees were performed with Mega-X. Neighbor-joining was selected as the statistical method, and node support was assessed using a bootstrap method based on 1000 replicates. The detailed method parameters were obtained from a previous study [26]. The OBP dataset contained 172 OBP sequences identified in 20 Hemiptera species (17 aphids and 3 plant bugs; Supplementary File S1). The CSP dataset contained 77 CSP sequences identified in 10 Hemiptera species (seven aphids and three plant bugs; Supplementary File S2). The OR dataset contained 85 OR sequences identified in 11 Hemiptera species (eight aphids and three plant bugs; Supplementary File S3). The IR dataset contained 39 IR sequences identified in 9 Hemiptera species (seven aphids and two plant bugs; Supplementary File S4). The SNMP dataset contained 19 SNMP sequences identified in 10 Hemiptera species (eight aphids and two plant bugs; Supplementary File S5).

### 2.7. Differentially Expressed Unigene Analysis

Salmon [27] was used to investigate expression levels for unigenes by calculating TPM [28]. The differentially expressed unigenes were selected with log2 (fold change) >1 or log2 (fold change) <−1 and with a statistical significance *p* value < 0.05 using R package edgeR [29]. Next, GO and KEGG enrichment analyses were again performed on the differentially expressed unigenes using perl scripts in-house.

### 2.8. Expression Analysis with qRT-PCR

Quantitative real-time PCR (qRT-PCR) was performed to verify the expression of candidate chemosensory genes. Aphids were reared and collected with the same procedures as before. Then, they were transferred into two unique PC vessels (diameter = 19 cm, height = 20 cm). Air cleaned by activated carbon was transferred into another two PC vessels (diameter = 19 cm, height = 20 cm), one of which was left empty (control) while the other contained *C. morifolium* ”Jinglinqiuge” (a whole plant about 15 cm in height). Then, odors from the CK and host plants were transferred into the aphids’ vessels with the help of an air extraction pump, with flow delivered at 0.1 L/min. After 3 h of exposure, 500 pairs of antennae for each sample were excised and stored as in the procedure described in Section 2.2. Total RNA was extracted using TRIzol reagent (Invitrogen, Carlsbad, CA, USA), and the cDNA was synthesized using a PrimeScript RT Reagent Kit with gDNA Eraser Perfect Real Time (TaKaRa Bio Inc., Dalian, China). Gene-specific primers were designed using IDT (https://sg.idtdna.com/PrimerQuest/Home/Index) (accessed on 23 August 2020) and synthesized by Beijing Ruiboxingke Biotechnology Co., Ltd. (Beijing, China) (Table S1). The relative gene expression level was determined using a SYBR Premix Ex Taq Kit (TaKaRa) on a PikoReal Real-time PCR System. Each 20 μL qRT-PCR contained 2 μL of diluted cDNA template and was amplified as follows: 95 °C for 30 s and 40 cycles of 95 °C for 5 s and 55 °C for 30 s, then 72 °C for 30 s. The candidate genes’ relative expression was quantified with the comparative 2−ΔCT method using the *M. sanborni* β-actin gene as the reference [6]. Each qRT-PCR reaction for each sample was performed in three biological replicates to ensure reproducibility. Data analysis was conducted using SPSS 19.0 (SPSS Inc., Chicago, IL, USA). The significant difference analysis of the target genes for the various organs was performed using one-way nested analysis of variance (ANOVA), followed by least significant difference (LSD) tests.

## 3. Results

### 3.1. Antennal Transcriptome Sequencing and Sequence Assembly

**Figure 1:**
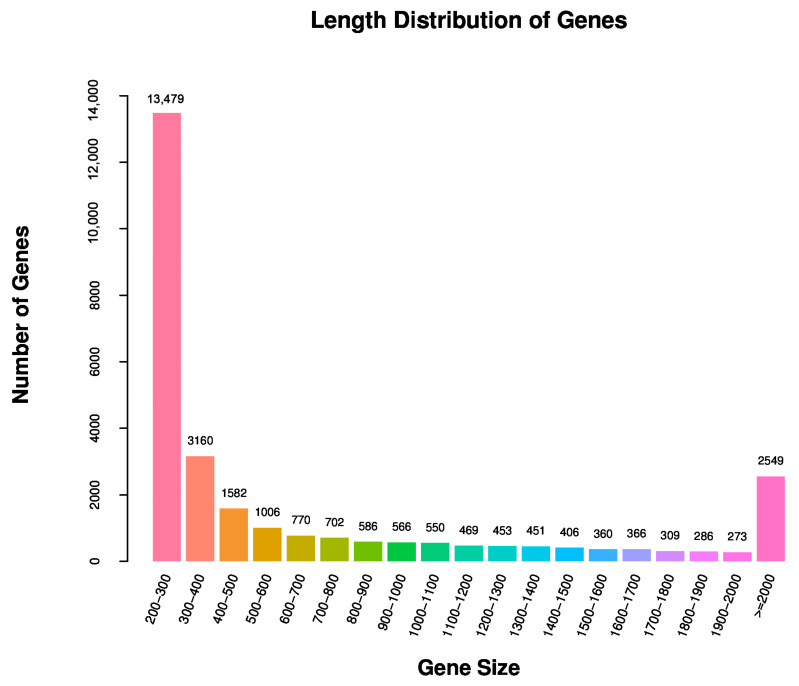
Distribution of unigene size in the *M. sanborni* antennal transcriptome assembly.

*M. sanborni* antennal transcriptomes were sequenced using the Illumina HiSeq 4000 platform combined with Trinity assembly. A total of 49.29 GB of clean data were obtained from six libraries (alate and apterous aphid antennae, each group with three replicates). Approximately 52.74 million and 56.78 million raw reads were obtained from alate and apterous *M. sanborni* antennae, respectively. Finally, about 51.31 and 55.13 million clean reads were obtained after filtering (Table S2). These clean reads were further assembled into 52,249 distinct transcripts and 28,323 unigenes (Table S3). The size distribution analysis showed that the lengths of 6472 unigenes (22.84% of all unigenes) were greater than 1000 bp (Figure 1).

### 3.2. Functional Annotation

**Figure 2:**
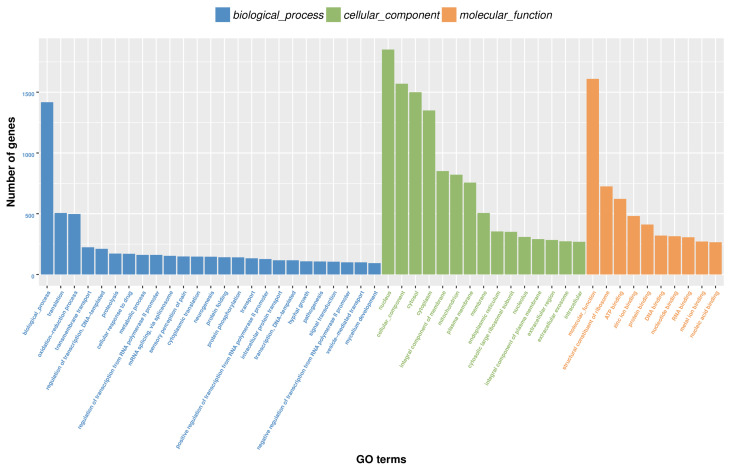
Gene ontology (GO) classification of *M. sanborni* unigenes.

In total, 15,272 unigenes from *M. sanborni* (53.92% of 28,323 unigenes) were annotated in at least one of the databases searched (Nr, Pfam, Swiss-Prot, KEGG, eggNOG and GO databases). Homology searches against the Nr database showed that the *M. sanborni* antennal transcriptome shared the greatest homology with sequences from *Ac. pisum* (51.84%), followed by *Diuraphis noxia* (10.5%) (Figure S1). A total of 11,983 unigenes were annotated, with functional groups classified into 50 subcategories under three main GO categories (“biological process”, “cellular component” and “molecular function”) (Figure 2). Among the GO categories, the *M. sanborni* unigenes were mostly enriched in the “molecular function (GO: 0003674)” and “structural constituent of ribosome (GO: 0003735)” categories at the “molecular function” level, followed by the “nucleus (GO: 0005634)” and “cellular component (GO: 0005575)” categories at the “biological process (GO: 0008150)” level and the “translation (GO: 0006412)” category at the “biological process” level. A total of 6577 unigenes (23.22%) were assigned to the six biological pathways described in the KEGG database: organismal systems, metabolism, genetic information processing, environmental information processing, human diseases and cellular processes (Figure S2). In the environmental information processing group, most genes (788) were involved in signal transduction. Additionally, functional classification was conducted by searching against the Pfam (11,844 unigenes), Swiss-Prot (9836 unigenes) and eggNOG (15,144 unigenes) databases, respectively (Table S4).

### 3.3. Candidate OBPs

**Figure 3:**
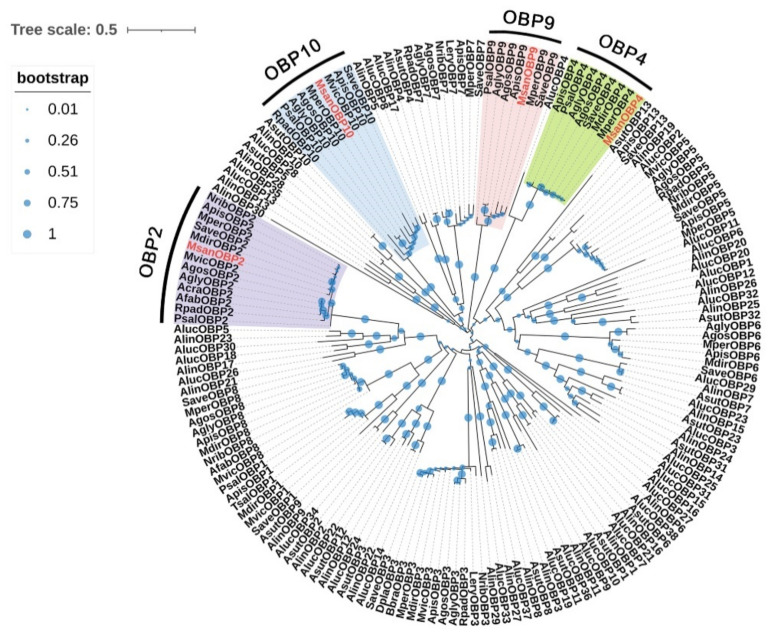
Phylogenetic tree of 172 odorant binding proteins (OBPs) from 20 Hemiptera species. The phylogenetic tree of Hemiptera OBPs was built using 172 OBP sequences from 17 different aphid species (9 from *Myzus persicae*; 9 from *Aphis gossypii*; 11 from *Acyrthosiphon pisum*; 9 from *A. glycines*; 11 from *Sitobion avenae*; 5 from *Pterocomma salicis*; 2 from *A. fabae*; 1 from *A. craccivora*; 1 from *Tuberolachnus salignus*; 6 from *Megoura viciae*; 7 from *Metopolophium dirhodum*; 5 from *Nasonovia ribisnigri*; 5 from *Rhopalosiphum padi*; 2 from *Lipaphis erysimi*; 1 from *Drepanosiphum platanoidis*; 1 from *Brevicoryne brassicae*; 4 from *M. sanborni*) and 3 plant bugs (16 from *Adelphocoris suturalis*; 29 from *Ad. lineolatus*; 38 from *Apolygus lucorum*).

According to the annotation results, eight putative OBPs were identified in the *M. sanborni* antennal transcriptomes. Of these, four OBPs contained full-length ORFs with predicted signal peptide sequences (Table S5). Apart from OBP4, the other three OBPs had the classic hemipteran OBP Cys motif (C1-X_22–32_ -C2-X_3_ -C3-X_36–46_ -C4-X_8–14_ -C5-X_8_ -C6) (Figure S3). OBP4 had 50 amino acids between the first cysteines and second conserved cysteines. Then, 168 OBPs from 16 aphids and 3 plant bugs from the Hymenoptera order were used to construct a phylogenetic tree coupled with four OBPs from *M. sanborni* (Figure 3). Plant bugs (*Adelphocoris suturalis*, *Ad. lineolatus*, *Apolygus lucorum*) were used as outgroups. *M. sanborni* OBPs were clustered into four major groups, each containing several homologous OBPs from different aphid species. Among the aphids’ OBPs, MsanOBPs had significant higher homologies with MperOBPs, SaveOBPs, MdirOBPs and MvicOBPs. However, *A. gossypii*, *A. glycines*, *R. padi* and *Pterocomma salicis* were found to have a comparatively great genetic distance from *M. sanborni* based on the multiple sequence alignment results.

### 3.4. Candidate CSPs

**Figure 4:**
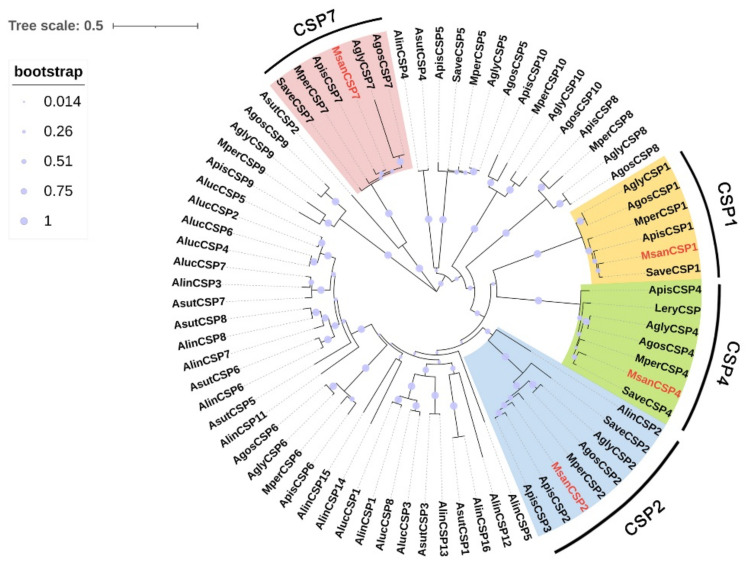
Phylogenetic tree of 77 chemosensory proteins (CSPs) from 10 Hemiptera species. The phylogenetic tree of Hemiptera CSPs was built using 77 CSP sequences from seven different aphid species (9 from *My. persicae*; 9 from *A. gossypii*; 10 from *Ac. pisum*; 9 from *A. glycines*; 5 from *S. avenae*; 1 from *L. erysimi*; 4 from *M. sanborni*) and three plant bugs (8 from *Ad. suturalis*; 14 from *Ad. lineolatus*; 8 from *Ap. lucorum*).

Through bioinformatic analysis, four different unigenes encoding candidate CSPs were identified from antennal transcriptomes of *M. sanborni* (Table S5). Three MsanCSPs (MsanCSP1, MsanCSP4 and MsanCSP7) had complete ORFs, MsanCSP2 only had the 5-prime partial sequence. The length of the deduced proteins ranged from 147 to 213 amino acids, and all of them had predicted signal peptides. All of the identified amino acid sequences possessed the highly conserved four-cysteine profile (C1-X_5–6_ -C2-X_18–19_ -C3-X_2_ -C4) (Figure S4). In order to assign functions to each of the MsanCSPs, a phylogenetic tree was constructed using 76 identified CSPs from nine hemipteran species (Figure 4). The result shown that four MsanCSPs were clustered into four major groups. MsanCSP2 and MsanCSP7 had comparatively closer relationships with ApisCSPs, but MsanCSP1 and MsanCSP4 were closer to SaveCSPs.

### 3.5. Candidate ORs

**Figure 5:**
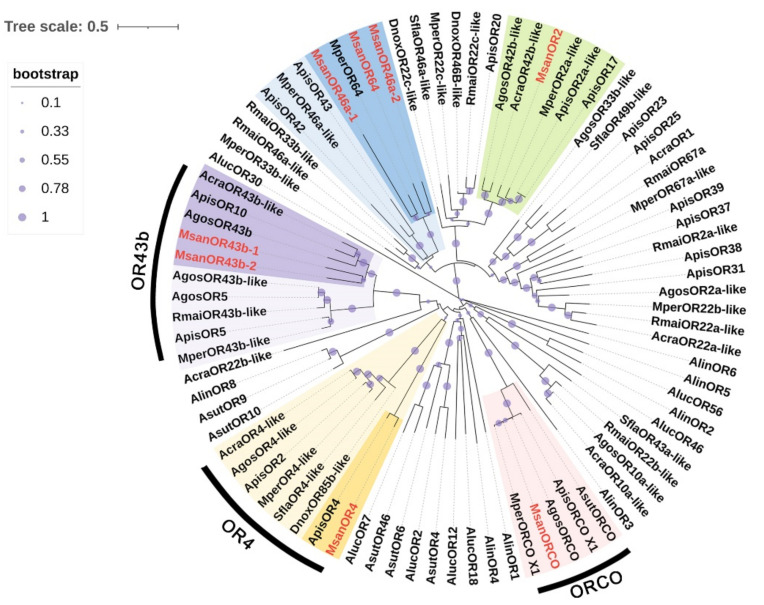
Phylogenetic tree of 85 odorant receptors (ORs) from 11 Hemiptera species. The phylogenetic tree of Hemiptera ORs was built using 85 OR sequences from eight different aphid species (16 from *Ac. pisum*; 10 from *My. persicae*; 8 from *R. maidis*; 7 from *A. craccivora*; 4 from *Sipha. flava*; 9 from *A. gossypii*; 3 from *Diuraphis noxia*; 8 from *M. sanborni*) and three plant bugs (6 from *Ad. suturalis*; 7 from *Ad. lineolatus*; 7 from *Ap. lucorum*).

Eight putative ORs were identified from the antennal transcriptomes of *M. sanborni* (Table S5). Of these, four MsanORs (MsanOR2, MsanOR43b-2, MsanOR46a-1, MsanORCO) contained complete ORFs, indicating that they were nearly full-length, whereas other sequences (MsanOR4, MsanOR43b-1, MsanOR46a-2, MsanOR64) had truncations in the 5′- and/or 3′-terminus. The length of the deduced MsanORs ranged from 323 to 464 amino acids, and transmembrane domains were predicted in all the OR proteins using DeepTMHMM Server; they were predicted to possess 5–7 transmembrane domains (TMDs) (Supplementary File S6). Then, a phylogenetic analysis was performed using our candidate MsanORs and the ORs from 10 other hemipteran species, including *Ac. pisum*, *My. persicae*, *R. maidis* and so on (Figure 5). The results indicated that eight MsanORs were well-segregated from each other with high bootstrap support, and most of them were clustered with at least one hemipteran ortholog. As expected, the olfactory co-receptor, MsanORCO, was clustered into a branch with ORCOs from *My. persicae*, *A. gossypii* and *Ac. pisum* (Figure 5). Among the other MsanORs, three MsanORs (MsanOR46a-1, -46a-2 and -64) were clustered into a mixed group with MperOR64, ApisOR43, MperOR46a-like and ApisOR42. Msan43b-1 and Msan43b-2 were well-clustered with OR43bs from other aphid species, such as AgosOR43b, AcraOR43b-like, AgosOR43b-like, RmaiOR43b-like and MperOR43b-like. The other two MsanORs (MsanOR2 and MsanOR4) were clustered into two appropriate branches, respectively.

### 3.6. Candidate IRs

**Figure 6:**
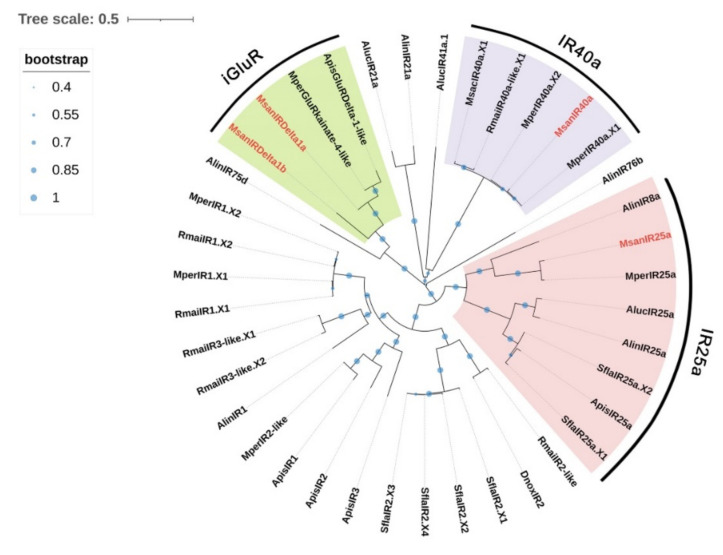
Phylogenetic tree of 39 ionotropic receptors (IRs) from 9 Hemiptera species. The phylogenetic tree of Hemiptera IRs was built using 39 IR sequences from seven different aphid species (6 from *Si. flava*; 5 from *Ac. pisum*; 7 from *My. persicae*; 1 from *D. noxia*; 6 from *R. maidis*; 1 from *Melanaphis sacchari*; 4 from *M. sanborni*) and two plant bugs (6 from *Ad. lineolatus*; 3 from *Ap. lucorum*).

Four unigenes encoding candidate IRs were identified in *M. sanborni* from the alate and apterous antennal transcriptomes (Table S5). Only one of these IRs (MsanIRDelta-1b) had a full-length ORF encoding 527 amino acids. The other three IRs (MsanIRDelta1a, -25a and -40a) had truncations in the 5′- and/or 3′-terminus, and they encoded 368–838 amino acids. These four MsanIRs were found to have three transmembrane domains using DeepTMHMM Server. A phylogenetic analysis was constructed to further distinguish putative IRs (Figure 6). The results showed that two candidate MsanIRs (MsanIR25a and 40a) were clustered with presumed “antennal” orthologues IR25a and IR40a. MsanIRDelta1a and MsanIRDelta1b were found to be members of ionotropic glutamate receptor (iGluR) family, which are the precursors of ionotropic receptors.

### 3.7. Candidate SNMPs

**Figure 7:**
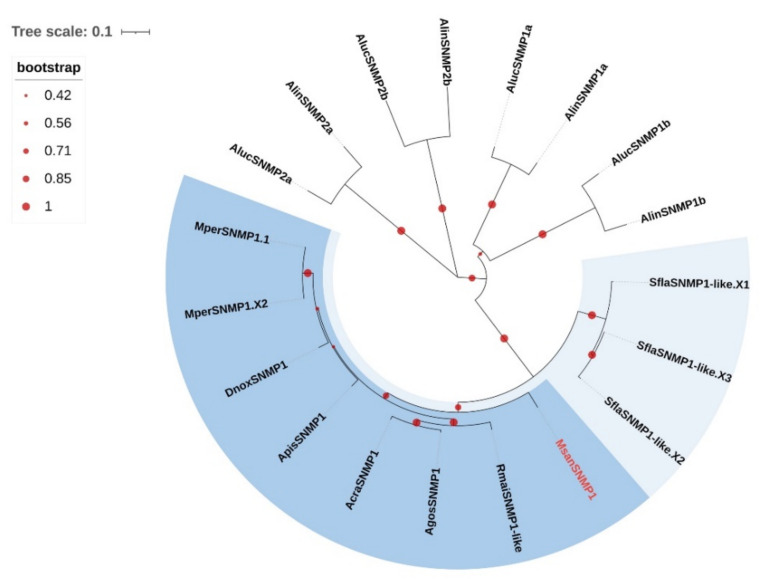
Phylogenetic tree of 19 sensory neuron membrane proteins (SNMPs) from 10 Hemiptera species. The phylogenetic tree of Hemiptera SNMPs was built using 19 SNMP sequences from eight different aphid species (2 from *My. persica*; 3 from *Si. flava*; 1 from *Ac. pisum*; 1 from *D. noxia*; 1 from *A. craccivora*; 1 from *A. gossypii*; 1 from *R. maidis*; 1 from *M. sanborni*) and two plant bugs (4 from *Ad. lineolatus*; 4 from *Ap. lucorum*).

Through bioinformatic analysis, one unigene-encoding candidate SNMP was identified from *M. sanborni* antennal transcriptomes. It was predicted to have a full-length ORF, a single transmembrane domain and five positionally conserved cysteine residues (Table S5, Figure S5). Phylogenetic analysis showed that MsanSNMP1 has a close relationship with its hemipteran orthologs (Figure 7). Moreover, *Sipha flava* can be obviously separated from these aphids.

### 3.8. Differentially Expressed Gene (DEG) Analysis of Candidate Chemosensory Genes

**Figure 8:**
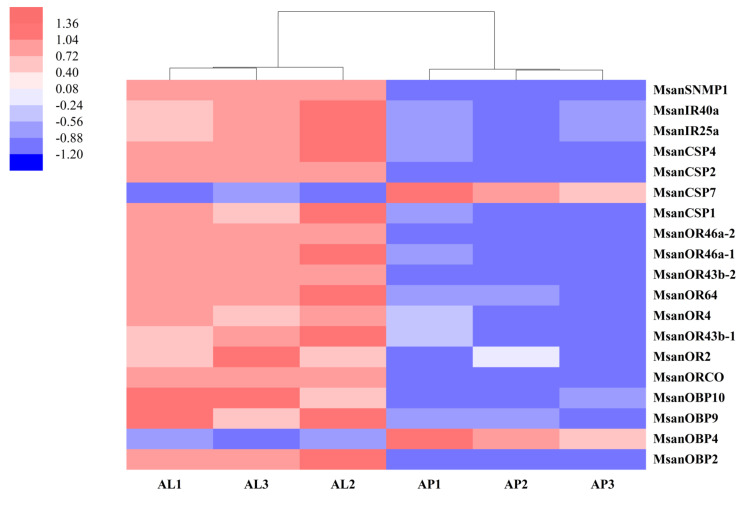
Expression profiles of chemosensory genes in antennal transcriptome of alate (AL) and apterous (AP) *M. sanborni*.

Gene expression levels of alate and apterous antennae-associated chemosensory genes in *M. sanborni* were assessed using TPM values, as represented in a heatmap (Figure 8). The cluster analysis results showed that expression data for both the alate and apterous groups showed good repeatability. Among these chemosensory genes, all *MsanORs*, *MsanIRs* and *MsanSNMPs* had significantly higher expression levels in alate antennae. Similarly, except for *MsanCSP7* and *MsanOBP4*, the other *MsanCSPs* and *MsanOBPs* were highly expressed in alate antennae.

### 3.9. Odor- and Wing-Specific Expression of Candidate Chemosensory Genes

**Figure 9:**
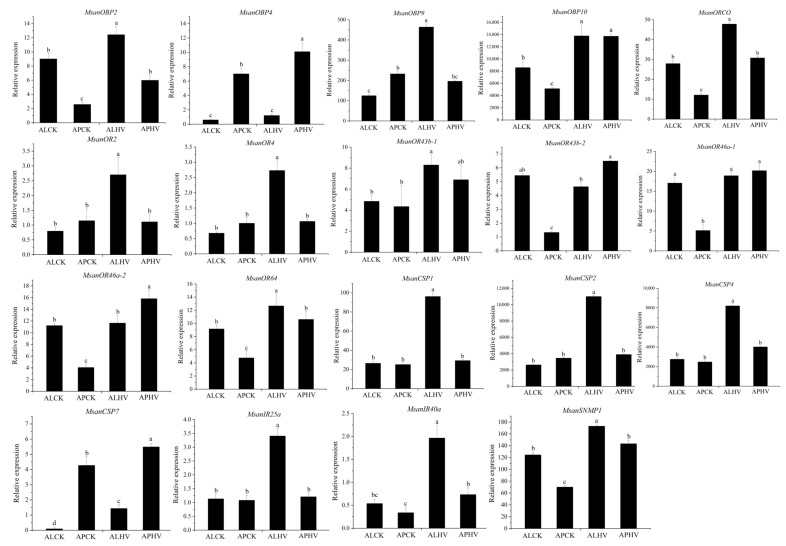
Odor- and wing-specific expression of candidate chemosensory genes of *M. sanborni*. ALCK: alate aphid under control condition, APCK: apterous aphid under control condition, ALHV: alate aphid under the effect of host volatiles, APHV: apterous aphid under the effect of host volatiles. Columns labeled with different letters are significantly different (*p* < 0.05).

The odor- and wing-specific expression profiles of the newly identified genes (four *MsanOBPs*, eight *MsanORs*, four *MsanCSPs*, two *MsanIRs* and one *MsanSNMP*) were investigated using qRT-PCR. The expression patterns of these chemosensory genes were basically consistent with DEG analysis of the transcriptome (Figure 9). Of these chemosensory genes, *MsanOBP2*, *MsanOBP10*, *MsanORCO*, *MsanOR64*, *MsanCSP7* and *MsanSNMP1* were expressed in the antennae of both alate and apterous aphids and significantly induced by the host plant volatiles (HPVs). *MsanOBP4* and *MsanCSP7* shower higher expression levels in the antennae of apterous aphids, and both of them could be induced by the HPVs. In addition, *MsanOBP9*, *MsanOR2*, *MsanOR4*, *MsanOR43b-1*, *MsanCSP1*, *MsanCSP2*, *MsanCSP4*, *MsanIR25a* and *MsanIR40a* in the alate aphids showed remarkably higher expression levels than in apterous aphids under the effect of the HPVs. *Msan43b-2*, *Msan46a-1* and *Msan46a-2* showed a more positive response to the HPVs in the apterous aphids.

## 4. Discussion

As the most representative pest of chrysanthemums, *M. sanborni* has brought substantial economic losses to the chrysanthemum industry. Moreover, the abuse of pesticides has also seriously restricted its sustainable development. Therefore, it is particularly urgent to seek efficient and environment-friendly aphid control strategies for chrysanthemum aphids. A large number of prior studies have demonstrated the vital role of olfactory behavior regulation technology in pest controls [3]. Chemosensory protein plays a crucial role in this process. Chemical cues can be identified and translated into electrical signals by chemosensory proteins, further stimulating the downstream physiological response [5]. The present study was designed to identify the chemosensory proteins of *M. sanborni*, and their phylogenetic relationships with other aphid species. The expression profiles of candidate chemosensory genes were also evaluated to infer their putative functions in aphid chemoreception of semiochemicals.

**Figure 10:**
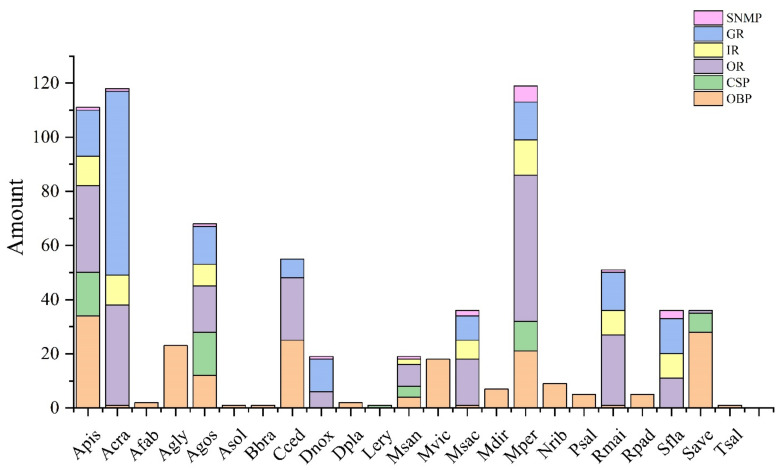
Overview of the identified chemosensory genes of 23 aphid species. Apis: *Ac. pisum*; Acra: *A. craccivora*; Afab: *A. fabae*; Agly: *A. glycines*; Agos: *A. gossypii*; Asol: *Aulacorthum solani*; Bbra: *B. brassicae*; Cced: *Cinara cedri*; Dnox: *D. noxia*; Dpla: *Dr. platanoidis*; Lery: *L. erysimi*; Msan: *M. sanborni*; Mvic: *Meg. viciae*; Msac: *Mel. sacchari*; Mdir: *Met. dirhodum*; Mper: *My. persicae*; Nrib: *N. ribisnigri*; Psal: *P. salicis*; Rmai: *R. maidis*; Rpad: *R. padi*; Sfla: *Si. flava*; Save: *S. avenae*; Tsal: *T. salignus*.

For the first time, we identified four OBPs, eight ORs, four CSPs, two IRs and one SNMP from the antennal transcriptomes of *M. sanborni*. The total number of chemosensory proteins was significantly lower than those of *Ac. pisum*, *A. craccivora*, *A. gossypii* and *My. persicae* (Figure 10). There are many studies on the transcriptomics and functional verification of chemosensory genes of these main aphids [6,15,29,30,31]. More importantly, with the development of whole-genome sequencing technology, chromosome-level genome data have been obtained for these aphids [10,11,32,33]. Therefore, the quantity and quality of the chemosensory genes of these main aphids are much higher than in other aphids. The results of multiple sequence alignment showed that, from the structural point of view, although some proteins had low similarity with other aphid-related proteins due to their poor sequence integrity, the odor sensing proteins of *M. sanborni* could be clustered into the same subfamily branch with the corresponding protein sequences of other aphid species, which indicates that these chemosensory proteins have better conservation among different aphids compared to the outer groups, such as plant bugs.

OBPs, CSPs and ORs are the three prominent families that have been studied most systematically. Previous studies have identified about 82, 38, 45 and 86 proteins (OBPs, CSPs and ORs) in *Ac. pisum*, *A. craccivora*, *A. gossypii* and *My. persicae*, respectively (Table S6), which are significantly higher than for *M. sanborni* in this study. The host diversity of euryphagous aphids makes it necessary to distinguish between more host plant species, which also puts forward higher requirements for olfactory recognition function. Therefore, the number, structure and function of olfactory recognition-related genes increases with the complexity of the overall evolution of aphids. In contrast, for the oligophagous aphids, such as *M. sanborni*, the range of host plants is relatively narrow, mainly focusing on some species or cultivars of the *Chrysanthemum* genus, so the number of chemosensory genes may be lower than for euryphagous aphids. Moreover, compared with the related OBPs, ORS and CSPs identified in other aphids through the whole genome, the transcriptome sequencing samples in this study were the antennae, which probably caused this difference.

In addition, the research on aphid IRs, GRs and SNMPs is minimal. IRs evolved from the ionotropic glutamate receptor (iGluR) family are a kind of conservative ligand-gated ion channel. After the ligand molecules bind with an IR, the channel opens, resulting in the flow of ions inside and outside the membrane and, consequently, membrane potential [34,35]. Early studies have confirmed that insect IRs are the receptors of volatile substances, such as acids and amines, but in recent years, it has been found that IRs can also sense other odor substances and participate in other senses other than smell, including taste, temperature and humidity [36,37]. At present, 68 IRs from about 10 aphids in Aphididae have been identified, but almost all of these IRs were obtained through bioinformatics analysis based on genome sequencing data and thus require further cloning and verification (Figure 10). Moreover, it is unclear how many of these IRs were antenna-specific.

Besides typical odorant receptors, some gustatory receptors expressed in olfactory organs may also be involved in insect olfactory perception, including sugar receptors, bitter receptors and pheromone recognition receptors [38]. Research on aphid gustatory receptors is also mainly affiliated with genome research. A total of 168 GRs have been identified from the genomes of nine aphid species. It is worth noting that the research on the gustatory receptors in aphid antennae has not been published. In this study, although some transcripts were annotated as gustatory receptors, no open reading frames or conservative domains were successfully predicted. It is speculated that the types and expression abundance of GRs in antennae are too low to be detected.

Sensory neuron membrane proteins (SNMPs) were first found in *Antheraea polyphemus*. They are a kind of protein only expressed in the trichoid sensillum of insect antennae, heads (excluding antennae) and thoraxes, and they have the highest expression levels in antennae [39]. Similarly, there is also no relevant research on the function of SNMPs. Only 16 SNMPs have been identified in genomic research from eight aphids, such as *My. persicae*. Similarly to the results of this study, only one SNMP was identified in *Ac. pisum*, *D. noxia*, *A. craccivora* and *R. maidis*. *My. persicae* has the greatest number of SNMPs (six), indicating that SNMPs are the least common among the six major chemosensory gene families in aphids.

A large number of previous studies have shown that insect OBPs have six Cys sites forming three linked disulfide bonds, and their arrangement contributes to the stability of three-dimensional protein structure, which is a conservative feature of insect OBPs. According to the characteristics of conserved sites, OBPs can be divided into classical OBPs, minus-C OBPs, plus-C OBPs, dimer OBPs and atypical OBPs. The amino acid sequence of classical OBPs contains six conserved Cys sites, while minus-C OBPs have only four or five conserved sites [40]. Previous studies have found that most aphids’ OBPs, such as SaveOBPs, AgosOBPs and MperOBPs, are classical OBPs, and a small number of them are plus-C OBPs [6,7,31]. One study found that all of the MsanOBPs have the conserved domain of the classical OBPs. Nevertheless, the motif pattern of MsanOBP4 is not like the hemipteran OBPs’ Cys motif [41]. It has 49 amino acids between the first and second conserved cysteines and 21 amino acids between the fourth and fifth conserved cysteines. However, OBP4 is highly conserved in aphids, which means OBP4s is a unique subfamily with its featured motif pattern.

This study found that MsanCSPs have the typical feature of insect CSPs, with four conserved cysteines fitting the hemipteran Cys spacing motif C1-X_5–6_ -C2-X_18–19_ -C3-X_2_ -C4. CSPs have higher amino acid sequence similarity than OBPs within the same species and between different species, confirming that CSPs are more conserved than OBPs [42]. Insect ORs have seven typical transmembrane domains. By comparing and analyzing the amino acid sequences of ORs, it has been found that the homology of ORs among different insects is very low, and ORs among the same kind of insects are also dispersed, which may be related to ORs’ participation in recognition of odorants [43]. This study supports the evidence from previous observations that ORCO has high homology among different species, and an insect usually contains only one ORCO [44]. Compared with ORs, IRs only have three transmembrane domains. However, the open reading frame of IRs is longer at more than 500, even up to 900, amino acids, but the ORs generally have fewer than 500 amino acids [45]. SNMP is the only protein expressed in neurons in CD36 family. Both the C-terminus and N-terminus of SNMPs have a transmembrane domain and an extracellular ring. MsanSNMP1 identified in this study had the above characteristics and good homology with the SNMPs in other aphids.

Chemosensory gene expression pattern analysis is an effective way to further analyze gene function. Previous studies have found that the antennae of alate and apterous aphids, such as *S. avenae*, *My. persicae* and *Schlechtendalia chinensis*, are significantly different in morphology and ultrastructure [46,47,48]. Only a few transcriptomic studies on the antennae of alate and apterous aphids found that the expression patterns of chemosensory genes were different in the antennae of different winged-type aphids [7]. Together with our results, these findings suggest that the morphology of antennae and the expression of related olfactory genes are differentiated for different winged-type aphids. As important chemical cues for chrysanthemum aphids to locate host plants, chrysanthemum volatiles were also found to significantly induce the expression of some chemosensory genes in this study. Moreover, alate aphids were induced to have more genes with significantly high expression, which proves to a certain extent that alate aphids are highly sensitive to the odorants of host plants so that they can accurately locate host plants when they migrate over a long distance.

## 5. Conclusions

Nineteen candidate genes encoding olfactory-related proteins were identified in the antennal transcriptome of *M. sanborni*. Phylogenetic analyses suggested that most of these olfactory-related proteins are conserved among aphid species. Several chemosensory genes of *M. sanborni* displayed wing- or odor-specific expression patterns, suggesting they may play unique roles in olfactory processes. The present study lays the groundwork for further functional studies of the olfactory system in chrysanthemum aphids and sheds light on a new perspective on semiochemical-based aphid management for the future.

## Data Availability

The data presented in this study are available in Supplementary Materials.

## Supplementary Materials

The following supporting information can be downloaded at: https://www.mdpi.com/article/10.3390/insects13070597/s1, Figure S1: Species distribution in the *M. sanborni* antennal transcriptome assembly; Figure S2: KEGG classification of *M. sanborni* unigenes; Figure S3: Multiple amino acid sequence alignment of candidate MsanOBPs; Figure S4: Multiple amino acid sequence alignment of candidate MsanCSPs; Figure S5: Multiple amino acid sequence alignment of aphids’ SNMPs; Table S1: Primers of *M. sanborni* chemosensory genes used for qRT-PCR; Table S2: Sequencing quality overview of antennal transcriptome of *M. sanborni*; Table S3: Assembly summary of *M. sanborni* antennal transcriptome; Table S4: DIAMOND annotation result statistics; Table S5: Sequence information and best blastx match information for candidate chemosensory genes; Table S6: Numbers and accession numbers of olfactory-related proteins from different aphid species; File S1: The amino acid sequences of 172 odorant binding proteins (OBPs) from 20 Hemiptera species; File S2: The amino acid sequences of 77 chemosensory proteins (CSPs) from 10 Hemiptera species; File S3: The amino acid sequences of 85 odorant receptors (ORs) from 11 Hemiptera species; File S4: The amino acid sequences of 39 ionotropic receptors (IRs) from nine Hemiptera species; File S5: The amino acid sequences of 19 sensory neuron membrane proteins (SNMPs) from 10 Hemiptera species; File S6: Transmembrane domain prediction for MsanORs.
