# Supplementary material for: Identification and Expression Analysis of Chemosensory Genes in the Antennal Transcriptome of Chrysanthemum Aphid Macrosiphoniella sanborni
Source: Insects. 2022 Jun 29;13(7):597. doi: 10.3390/insects13070597 (PMC9317607; doi:10.3390/insects13070597)
Supplement: Supplementary file 1 [file insects-13-00597-s001.zip › insects-1764546-SI.pdf]

Supplementary Materials:

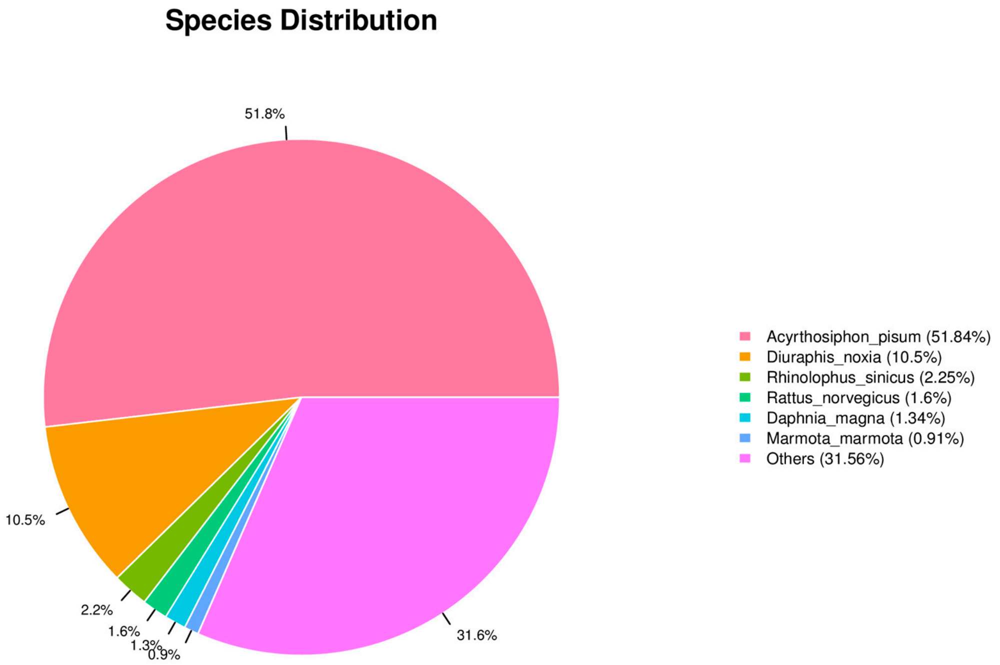

Figure S1. Species distribution in the *M. sanborni* antennal transcriptome assembly.

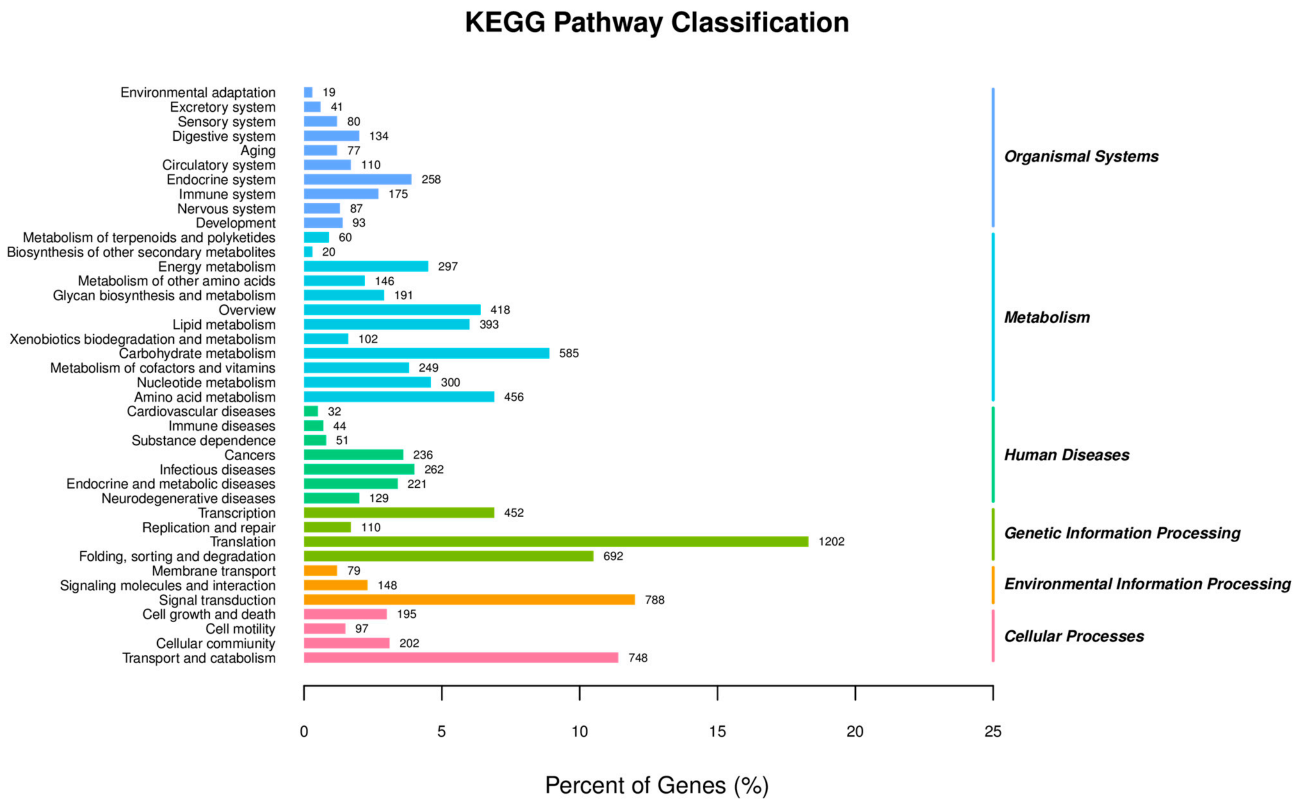

Figure S2. KEGG classification of *M. sanborni* unigenes.

```
MsanOBP9     ........................................    0
MsanOBP2     MKVSAATAVLVALVATVQSSDPCNISTCYKSGTTKPPIAV   40
MsanOBP4     ......................................MR    2
MsanOBP10    ........................................    0
Consensus

MsanOBP9     .................MIIKKTLLVSVFVIFGCLFSFNK   23
MsanOBP2     TPTHLPVQSSTPTNHPQTTYAKDHVHGSTTTKSGANATVT   80
MsanOBP4     GNYSLMVFLLFTIGLQDIYCQKQELSGKCRAPDKAPLNLE   42
MsanOBP10    .....................MEHLRSSNVVFAIVMALLV   19
Consensus

MsanOBP9     AADDADAADKE............................L   35
MsanOBP2     TASGASVNGTERPAVVKSSVGVTGNSTTPKPTMTEGHVAL  120
MsanOBP4     IIINICQEEIKS..........................AL   56
MsanOBP10    VQSSTRPQPDE............................I   31
Consensus

MsanOBP9     ISKLFTVVFKCFKDA..DWGTCGEMLTTKYDIAQAKYKQC   73
MsanOBP2     KQKLNTIAVKCKD....ELHAPQEIMALVSNTVVPQNEQQ  156
MsanOBP4     LQEALDILNDGNLEQNTPSYSSRSKREADEDLTNEERRVA   96
MsanOBP10    EEIKKTLYNACSG....KFPITEEIKNDFKNSIISDDPNF   67
Consensus

MsanOBP9     TCHLACAGEELGLINTSGQ.PEPAKFLEYVNRINNPGIKS  112
MsanOBP2     RCYLECVYKNLNLIKNNKFSVEDGKAMAKIRFANQPEEHK  196
MsanOBP4     GCLLQCVYKKVKAVDETGFPVVDGLMKLYNEGVQDRNYYM  136
MsanOBP10    KCFLRCCLDEMSLIDEDGI.IDGDSLKEMAIDSIKP....  102
Consensus     c l c

MsanOBP9     QLQLIYDKCQNVKGSE.............KCDLAEQFSIC  139
MsanOBP2     KAVTIIETCEKEAVIDPKTT........EKCAAGRVIRNC  228
MsanOBP4     ATLSAVRHCISIAQQLKQQQPSKSFDDGQTCDLAYEMFEC  176
MsanOBP10    IIEQVNSNCLKNVKQD.............GCQAAFEFISC  129
Consensus            c                     c        c

MsanOBP9     AFKESPAVSNNNYTSDGIII..                    159
MsanOBP2     FVKNGEKINFFPKA........                    242
MsanOBP4     VSEKIEENCGVENKSNNLSQRQ                    198
MsanOBP10    GIKLNPLTMQLLPL........                    143
Consensus
```

Figure S3. Multiple amino acid sequence alignment of candidate MsanOBPs.

```
MsanCSP1    ......MNLLAIFCYITVMCDTQFRRLEQPTTTPQVKRID     34
MsanCSP2    ......................RYFRFDQIYFCQNIYSLK     18
MsanCSP4    ......................MDSRIAVVCFVLAVFAVD     18
MsanCSP7    MARSSSSVTMKVFVIAVCVCAALARPEEAKMENKPTVVKS     40
Consensus

MsanCSP1    QPATIATRIGQATIAPRFGQVTAAPQIEQAAIASRIGQGF     74
MsanCSP2    QTKMAHLNLFVVLVASLV......................     36
MsanCSP4    QTVGAPQKDAVATGG.........................     33
MsanCSP7    ETLAAPLPTTIVKRATP.......................     57
Consensus

MsanCSP1    QNVNNSVSPTTDGRKTTRQTSSYPTRYDFIDIEAVMNNER    114
MsanCSP2    ..............CFTLAEEKYTTKFDNFDVDKVLNNDR     62
MsanCSP4    ....................SAYTNKYDHIDIDQVLASKR     53
MsanCSP7    .............QVVSTQQDSSLPNVSEDVLDKALSDRR     84
Consensus                                          r

MsanCSP1    IIKILFNCVMNQGPCTREGLELKWIVPDAIQTECAKCNER    154
MsanCSP2    ILTSYIKCLLDQGNCTNEGRELKRVLPDALKTDCSKCTGV    102
MsanCSP4    LVNSYVQCLLDKKPCTPEGAELRKILPDALKTQCAKCNAT     93
MsanCSP7    FVQRQLKCATGEGPCDPIGRKIKAHAPLVLRGMCVKCSQS    124
Consensus          c      c   g       p      c kc

MsanCSP1    QRKQAGKVLAHLLQYKPEYWNMLVKKFDPNNIYLRKYMVD    194
MsanCSP2    QKDRSEKVIKFLIKNRAADFDHLTAKYDPSGEYKKKLEKF    142
MsanCSP4    QKNAALKVVERLQRDYDKEWKQLLDKWDPKREQFQKFQQF    133
MsanCSP7    EIKQIQRVMSHIQKNYPKEYTKMLKQYQSGF.........    155
Consensus          v

MsanCSP1    NDDDEKLSLQKLTTNTT                           211
MsanCSP2    EAERIAAAKN.......                           152
MsanCSP4    LTEEKKKGGVVKF....                           146
MsanCSP7    .................                           155
Consensus
```

Figure S4. Multiple amino acid sequence alignment of candidate MsanCSPs.

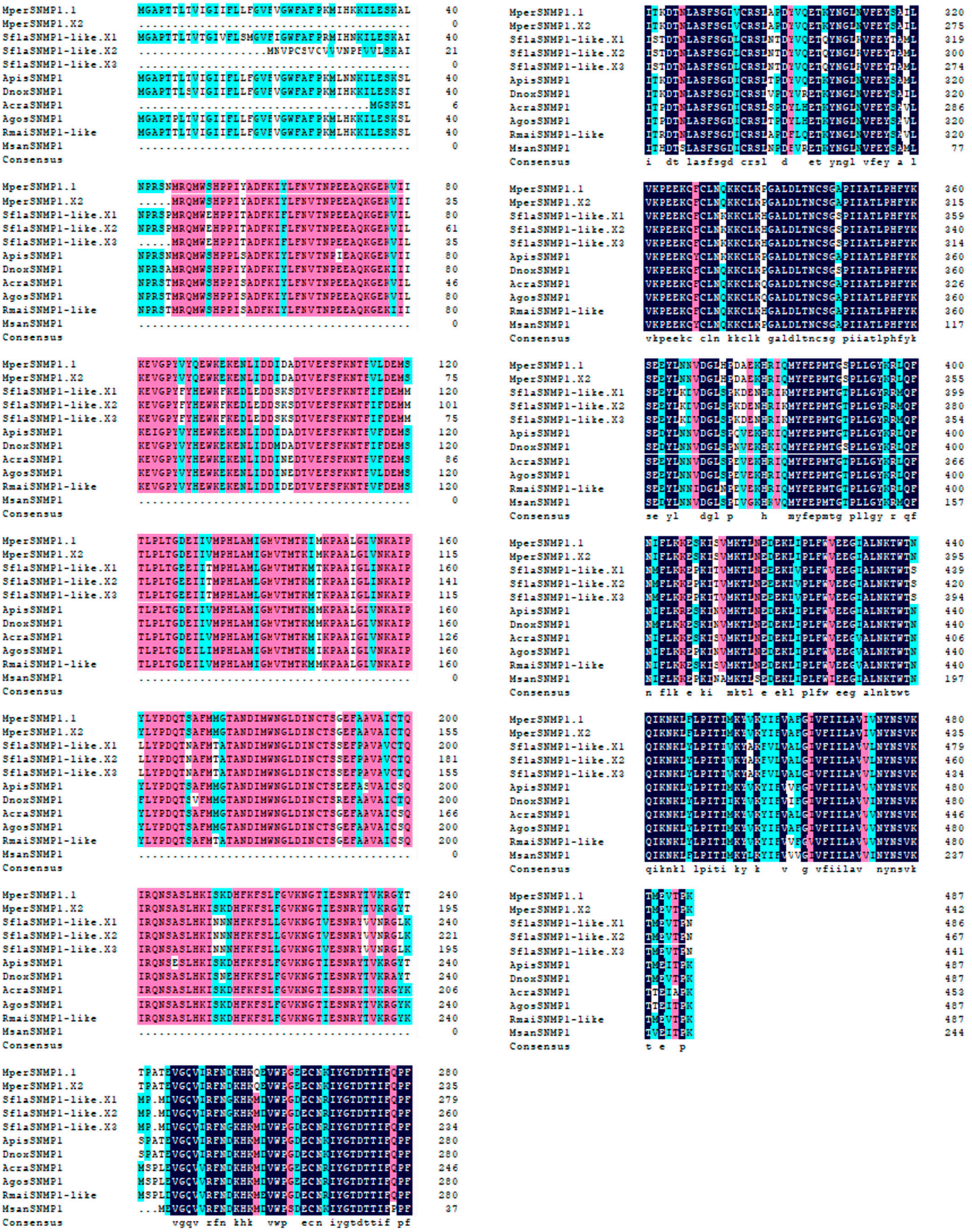

Figure S5. Multiple amino acid sequence alignment of aphids' SNMPs. Mper: *Myzus persica*; Afla: *Sipha flava*; Apis: *Acyrthosiphon pisum*; Dnox: *Diuraphis noxia*; Acra: *Aphis craccivora*; Agos: *Aphis gossypii*; Rmai: *Rhopalosiphum maidis*; Msan *Macrosiphoniella sanborni*.

Table S1 Primers of *M. sanborni* chemosensory genes used for qRT-PCR

Gene Name	Forward Primer (5'-3')	Reverse Primer (5'-3')
MsanOBP2	GCTCGGACCCATGTAACATATC	CTGTCGTGACCGTAGCATTAG
MsanOBP4	CGTAGAGTTGCAGGGTGTTTG	GAGGGCTGCTGTTGCTTTAG
MsanOBP9	CAGATGCAGCGGATAAGGAA	GTTCTGGTTGCCCTGAAGTA
MsanOBP10	CACGACCACAACCAGATGAA	GACATCTCGTCCAAGCAACA
MsanORCO	CAATGGGTCCTACGTGAATGAG	TGCTTGGTATGCGAGAATGG
MsanOR2	GTGGCCCGGTATGAGTTTAC	CATTCGTCACCTGGAGGTATTC
MsanOR4	CACTGTCACATCGGAGAAGT	GAAATCAACGCTCTGGATGC
MsanOR43b-1	CGCTGGTTATAGTCCCACTG	GACGCCCGTGAAATAGATGA
MsanOR43b-2	GGGATGGAGTGACGACCATA	GTGGACTACGATGAACAACCAC
MsanOR46a-1	AATCGCGACGGTTCAATCAG	TTGCCACTGACAGCGTATTG
MsanOR46a-2	CACGACGGTTCGATTGGATA	GACCTGCATCTGACAGCATA
MsanOR64	GGTAACCGACACAGACACATAC	CTGGCGGTAATACCCGATTG
MsanCSP1	CGACTTGAGCAACCGACTAC	CTGGAAACCTTGTCCGATCC
MsanCSP2	CGAAGGCCGAGAATTGAAGAG	CACCTGATGGGTCGTATTTGG
MsanCSP4	CACCAGAAGGAGCTGAACTTAG	GTTCACGTTTAGGGTCCCAT
MsanCSP7	CTACTCCTCAGGTCGTCTCTAC	ACTTGACGCACATTCCTCTC
MsanIR25a	GACCGTACTGAAACCCGAAG	CTCCTCCTTGCGGAGTAAATG
MsanIR40a	GGGTGGACGAGACGATAAAC	CACCAAATTGCGCTCGTATG
MsanSNMP1	ACGGGACAGATACCACGATA	AGGAGCACCTGAACAGTTTG
Msanβ-actin (Ref.)	GGATCGGTGGTTCCATTCTC	TCACAGCCTGCATCGTAATC

Table S2 Sequencing quality overview of antennal transcriptome of *M. sanborni*

Sample	Raw_Reads	Raw_Bases	Valid_Reads	Valid_Bases	Valid%	Q20%
AL1	51497638	7.72G	50069524	7.37G	97.23	98.71
AL2	49167846	7.38G	47948188	7.04G	97.52	98.59
AL3	57552466	8.63G	55919454	8.22G	97.16	98.69
AP1	55464352	8.32G	53863906	7.93G	97.11	98.76
AP2	57571092	8.64G	55611788	8.19G	96.60	98.71
AP3	57311562	8.60G	55917338	8.24G	97.57	98.80

Table S3 Assembly summary of *M. sanborni* antennal transcriptome

Index	All	GC%	Min Length	Median Length	Max Length	Total Assembled Bases	N50
Transcript	52249	42.14	201	449	15647	47335333	1656
Gene	28323	41.22	201	314	15647	21319147	1538

Table S4 DIAMOND annotation result statistics

Database	Annotated Gene Numbers	Ratio (%)
GO	11983	42.31
KEGG	6577	23.22
Pfam	11844	41.82
swissprot	9836	34.73
eggNOG	15144	53.47
NR	12042	42.52

Table S5 Sequence information and best blasts match information of candidate chemosensory genes

Unigene Reference	Gene Name	Length (nt)	ORF (aa)	Blastx Best Hit (Reference/Name/Species)	Evalue	Identity	TMD (No.)	Signal Peptide	Full Length
TRINITY_DN31343_c0_g1	MsanOBP2	1076	243	CAR85629.1/odorant-binding protein 2, partial [*Acyrthosiphon pisum*]	1.00E-154	96.43	0	19	Yes
TRINITY_DN7859_c0_g1	MsanOBP4	782	200	APB03427.1/odorant-binding protein 4 [*Sitobion avenae*]	4.00E-141	98.00	0	22	Yes
TRINITY_DN34070_c1_g8	MsanOBP9	763	160	AXE72026.1/OBP9 [*Megoura viciae*]	6.00E-89	88.89	0	24	Yes
TRINITY_DN35164_c1_g2	MsanOBP10	641	144	APB03433.1/odorant-binding protein 10 [*Sitobion avenae*]	6.30E-64	83.20	0	24	Yes
TRINITY_DN36123_c1_g6	MsanCSP1	1011	213	ULF48242.1/chemosensory protein 1 [*Acyrthosiphon pisum*]	1.00E-132	82.61	0	16	Yes
TRINITY_DN4112_c0_g1	MsanCSP7	843	156	ULF48248.1/chemosensory protein 7 [*Acyrthosiphon pisum*]	2.00E-107	98.06	0	24	Yes
TRINITY_DN36430_c0_g14	MsanCSP2	579	153	ULF48243.1/chemosensory protein 2 [*Acyrthosiphon pisum*]	3.00E-82	92.37	0	41	No
TRINITY_DN36313_c1_g3	MsanCSP4	535	147	AWV63292.1/chemosensory protein 4 [*Myzus persicae*]	6.00E-76	92.59	0	22	Yes
TRINITY_DN34819_c0_g2	MsanORCO	2503	464	XP_022162891.1/odorant receptor coreceptor isoform X1 [*Myzus persicae*]	1.30E-258	98.10	7	0	Yes
TRINITY_DN35161_c1_g4	MsanOR2	3164	434	XP_016662512.2/odorant receptor 2a-like [*Acyrthosiphon pisum*]	2.50E-182	82.91	6	0	Yes
TRINITY_DN37439_c1_g9	MsanOR43b-1	2375	400	XP_016660447.1/PREDICTED: odorant receptor 43b-like isoform X2 [*Acyrthosiphon pisum*]	2.00E-99	54.60	6	0	No
TRINITY_DN435_c0_g1	MsanOR4	1295	399	AQS60743.1/PREDICTED: olfactory receptor 4 protein	5.80E-117	76.20	6	0	No

				[*Acyrthosiphon pisum*]					
TRINITY_DN37765_c2_g1	MsanOR64	1281	391	UMT69240.1/PREDICTED: odorant receptor 64 [*Myzus persicae*]	4.00E-163	59.95	6	0	No
TRINITY_DN36837_c1_g2	MsanOR43b-2	1409	365	XP_016657134.1/PREDICTED: odorant receptor 43b-like [*Acyrthosiphon pisum*]	7.00E-100	54.70	6	0	Yes
TRINITY_DN34686_c1_g1	MsanOR46a-1	1758	329	XP_016660259.1/PREDICTED: odorant receptor 46a-like [*Acyrthosiphon pisum*]	1.50E-112	47.90	5	0	Yes
TRINITY_DN37775_c1_g4	MsanOR46a-2	969	323	XP_016660259.1/PREDICTED: odorant receptor 46a-like [*Acyrthosiphon pisum*]	1.30E-110	64.80	5	0	No
TRINITY_DN36559_c0_g1	MsanIR25a	2512	838	XP_022161260.1/ionotropic receptor 25a [*Myzus persicae*]	0.00E+00	93.76	3	22	No
TRINITY_DN37723_c2_g7	MsanIR40a	2403	739	XP_022182311.1/ ionotropic receptor 40a isoform X2 [*Myzus persicae*]	0.00E+00	96.36	3	52	No
TRINITY_DN36573_c2_g4	MsanIRDelta-1b	2769	527	XP_029347273.1/glutamate receptor ionotropic, delta-1-like [*Acyrthosiphon pisum*]	2.00E-284	93.14	3	0	Yes
TRINITY_DN37799_c1_g4	MsanIRDelta-1a	1175	368	XP_016663076.2/glutamate receptor ionotropic, delta-1-like [*Acyrthosiphon pisum*]	1.60E-190	92.10	3	0	No
TRINITY_DN35872_c3_g1	MsanSNMP1	3372	246	XP_003240759.4/sensory neuron membrane protein 1 [*Acyrthosiphon pisum*]	7.00E-150	90.57	1	0	Yes

Table S6 Amount and accession numbers of olfactory-related proteins of different aphid species

Species	OBP		CSP		OR		IR		GR		SNMP	
	**NO.**	**Accession**	**NO.**	**Accession**	**NO.**	**Accession**	**NO.**	**Accession**	**NO.**	**Accession**	**NO.**	**Accession**
Acyrthosiphon pisum	34	NP_001153534.1	16	NP_001119652.1	32	XP_029346749.1	11	XP_008183092.2	17	XP_003244120.4	1	XP_003240759.4
		NP_001353810.1		NP_001119651.1		XP_029346718.1		XP_029345195.1		XP_016660313.2		
		NP_001155983.1		NP_001119650.1		XP_029347684.1		XP_016662281.2		XP_029344591.1		
		NP_001153535.1		NP_001119649.1		XP_029347565.1		XP_029346904.1		XP_029344589.1		
		NP_001153533.1		NP_001128404.1		XP_029347069.1		XP_029347313.1		XP_029344230.1		
		NP_001153531.1		NP_001156200.1		XP_029347068.1		XP_029347065.1		XP_029343633.1		
		NP_001153532.1		ULF48242.1		XP_016662512.2		XP_029344228.1		XP_016657079.2		
		NP_001153530.1		ULF48243.1		XP_029342650.1		XP_029343909.1		XP_016662919.1		
		NP_001153529.1		ULF48244.1		XP_016657950.2		XP_029347699.1		XP_008185853.2		
		NP_001153528.1		ULF48245.1		XP_016663583.1		XP_029347698.1		XP_016659625.1		
		NP_001153527.1		ULF48246.1		XP_016661819.1		XP_016661577.1		XP_008186707.1		
		NP_001153526.1		ULF48247.1		XP_016660447.1				XP_008178128.1		
		NP_001153525.1		ULF48248.1		XP_016657134.1				XP_003248767.1		
		CAR85635.1		ULF48249.1		XP_008188009.1				XP_001942787.2		
		XP_029344816.1		ULF48250.1		XP_003245950.2				XP_029343507.1		
		XP_016663220.1		ULF48251.1		XP_001951646.2				XP_029343505.1		
		XP_008187140.1				ARJ54248.1				XP_029348406.1		
		XP_008187139.1				AQS60755.1						
		XP_008181869.1				AQS60754.1						
		XP_008181612.1				AQS60753.1						
		XP_008178459.1				AQS60752.1						
		CAX63070.1				AQS60751.1						
		CAX63069.1				AQS60750.1						
		CAX63068.1				AQS60749.1						
		CAR85637.1				AQS60748.1						
		CAR85636.1				AQS60747.1						
		CAR85634.1				AQS60746.1						
		CAR85633.1				AQS60745.1						
		CAR85632.1				AQS60744.1						
		CAR85631.1				AQS60743.1						
		CAR85630.1				AQS60742.1						
		CAR85629.1				AQS60741.1						
		CAR85628.1										
		ACI30694.1										
Aphis craccivora	1	CAR85658.1	0	-	37	KAF0766177.1	11	KAF0766232.1	68	KAF0773921.1	1	KAF0772486.1
						KAF0758873.1		KAF0773254.1		KAF0773181.1		
						KAF0751963.1		KAF0772645.1		KAF0767702.1		
						KAF0737851.1		KAF0772642.1		KAF0765041.1		
						KAF0765147.1		KAF0768064.1		KAF0762749.1		
						KAF0772142.1		KAF0767625.1		KAF0762692.1		

	KAF0770681.1	KAF0766106.1	KAF0762198.1
	KAF0766047.1	KAF0761777.1	KAF0761972.1
	KAF0766000.1	KAF0758016.1	KAF0760063.1
	KAF0765716.1	KAF0756485.1	KAF0759983.1
	KAF0763679.1	KAF0715507.1	KAF0755637.1
	KAF0763174.1		KAF0754673.1
	KAF0762590.1		KAF0754460.1
	KAF0762586.1		KAF0749867.1
	KAF0761946.1		KAF0747436.1
	KAF0754398.1		KAF0745194.1
	KAF0749570.1		KAF0735375.1
	KAF0748951.1		KAF0734115.1
	KAF0748431.1		KAF0729498.1
	KAF0767888.1		KAF0718170.1
	KAF0766732.1		KAF0715964.1
	KAF0766607.1		KAF0704079.1
	KAF0765148.1		KAF0773629.1
	KAF0764014.1		KAF0771009.1
	KAF0763175.1		KAF0771006.1
	KAF0763173.1		KAF0763562.1
	KAF0762588.1		KAF0762745.1
	KAF0759089.1		KAF0762387.1
	KAF0758875.1		KAF0762278.1
	KAF0758872.1		KAF0761922.1
	KAF0744260.1		KAF0760652.1
	KAF0742241.1		KAF0760064.1
	KAF0730385.1		KAF0759510.1
	KAF0730384.1		KAF0757315.1
	KAF0712197.1		KAF0751572.1
	KAF0706717.1		KAF0749859.1
			KAF0748601.1
			KAF0748336.1
			KAF0747775.1
			KAF0747416.1
			KAF0740675.1
			KAF0737845.1
			KAF0737167.1
			KAF0736755.1
			KAF0733013.1
			KAF0727737.1
			KAF0726823.1
			KAF0719565.1
			KAF0719564.1
			KAF0715541.1

										KAF0713603.1		
										KAF0710760.1		
										KAF0703014.1		
										KAF0698084.1		
										KAF0773723.1		
										KAF0773920.1		
										KAF0773636.1		
										KAF0765871.1		
										KAF0765870.1		
										KAF0765661.1		
										KAF0764140.1		
										KAF0755545.1		
										KAF0740068.1		
										KAF0715963.1		
										KAF0773180.1		
										KAF0763636.1		
										KAF0759334.1		
										KAF0749339.1		
Aphis fabae	2	CAR85657.1	0	-	0	-	0	-	0	-	0	-
		CAR85656.1										
Aphis glycines	23	QYL02823.1	0	-	0	-	0	-	0	-	0	-
		QYL02820.1										
		QYL02818.1										
		AHJ80894.1										
		QYL02822.1										
		QYL02821.1										
		QYL02819.1										
		QYL02817.1										
		QYL02816.1										
		QYL02815.1										
		QYL02814.1										
		QYL02813.1										
		QYL02812.1										
		AHJ80897.1										
		AHJ80893.1										
		AHJ80889.1										
		AHJ80888.1										
		AHJ80896.1										
		AHJ80895.1										
		AHJ80892.1										
Aphis gossypii	12	AGE97638.1	16	AGG38798.1	17	XP_027852915.1	8	XP_027852221.1	14	XP_027852364.1	1	XP_027838787.1
		AJP06027.1		AGG38799.1		XP_027844946.1		XP_027851996.1		XP_027851961.1		
		AGE97640.1		AGG38800.1		XP_027847983.1		XP_027849937.1		XP_027847490.1		
		AGE97639.1		AGG38801.1		XP_027844945.1		XP_027849936.1		XP_027847461.1		

		AGE97637.1		AGE97641.1		XP_027844943.1		XP_027844268.1		XP_027847431.1		
		AGE97636.1		AGE97642.1		XP_027842900.1		XP_027843243.1		XP_027846850.1		
		AGE97635.1		AGE97643.1		XP_027842896.1		XP_027840243.1		XP_027846833.1		
		AGE97634.1		AGE97644.1		XP_027841362.1		XP_027836912.1		XP_027845934.1		
		AGE97633.1		AGE97645.1		XP_027841183.1				XP_027845933.1		
		AGE97632.1		AGE97646.1		XP_027841162.1				XP_027840412.1		
		AGP04981.1		AGE97647.1		XP_027840127.1				XP_027840408.1		
		ACI30678.1		AGE97648.1		XP_027837639.1				XP_027840395.1		
				AGE97649.1		XP_027836930.1				XP_027839680.1		
				ACJ64045.1		XP_027853481.1				XP_027847075.1		
				ACJ64044.1		XP_027847498.1						
				ACJ64046.1		XP_027847411.1						
						AQS60756.1						
Aulacorthum solani	1	AHH34994.1	0	-	0	-	0	-	0	-	0	-
Brevicoryne brassicae	1	AEX65667.1	0	-	0	-	0	-	0	-	0	-
Cinara cedri	25	VVC24262.1	0	-	23	VVC25273.1	0	-	7	VVC27510.1	0	-
		VVC24261.1				VVC25250.1				VVC27540.1		
		VVC24260.1				VVC25231.1				VVC27552.1		
		VVC25032.1				VVC25230.1				VVC28649.1		
		VVC25280.1				VVC25910.1				VVC34142.1		
		VVC25238.1				VVC25909.1				VVC41288.1		
		VVC25306.1				VVC26167.1				VVC43122.1		
		VVC25514.1				VVC26166.1						
		VVC26092.1				VVC28590.1						
		VVC26168.1				VVC28589.1						
		VVC29378.1				VVC28588.1						
		VVC29376.1				VVC28587.1						
		VVC29375.1				VVC31899.1						
		VVC30360.1				VVC33585.1						
		VVC30725.1				VVC37787.1						
		VVC32662.1				VVC39717.1						
		VVC35381.1				VVC39903.1						
		VVC35805.1				VVC39900.1						
		VVC36643.1				VVC40829.1						
		VVC40159.1				VVC41125.1						
		VVC41790.1				VVC41882.1						
		VVC41776.1				VVC43746.1						
		VVC43406.1				VVC44361.1						
		VVC45546.1										
		VVC43934.1										
Diuraphis noxia	0	-	0	-	6	XP_015371514.1	0	-	12	XP_015375986.1	1	XP_015372007.1
						XP_015367780.1				XP_015375625.1		
						XP_015367779.1				XP_015369521.1		
						XP_015367764.1				XP_015369418.1		

						XP_015379800.1				XP_015369416.1		
						XP_015378374.1				XP_015369415.1		
										XP_015369414.1		
										XP_015369094.1		
										XP_015369092.1		
										XP_015369075.1		
										XP_015368969.1		
										XP_015367652.1		
Drepanosiphum	2	AEX65664.1	0	-	0	-	0	-	0	-	0	-
platanoidis		AEX65663.1										
Lipaphis erysimi	0	-	1	ABU56011.1	0	-	0	-	0	-	0	-
Megoura viciae	18	ALN66115.1	0	-	0	-	0	-	0	-	0	-
		4Z39_B										
		4Z39_A										
		AXE72022.1										
		CAR85653.1										
		AXE72027.1										
		AXE72026.1										
		AXE72025.1										
		AXE72024.1										
		AXE72023.1										
		AXE72021.1										
		AXE72020.1										
		AXE72019.1										
		AXE72018.1										
		CAX63260.1										
		CAR85652.1										
		CAR85651.1										
		CAR85650.1										
Melanaphis sacchari	1	XP_025196155.1	0	-	17	XP_025207156.1	7	XP_025204239.1	9	XP_025206784.1	2	XP_025193314.1
						XP_025205994.1		XP_025203212.1		XP_025205106.1		XP_025193313.1
						XP_025205992.1		XP_025196062.1		XP_025205086.1		
						XP_025205991.1		XP_025196061.1		XP_025204513.1		
						XP_025205601.1		XP_025193297.1		XP_025195890.1		
						XP_025205128.1		XP_025192137.1		XP_025195577.1		
						XP_025205100.1		XP_025195864.1		XP_025195576.1		
						XP_025204161.1				XP_025202850.1		
						XP_025200899.1				XP_025202779.1		
						XP_025200891.1						
						XP_025200888.1						
						XP_025200214.1						
						XP_025196562.1						
						XP_025195448.1						
						XP_025195447.1						

						XP_025200524.1						
						XP_025197380.1						
Metopolophium dirhodum	7	CAR85643.1	0	-	0	-	0	-	0	-	0	-
		CAX63256.1										
		CAR85642.1										
		CAR85641.1										
		CAR85640.1										
		CAR85639.1										
		CAR85638.1										
Myzus persicae	21	QLI60787.1	11	AWV63290.1	54	UMT69211.1	13	XP_022161260.1	14	XP_022180784.1	6	XP_022172547.1
		AWV63287.1		AWV63291.1		UMT69212.1		XP_022161203.1		XP_022180783.1		XP_022172546.1
		CAR85648.1		AWV63292.1		UMT69213.1		XP_022182312.1		XP_022180782.1		XP_022172545.1
		ACI30682.1		AWV63293.1		UMT69214.1		XP_022182311.1		XP_022179784.1		XP_022172544.1
		AWV63289.1		AWV63294.1		UMT69215.1		XP_022182310.1		XP_022172158.1		XP_022172543.1
		AWV63288.1		AWV63295.1		UMT69216.1		XP_022176431.1		XP_022165399.1		XP_022172542.1
		AWV63286.1		AWV63296.1		UMT69218.1		XP_022170527.1		XP_022165389.1		
		AWV63285.1		AWV63297.1		UMT69217.1		XP_022168779.1		XP_022162432.1		
		AWV63284.1		AWV63298.1		UMT69219.1		XP_022168778.1		XP_022161088.1		
		AWV63283.1		ACJ64048.1		UMT69220.1		XP_022168777.1		XP_022161087.1		
		AWV63282.1		ACJ64049.1		UMT69221.1		XP_022168776.1		XP_022161086.1		
		AWV63281.1				UMT69222.1		XP_022168775.1		XP_022183395.1		
		XP_022165015.1				UMT69223.1		XP_022168774.1		XP_022166919.1		
		CAR85649.1				UMT69224.1				XP_022166914.1		
		CAR85647.1				UMT69225.1						
		CAR85646.1				UMT69226.1						
		CAR85645.1				UMT69227.1						
		CAR85644.1				UMT69228.1						
		ACJ64043.1				UMT69229.1						
		ACI30684.1				UMT69230.1						
		ACI30683.1				UMT69231.1						
						UMT69232.1						
						UMT69233.1						
						UMT69234.1						
						UMT69235.1						
						UMT69236.1						
						UMT69237.1						
						UMT69238.1						
						UMT69239.1						
						UMT69240.1						
						UMT69241.1						
						UMT69242.1						
						UMT69243.1						
						XP_022160430.1						
						XP_022160649.1						

						XP_022162891.1						
						XP_022162892.1						
						XP_022162893.1						
						XP_022163466.1						
						XP_022164565.1						
						XP_022167526.1						
						XP_022167641.1						
						XP_022169050.1						
						XP_022169059.1						
						XP_022169061.1						
						XP_022169092.1						
						XP_022169093.1						
						XP_022169094.1						
						XP_022171404.1						
						XP_022173211.1						
						XP_022174880.1						
						XP_022175226.1						
						XP_022178692.1						
						XP_022180856.1						
Nasonovia ribisnigri	9	CAR85655.1	0	-	0	-	0	-	0	-	0	-
		ALB26778.1										
		CAX63259.1										
		CAX63258.1										
		CAX63257.1										
		CAR85654.1										
		4Z45_A										
		4Z45_B										
		4Z45_D										
Pterocomma salicis	5	CAX63261.1	0	-	0	-	0	-	0	-	0	-
		CAR85663.1										
		CAR85662.1										
		CAR85661.1										
		CAR85660.1										
Rhopalosiphum maidis	1	XP_026812533.1	0	-	26	XP_026820377.1	9	XP_026815367.1	14	XP_026819078.1	1	XP_026822399.1
						XP_026814691.1		XP_026819270.1		XP_026814267.1		
						XP_026814479.1		XP_026817182.1		XP_026813287.1		
						XP_026814179.1		XP_026817181.1		XP_026813286.1		
						XP_026807464.1		XP_026816263.1		XP_026812684.1		
						XP_026807368.1		XP_026814788.1		XP_026811182.1		
						XP_026807283.1		XP_026811161.1		XP_026807794.1		
						XP_026807240.1		XP_026810663.1		XP_026807230.1		
						XP_026807158.1		XP_026809176.1		XP_026806407.1		
						XP_026807122.1				XP_026822156.1		
						XP_026807026.1				XP_026821645.1		

						XP_026807025.1				XP_026821636.1		
						XP_026806824.1				XP_026821633.1		
						XP_026806786.1				XP_026821627.1		
						XP_026804939.1						
						XP_026804937.1						
						XP_026804936.1						
						XP_026804935.1						
						XP_026804934.1						
						XP_026822202.1						
						XP_026805470.1						
						XP_026805457.1						
						XP_026805444.1						
						XP_026805373.1						
						XP_026822384.1						
						XP_026817480.1						
Rhopalosiphum padi	5	AHL30243.1	0	-	0	-	0	-	0	-	0	-
		AHL30242.1										
		CAX63255.1										
		CAX63254.1										
		CAX63253.1										
Sipha flava	0	-	0	-	11	XP_025424050.1	9	XP_025415213.1	13	XP_025421193.1	3	XP_025419540.1
						XP_025421655.1		XP_025415212.1		XP_025419973.1		XP_025419539.1
						XP_025420356.1		XP_025420928.1		XP_025419807.1		XP_025419538.1
						XP_025420352.1		XP_025419699.1		XP_025417386.1		
						XP_025413921.1		XP_025419763.1		XP_025417128.1		
						XP_025412888.1		XP_025408296.1		XP_025417127.1		
						XP_025412887.1		XP_025408287.1		XP_025417126.1		
						XP_025412114.1		XP_025408278.1		XP_025412092.1		
						XP_025412107.1		XP_025408274.1		XP_025411658.1		
						XP_025406248.1				XP_025411657.1		
						XP_025413669.1				XP_025411656.1		
										XP_025410155.1		
										XP_025405505.1		
Sitobion avenae	28	ACW03675.2	7	APB03437.1	1	ACT37280.1	0	-	0	-	0	-
		AEX65668.1		APB03438.1								
		ACX32010.2		APB03439.1								
		ACY06265.1		APB03440.1								
		ACW03676.2		APB03441.1								
		APB03436.1		AFD20365.1								
		APB03435.1		AFD20367.1								
		APB03434.1										
		APB03433.1										
		APB03432.1										
		APB03431.1										

		APB03430.1										
		APB03429.1										
		APB03428.1										
		APB03427.1										
		APB03426.1										
		APB03425.1										
		APB03424.1										
		AER92708.1										
		AER92707.1										
		CAX63252.1										
		CAX63251.1										
		CAX63250.1										
		CAX63249.1										
		CAX63248.1										
		CAX63247.1										
		AER92706.1										
		AER92705.1										
Tuberolachnus salignus	1	CAR85659.1	0	-	0	-	0	-	0	-	0	-

File S1. The amino acid sequences of 172 odorant binding proteins (OBPs) from 20 Hemiptera species.
>SaveOBP1
MLNLKVMMFLCLSVTVVYCEIEENRLNNNTAIEICILETNIPKDEFQAMVTMPNNPDVDIL
TTRAQKCMLGCVMRKNHIINDGYVSTDVLYRYVMNFYGAVPNTKRKLLSRTVSKVIDICT
KKDNLPTEECVLADLIMTCVRSEALKRGLQR
>SaveOBP2
MKVSAATAVLVALVATVQSSDPCNISTCYKSGTTKPPMAVTPTRLPVQSSSTPTSHPQTTYA
KDHVHGSTTIKSGANATATTASGASVNGTERPIVVKSSAGVIGNSTTPKPTMTEGHVALKQ
KLNTIAVKCKDELHAPQEIMALVSNTVVPQNEQQRCYLECVYKNLNLIKNNKFSVEDGK
AMARIRFANQPEEHKKAVTIIETCEKEAIIDPKITEKCAAGRIIRNCFVKNGEKINFFPKA
>SaveOBP3
MISSTFYITSVFGIALLISCGYGRFTTDQIDYYGKACNASEDDLVVVKSYKVPSTETGKCL
MKCMITKLGLLNDDGSYNKTGMEAGLKKYWSEWSTEKIENINNKCYEEALLVSKEVVAT
CNYSYTVMACLNKQLDLDKST
>SaveOBP4
MRGNYSLMVFLLFAIGLQDIFCQKQEPSGKCRAPDKAPLNLEIIINICQEEIKSALLQEALDI
LNDGNLEQNTPSHSSRSKREADEDLTNEERRVAGCLLQCVYKKVKAVDETGFPVVDGLM
KLYNEGVQDRNYYMATLSAVRHCISIAQQLKQQQPSKSFDDGQTCDLAYEMFECVSEKIE
ENCGVENKSNNLSQRQV
>SaveOBP5
MSVNSATIKCIAVAVVLLQISIIFADAGHHRRGKELLDTEDSDFFRCKQASRKSCCGPENAM
KRFGDKDKVAADECYAQVAEKFATVTATTPKQDLFSAEAVKITKKKQFCLHECIGKKNNL
LTEDGSLNKTFIADYAMKSVFKEQWQKQVGQKALDKCLEETYIPWPAEDKENVCNPVYV
QFQHCLWLQYESNCPANKIKTTKKCEKTRNRYRMQKSTSN
>SaveOBP6
MQKVVFICIFAIICQTVFTAGYDRTWILRQKRMTNDDECRTLIPSPEKKLPSCCQMPDILPN
SNSTWEKCFETFKQFKDKPETKEYKEMAHGKEPPCLFQCIFMQSGLTTSDGKLNEDAITK
KMSEGINNDEKWKSTWQNSLNKCFDDVKQEDKKQIPIMNTPAGRLMKCFLRDMYMSCP
KNVWVESSECLNMKDLVQKCPEMPPPVFKSPPKLI
>SaveOBP7
MYNMLPKTVLFAIIAATVLKDCDAYLSEAAIKKTQQMLKTVCSKKFSVEEDVFTDIKKGIF
PEDNNNIKCYFACNFKTMQLINQKGSIDKKMFKDKMTMMAPPNVLKVLLPVIEQCTGID
KGEELCQSSYNLIKCAHTVDPRSLEYLPL
>SaveOBP8
MFALKVACLCLSVAVVFGENNQQNGPSDRSASIFQSCIAETKLSGDALKGFRSMSIPKTQA
EKCMMGCLMRKVNVINKGKFSVEEATKVAQKYYGTNEVMMKKAKDLIDVCAKKAQST
TEECALAGIVTTCIVEEAQKAGLAGGPGSRSRRTVSPKFRRDAM
>SaveOBP9
MIIKKTLLVSVFVLFGCLFSINKAADDADAGDKELMSKLFTVVLKCFKDADWGTCGEMIT
TKYDITQAKYKQCTCHMACAGEELGMINTSGQPEPAKFLEYVNKINHPSIKSQLQLIYDK
CHNVKGSEKCDLAEQFAICAFKESPALKERAATLMEMLVKMKPKSK
>SaveOBP10

MEHLRSTNVVFAIVMALLVVQSSTRPQPDEMEEIKKTLYNACAGKFPITEEIKKNAKNAIIS
DDPTFKCFLKCCFDEMSLIDEDGILDGDSMKAMAPDHIKPIFEQVIPSCLKNVKQDGCEAS
FEFISCGMKLNPLTVELLPL
>SaveOBP13
MDACTVHCVFNQLEMLNSNSRPDKYSIVNIMTNQIKDVELKEFIQDSIDECFDTLELDSNN
NKCEFSKNFAVCMENKAQRNCDDWDENLSANKINSAGLQDGTNQQDKRKGY
>AgosOBP2
MKVSAATAVLVALVATVQSSDPCNISTCYKSGTTKPPTTVTPTRLPVQSSSTPTSHQQTTYA
KDHVHSSTATKSGVNTTATTTSGASVNGTERTTVVKSSSGVAGNVTTPKPTMTDGHVALK
QKLNTIAVKCKDELHAPQEIMALVSNTVVPQNEQQRCYLECVYKNLNLIKNNKFSVDDG
KAMAKIRFANQPEEHKKAVTIIETCEKEAIIDPKTTEKCAAGRVIRNCFVKNGEKINFFPKA
>AgosOBP3
MISSTFYTSLMFGIAMLISCSFGRFTTEQIDHYGKACNATEDDLVIVKSYKVPTSDTGKCL
MKCMISKLGLLNDDGSYNKTGMEAGLKKYWSEWSTDTIESINNKCYEEALLVSKDIIATC
NYAYVVMACLNKQLKLDNST
>AgosOBP4
MRGNYSLVVFLLFGFGLLEIYCQKQELSGKCRAPDKAPLNLEIIINICQEEIKSALLQEALDI
LNDGTLEQNTPSYSRSKRDADEDLSNEERRVAGCLLQCVYKKVKAVDETGFPVVDGLMK
LYNEGVQDRNYYMATLSAVRHCISIAQQLKQQQPSKSFDDGQTCDLAYEMFECVSEKIEE
NCGVENKLNNLSQRQV
>AgosOBP5
MKMSANGATMKCVAVAVVLFQMSVIFAEAGHQRRGKELLDTEDSDFFRCKQASRKSCCG
PENAMKRFGDKDKVAADECYAQVAEKFATVTATTPKQDLFSGEAVKITKKKQFCLHECIG
KKNKLLTEDGSLNKTFIADYAMKSVFKEQWQKQIGQKALDKCLEETYIPWPAEETENKC
NPVYVQFQHCLWLEYESNCPDNKIKLTKKCEKTRNRYRMQKSPSNQ
>AgosOBP6
MQKVVFLCIFAIICQTVFTVGFERTWILRQKRMTNDNECRALFPSPEKKLPTCCQMPNILP
GLDNAWEVCFEKFKQFKDKHATKEYKEMVHENEPPCLFQCVFMQSGLTTSDGKVNEDA
VIKKMAEGMDNDEKWKSIWRNTFNKCLNDVKQEDKEQIKVMNTPTGRLMKCFLRDLY
MNCPKNVWVENSECSNLKDLVEKCPKLPPPVFQSPPKLI
>AgosOBP7
MNMLPATVLLAVVAATILKDSDAYLSEEAIKKTQKMLKNVCSKKHSVEEEVFTDIKKGIFP
ENNNNIKCYFACNFKTMQMVNQKGILDKKMFKDKMTMLAPPNVLAILLPPIEQCIGNDK
DTEICQSSYNFIKCAHRVDPKSLEFLPL
>AgosOBP8
MFAFKVACLCLSVAVVFGENNQQNSNDRSASIFQSCISETKLSGDALKGFRSMSIPKTQAE
KCMMGCLMRKVNVINKGKFSVEEATKVAQKYYGTNESMMKKAKDLIDVCAKKAQSTT
EECALAGIVTTCIVEEAQKAGLTGGPGSRSKRTVSPKFRHSIV
>AgosOBP9
MIIKKTLLVSGFVLFGCMFSINKAADDADTADKELMSKLITVAFKCFKDADWGTCGEMIT
TKYDITQAKYKQCTCHMACAGEDLGLINSNGQPEPAKFLEYVKRINNSVIKSQLQHIYDK
CQNVKGTEKCDLAEQFAICAFKESPEMKERVTKLIEMLVKMKPKSK
>AgosOBP10

MEHLRGTNVMFAIVMALLVVQSSTRPQPDEPDDIKKTLYNACSEKFPLTEEIKNNVKNSM
VIDDQNFKCFLRCCFDEMSLIDEDGIIDGESLAAMAVDKIKPVAEKIVHDCLPAGKQEKQD
GCEAAFKFFSCGMKLNPLTIELLPLQ
>MperOBP2
MKVSAATAVLVALVATVQSSDPCNISTCYKSGTTKPPMNVTPTRLPVQSSSTPTSHPQTTYA
KDHAHGSTTVKSGANATATTASGASVNGTERPAVAKSSAGVTGNSTTPKPTMTEGHVALK
QKLNTIAVKCKDELHAPQEIMALVSNTVVPQNEQQRCYLECVYKNLNLIKNNKFSVDDG
KAMARIRFANQPEEHKKAVTIIETCEKEAVIDPKTTEKCAAGRVIRNCFVKNGEKINFFPKA
>MperOBP3
MISSTFYITLLFGIAMLISCGYGRFSTEQIDYYGKACNASEDDLVVVKSYKVPTTETGKCL
MKCMITKLGLLNDDGSYNKTGMEAGLKKYWSEWSTEKIEAINNKCYEEALLVSKEVIAT
CSYTVMACLNKQLDLDKST
>MperOBP4
MRGNYSLTVFLLFVIGLQDIYCQKQEPSGKCRAPDKAPLNLEIIINICQEEIKSALLQEALDI
LNDGNLEQNTPSYSSRSKREADEDLTNEERRVAGCLLQCVYKKVKAVDETGFPVVDGLM
KLYNEGVQDRNYYMATLSAVRHCISIAQQLKQLQPSKSFDDGQTCDLAYEMFECVSEKIE
ENCGVENKSNNLSQRQV
>MperOBP5
MSANSATIKCIAVAVVLLQISVVFADAGHHRRGKELLDTEDSDFFRCKQASRKSCCGPENA
MKRFGDKDKVAADECYAQVAEKFATVAATTPKQDLFSADAVKITKKKQFCLHECIGKKNR
LLTEDGSLNKTFIADYAMKSVFKEQWQKQVGQKALDKCLEETYIPWPAEDKENVCNPVY
VQFQHCLWLQYESNCPDNKIKITKKCEKTRNRYRMQKSTSN
>MperOBP6
MQKVVFICIFAIIYQTVFTVGYERTWILRQKRMTNDDECRTLLPSSEKKLPSCCQMPNILPG
LDSTWEKCYEKFIQFKDKPETKEYKEMSHGKEPPCLFQCIFMESGLTTNDGKLNEDAITK
KMTEGINNDEKWKSTWKKSLDKCFDDVKQEDKKQILIMNTPAGRLMKCFLRDIYMNCP
ENVWVESSECLNVKNLVQKCPEMPPPVFQSAPKLI
>MperOBP7
MNNMIPATVLLAVIAATVLKDCDAYLSEAAIKKTQQMLKTVCSKKHSVEEDVFTDIKKGIF
PENNNNIKCYFACNFKTMQMINQKGTLDKKLFKDKMSMMAPPNIYNILLPAIEQCIGIDK
GEELCQSSYNFIKCAHRVDPKSLEYLPL
>MperOBP8
MFVLKVACLCLSVAVVFGENNQQNSSDRSATIFQSCIAETKLSGDALKGFRSMSIPKTQAE
KCMMGCLMRKVNVINKGKFSVEEATKVAQKYYGTNETMMKKAKDLIDVCAKKAQSTT
EECALAGIVTTCIVEEAQKAGLAGGPGSRSRRTVSPKFRRNSM
>MperOBP9
MLIKKTLLVSVFVLFSCLFSINKATDDADTADKELMSKLFTVVFKCFKDADWGTCGEMIT
TKYDITQAKYKQCTCHMACAGEELGLINSSGQPEPAKFLEYVNRINNPGIKSQLQHIYDKC
QNVKGTEKCDLAEQFAICAFKESPALKERATTLMEILMKMKPKSK
>MperOBP10
MEHLRSTNVVFAIVMALLVVQSSTRPQPDELEEIKKTLYNACAGKFPITEEMKKDILNSNM
VDDQNFKCFLRCCFDEMSMIDEDGIIDGESLISMATDNLKPVIQQVVQSCVKDIKQDGCE
AAFNFISCGLKLNPMTIQLLPL
>ApisOBP1

MLNLKVMMFLCLSVIVVYCESDQVPINSSAAVESCLLETNMTRDEFEDMLTSPNARELTIL
KSHAHKCMFGCVMRKNHIVNDGVVSKEVLSKYVLNFYGRPDYKRRLIIKDVEHIVDVCA
KKVADESETDECELAATLVTCIVLEANKAGLVDDPARQI*
>ApisOBP2
SDPCNISTCYKSGTTKPPMAVTPTHLPVQSSSTQTSHPQTTYAKDHVHGSTTTKSGVNATV
TTASGASVNGTEPPAVVKSSAGVTGNSTTPKPTMTEGHVALKQKLNTIAVKCKDELHAPQ
EIMALVSNTVVPQNEQQRCYLECVYKNLNLIKNNKFSVEDGKAMARIRFANQPEEHKKA
VTIIETCEKEAVIDPKTTEKCAAGRVIRNCFVKNGEKINFFPKA
>ApisOBP3
RFTTEQIDYYGKACNASEDDLVVVKSYKVPTTETGKCLMKCMITKLGLLNDDGSYNKTG
MEAGLKKYWSEWSTEKIESINNKCYEEALLVSKEVVATCNYSYTVMACLNKQLDLDKST
>ApisOBP4
QKQETSGKCRAPDKAPLNLEIIINTCQEEIKSALLQEALDILNDGNVEQNTPNYSSRSKREA
EEDLTNEERRVAGCLLQCVYKKVKAVDETGFPVVDGLMKLYNEGVQDRNYYIATLSAVR
HCISIAQQLKQQQPSKSFDDGQTCDLAYEMFECVSEKIEENCGVENKSNN
>ApisOBP5
DAGHHRRGKELLDTEDSDFFRCKQASRKSCCGPENAMKRFGDKDKVAADECYAQVAEK
FATVTATTPKQDLFSAEAVKITKKKQFCLHECIGKKNNLLTEDGSLNKTFIADYAMKSVFK
EQWQKQVGQKALDKCLEETYIPWPAEDKENVCNPVYVQFQHCLWLQYESNCPANKIKIT
KKCEKTRNRYRMQKSTSN
>ApisOBP6
PNILPNLDSTWEKCFETFKQFKDKPETKEYKEMAHGKEPPCLFQCIFMQSGLTTSDGKLNE
DAITKKMSEGINNDEKWKSIWQNSLNKCFDDVKQEDKKQILIMNTPAGRLMKCFLRDMY
MSCPKNVWVESSECLNMKDLVQKCPEMPPPVFKSPPKLI
>ApisOBP7
YLSEAAIKKTQQMLKTVCSKKHSVEEDVFTNIKKGIFPEDNNNIKCYFACNFKTMQLINQ
KGVIDKKMFKDKMSMMAPPNVYKILLPVIEQCTGKDKGEELCQSSYNVIKCAHSVDPKS
LEFLPL
>ApisOBP8
ENNQQNGPSDRSATIFQSCIAETKLSGDALKGFRSMSIPKTQAEKCMMGCLMRKVNVINK
GKFSVEEATKVAQKYYGTNEAMMKKAKDLIDVCAKKAQSTTEECALAGIVTTCIVEEAQ
KAGLSGGPGSRSRRTVSPKFRRDAM
>ApisOBP9
DDADAKDKELMSKLFTVVFKCFKDADWGTCGEMITTKYDITQAKYKQCTCHMACAGE
ELGMINASGQPEPAKFLEYVNKINNPDIKSQLQLIYDKCQNVKGSEKCDLAEQFAICAFKE
SPALKERVSTLMEMLVKMKPKSK
>ApisOBP10
STRPQPDEMEEIKRTLYNACAGKFPITEEIKNNAKNSIISDDPTFKCFLKCCFDEMSMIDED
GIIDGDSLKAMAPDHIKPILEQVIPSCTKNVKQDGCEASFEFISCGIKLNPLIVALLPL
>ApisOBP13
CTIHCVFNQLEMLNSNSRPDKYSIVNIMTNQIKDVELKEFIQDSIDECFDTLELDSHNNKCE
FSKNFAVCMENKAQRNCDDWDENLSANKINSAGLQDGTNQQDKRKGY
>PsalOBP2

MTEVHVALKQKLNTIAVKCKDELHAPQEIMALASNTVVPQNEQQRCYLECVYKNLNLIK
NDKFSVDDGKTMAKIRFAKQPEEYKKAVTIIETCEKEAVIDPKTTEKCAAGRVIRNCFVKN
GEKINFFPKA
>PsalOBP1
MNSSAAVENCLLETNMTRDEFEDMLTSPNARELTILKSHAHKCMFGCVMRKNHIVNDGV
VSKEVLSKYVLNFYGRPDYKRRLIIKDVEHIVDVCAKKVADESETDECELAATLVTCIVLE
ANKAGLVDDPARQI
>PsalOBP4
QKQETSGKCRAPDKAPLNLEIIINTCQEEIKSALLQEALDILNDGNTEQNTQNQSNRSKRE
TEEDLTNEERRVAGCLLQCVYKKVKAVDETGFPVVDGLMKLYNEGVQDRNYYIATLSAV
RHCISIAQQLKQQQPSKTFDDGQTCDLAYEMFECVSEKIEENCGVENKSNN
>PsalOBP9
MAKLFGVALKCFKDADWGACGEMITTKYDITEPKYKQCTCQMACVGEDLGMINTKGEP
EPAKFLEYVKRINNQSIKSQLQHIYDKCQNVKGADKCDLSEQFAICAFKESPALKERVSTL
MEMLVKMKPKSK
>PsalOBP10
STRPQPDELEEVKKSLYNACSSKFPLTEEIRNQAKNGILTEDPNLKCFLRCCFDEMSLIDED
GIIDGETLVAMSMDRIKLITEQAVHNCLKTTKQDGCEASFQFLSCGIKLNPLI
>AfabOBP8
ENNQQNSNDRSATIFQSCISETKLSGDALKGFRSMSIPKTQAEKCMMGCLMRKVNVINKG
KFSVEEATKVAQKYYGTNETMMKKAKDLIDVCAKKAQSTTEECALAGIVTTCIVEEGQK
VGLTGGPGGRSRRTVSPKFRRNSM
>AfabOBP2
SDPCNISTCYQSGTTKPPTTVTPTRLPVQSSSTPTSHQQTTYAKDHVHSSTATKSGVNTTAT
TTSGASVNGTERTTVVKSSSGVAGNLTTPKPTMTEGHVALKQKLNTIAVKCKDELHAPQEI
MALVSNTVVPQNEQQRCYLECVYKNLNLNKNNKFSVDDGKAMAKIRFANQPEEHKKAV
TIIETCEKEAIIDPKTTEKCAAGRVIRNCFVKNGEKINFFPKA
>AcraOBP2
SDPCNISTCYKSGTTKPPTTVTPTRLPVQSSSTPTSHQQTTYAKDHVHSSTATKSGVNTTAT
TTSGASVNGTERTTVVKSSSGVAGNATTPKPTMTEGHVALKQKLNTIAVKCKDELHAPQEI
MALVSNTVVPQNEQQRCYLECVYKNLNLIKNNKFSVDDGKAMAKIRFANQPEEHKKAVT
IIETCEKEAIIDPKTTEKCAAGRVIRNCFVKNGEKINFFPKA
>TsalOBP1
MNSSAAVENCLLETNMTRDEFEDMLTSPNARELTILKSHAHKCMLGCVMRKNHIVNDGV
VSKEVLSKYVLNFYGRPDYKRRLIIKDVEHIVDVCAKKVADESETDECELAATLVTCIVLE
ANKAGLVDDPARQI
>MvicOBP1
MTRDEFEDMLTSPNARELTILKSHAHKCMFGCVMRKNHIVNDGVVSKEVLSKYVLNFYG
RPDYKRRLIIKDVEHIVDVCAKKVADESETDECELAATLVTCIVLEANKAGLVDDPARQI
>MvicOBP2
MSVTPTRLPVQSSSTPTSHPQTTYAKDHSHGSTTTKSGANATATTASGASVNGTERPAVVK
SSAGVTGNLTTPKPTMTEGHVALKQKLNTIAVKCKDELHAPQEIMALVSNTVVPQNEQQR
CYLECVYKNLNLIKNNKFSVEDGKAMAKIRFANQPDEHKKAVTIIETCEKEAVIDPKTTEK
CAAGRVIRNCFVKNGEKINFFPKA

>MvicOBP3
MASMRFTTEQIDYYGKACNASEDDLVVVKSYKVPSSETGKCLMKCMITKLGLLNDDGSY
NKTGMEAGLKKYWSEWSTEKIESINNKCYEEALLVSKEVIATCNYSYTVMACLNKQLDL
DKST
>MvicOBP5
MKRFGDKDKVAADECYAQVAEKFATVTATTPKQDLFSADAVKITKKKQFCLHECIGKKNH
LLTEDGSLNKTFIADYAMKSVFKEQWQKPVGLKALEKCLEETYIPWPAEDKENVCNPVY
VQFQHCLWLQYESNCPANKIKITKKCEKTRNRYRMQKSTSN
>MvicOBP8
MSIPKTQAEKCMMGCLMRKVNVINKGKFSVEEATKVAQKYYGTNETMMKKAKDLIDVC
AKKAQSTTEECALAGIVTTCIVEEAQKAGLSGGPGSRSRRTVSPKFRRNSM
>MvicOBP10
STRPQPDELEEIKKTLYNACAGKFPITEEVKNNAKNSIFLDDQNFKCFLKCCLDEMSLIDD
DGIIDGDSLKAMASDKIKPMXEQVVPNCLKNVQQDGLEAAFVFLRXGRG
>MdirOBP1
MTRDEFEDMLTSPNARELTILKSHAHKCMFGCVMRKNHIVNDGVVSKEVLSKYVLNFYG
RPDYKRRLIIKDVEHIVDVCAKKVADESETDECELAATLVTCIVLEANKAGLVDDPARQI
>MdirOBP2
MAVTPTRLPVQSSSTPTSHPQTTYAKDHVHGSTTIKSGANATATTASGASVNGTERPTVVK
SSAGVIGNSTTPKPTMTEGHVALKQKLNTIAVKCKDELHAPQEIMALVSNTVVPQNEQQR
CYLECVYKNLNLIKNNKFSVEDGKAMARIRFANQPEEHKKAVTIIETCEKEAIIDPKTTEK
CAAGRVIRNCFVKNGEKINFFPKA
>MdirOBP3
RFTTEQIDYYGKACNASEDDLVVVKSYKVPSTETGKCLMKCMITKLGLLNDDGSYNKTG
MEAGLKKYWSEWSTEKIENINNKCYEEALLVSKEVVATCNYSYTVMACLNKQLDLDKST
>MdirOBP4
QKQETSGKCRAPDKAPLNLEIIINICQEEIKSALLQEALDILNDGNLEQNTPASYSSRSKREA
DEDLTNEERRVAGCLLQCVYKKVKAVDETGFPVVDGLMKLYNEGVQDRNYYMATLSAV
RHCISIAQQLKQQQPSKSFDDGQTCDLAYEMFECVSEKIEENCGVENKSNN
>MdirOBP5
DAGHHRRGKELLDTEDSDFFRCKQASRKSCCGPENAMKRFGDKDKVAADECYAQVAEK
FATVTATTPKQDLFSAEAVKITKKKQFCLHECIGKKNNLLTEDGSLNKTFIADYAMKSVFK
EQWQKQVGQKALDKCLEETYIPWPAKNKENVCNPVYVQFQHCLWLQYESNCPANKIKIT
KKCEKTRNRYRMQKSTSN
>MdirOBP6
PNILPNSNSTWAKCFETFKQFKDKPETKEYKEMAHGKEPPCLFQCIFVQSGLTTSDGKLNE
DAITKKMSEGINNDEKWKSTWQNSLNKCFDDVKQEDKKQILIMNTPAGRLMKCFLRDM
YMSCPKSVWVESSECLNMKDLVQKCPEMPPPVFKSPPKLI
>MdirOBP8
ENNQQNSNDRSATIFQSCISETKLSGDALKGFRSMSIPKTQAEKCMMGCLMRKVNVINKG
KFSVEEATKVAQKYYGTNGTMMKKAKDLIDVCAKKAQSTTEECALAGIVTTCIVEEAQK
AGLSGGPGSRSRRTVSPKFRRNSM
>NribOBP3

RFTTEQIDYYGKACNASEDDLVVVKSYKVPSTETGKCLMKCMITKLGLLNDDGSYNKTG
MEAGLKKYWSEWSTEKIETINNKCYEEALLVSKEVVATCSKSHDRKACLNQDPDLDKST
>NribOBP2
MTVTPTHLPVQSSSTPTSHPQTTYAKDHVHGSTTTKSGANATATTASGASVNGTERPAVVK
SSAGVTGNFTTPKPTMTEGHVALKQKLNTIAVKCKDELHAPQEIMALVSNTVVPQNEQQR
CYLECVYKNLNLIKNNKFSVEDGKAMARIRFANQPEEHKKAVTIIETCEKEAVIDPKTTEK
CAAGRVIRNCFVKNGEKINFFPKA
>NribOBP5
DAGHHRRGKELLDTEDSDFFRCKQASRKSCCARKNAMKRFGDKNKVAADECYAQVAEK
FATVPATTHKQDLFSAEAVKITKKKQFCLHECIGKKNNLLTEDGSLNKTFIADYAMKSVFK
EQWQKEVGQKALDKCLEETYIPWPAEDKENVCNPRYVQIQHCLWLLSRRNIPAHKSKITK
KCEKTRNRYRMQKSTSN
>NribOBP7
YLSEAAIKKTQHMLKTVCSKKHSVDEDVFTEIKKGIFPEDNNDIKCYFACNFKTMQLVNQ
KGYIDKKLFKDKMSIMAPPNVYNILLPVIEQCAGIDKSEELCQSSYNLIKCAHRVNPKSLEF
LPL
>NribOBP8
ENNQQNSNDRSATIFQSCISETKLSGDALKGFRSMSIPKTQAEKCMMGCLMRKVNVINKG
KFSVEEATKVAQKYYGTNETMMKKAKDLIDVCAKKAQSTTEECALAGIVTTCIVEEGQK
AGLTGGPGGRSRRTVSPKFRRNSM
>RpadOBP2
SDPCNISTCYKSGTTKPPTTVTPTRLPVQSSSTPTSHQQTTYAKDHAHSSIAAKSGANVTAT
TASGATVNGTERPTVVKSSPGVAGNATTPKPTMTVEHVALKQKLNTIAVKCKDELHAPQEI
MALVSNTVVPQNEQQRCYLECVYKNLNLIXNNKFSVDDGKAMARIRFANQPEEHEKAVT
IIETCEKEAIIDPKTTEKCAAGRVIRNCFVKNGEKINFFPKA
>RpadOBP3
MISPTFYISLLFSIGMLISCSFGRFTTEQIDHYGKACNASEDDLVIVKSYKVPTSDTGKCLM
KCMISKLGLLNDDGSYNKTGMEAGLKKYWSEWSTDTIENINNKCYEEALLVSKDVVATC
NYAYVVMACLNKQLKLDKST
>RpadOBP5
DAGHHRRGKELLDTEDSDFFRCKQASRKSCCGPDNAMKRFGDKDKVAADECYAQVAEK
FATTKATTPKQDLFSSEAVKVTKKKQFCLHECIGKKNKLLTEDGSLNKTFIADYAMKSIFK
EQWQKQIGQKALDKCLEETYIPWPAEETENKCNPVYVQFQHCMWFEYESNCPSNKIKLT
KKCEKTRNRYRMQKSTSN
>RpadOBP7
MNMLPATVLLAVIAATVLKDSDAYLSEAAIKKTQQMLKNVCSKKHSVGEDVFTDIKKGIF
PENNNNIKCYFACNFKTMQMINPKGILDKKMFKDKMTMLAPPNVLEILLPAIEQCIGTDK
DTEICQSSYNFIKCAYRVDPKSLEFLPL
>RpadOBP10
STRPQPDEMEEIKKTLYNACSAKFPLTDEIRNNAKNSIVADDQNLKCFLRCCFDEMSMIDE
DGIIDGESLVSMTSDKLKIVAKKAVDSCLTADKQDGCEAAFKFISCGIKLNPLIGSATL
>AglyOBP2
MKVSAATAVLVALVATVQSSDPCNISTCYKSGTTKPPTTVTPTRLPVQSSSTPTSHQQTTYA
KDHVHSSTATKSGVNTTATTTSGASVNGTERTTVVKSSSGVAGNVTTPKPTMTDGHLALK

QKLNTIAVKCKDELHAPQEIMALVSNTVVPQNEQQRCYLECVYKNLNLIKNNKFSVDDG
KAMAKIRFANQPEEHKKAVTIIETCEKEAIIDPKTTEKCAAGRVIRNCFVKNGEKINFFPKA
>AglyOBP3
MISSTFYTSLMFGIVMLISCSFGRFTTEQIDHYGKACNATEDDLVVVKSYKVPTSDTGKCL
MKCMISKLGLLNDDGSYNKTGMEAGLKKYWSEWSTDTIESINNKCYEEALLVSKDIIATC
NYAYVVMACLNKQLDLDKST
>AglyOBP4
MRGNYSLVVFLLFGFGLLEIYCQKQETSGKCRAPDKAPLNLEIIINICQEEIKSALLQEALDI
LNDGTLEQNTPSYSRSKRDADEDLSNEERRVAGCLLQCVYKKVKAVDETGFPVVDGLMK
LYNEGVQDRNYYMATLSAVRHCISIAQQLKQQQPSKSFDDGQTCDLAYEMFECVSEKIEE
NCGVENKSNN
>AglyOBP5
MKRFGDKDKVAADECYAQVAEKFATVTATTPKQDLFSGEAVKITKKKQFCLHECIGKKNK
LLTEDGSLNKTFIADYAMKSVFKEQWQKQIGQKALDKCLEETYIPWPAEETENKCNPVYV
QFQHCLWLEYESNCPDNKIKLTKKCEKTRNRYRMQKSTSN
>AglyOBP6
MQKVVFLCIFAIICQTVFTVGFERTWILRQKRVTNDDECRTLIPSSEKKLPTCCQMPNILPG
LDNAWEVCFEKFKQFKDKHATKEYKEMAHGNEPPCLFQCVFMQSGLTTSDGKVNEDAVI
KKMAEGMDNDEKWKSIWRNTFNKCLNDVKQEDKEQIKMTNTPTGRLMKCFLRDLYMN
RPKNVWVESSECSNLKDLVEKCPKMPPPVFKSPPKLI
>AglyOBP7
MVARKRMYMLPATVLLAVVAATILKDSDAYLSEEAIKKTQKMLKNVCSKKHSVEEEVFT
DIKKGIFPENNNNIKCYFACNFRTMQMVNQKGILDKKMFKDKMTMLAPPNVLAILLPPIE
QCIGNDKDTEICRSSYNFIKCAHRVDPKSLEFLPL
>AglyOBP8
MFAFKVACLCLSVAVVFGENNQQNSNDRSASIFQSCISETKLSGDALKGFRSMSIPKTQAE
KCMMGCLMRKVNVINNGKFSVEEATKVAQKYYGTNETMMKKAKDLIDVCAKKAQSTT
EECALAGIVTTCIVEEAQKAGLTGGPGSRSKRTVSPKFRHSIV
>AglyOBP9
MIIKKTLLVSGFVLFGCMFSINKAADDADAKDKELMSKLITVAFKCFKDADWGTCGEMIT
TKYDITQAKYKQCTCHMACAGEDLGLINSNGQPEPAKFLEYVKRINNSVIKSQLQHIYDK
CQNVKGTEKCDLAEQFAICAFKESPEMKERVTKLIEMLVKMKPKSK
>AglyOBP10
MEHLRGTNVVFAIVMALLVVQSSTRPQPDELDDIKKTLYNACSEKFPLTEEIKNNVKNSIVI
DDQNFKCFLRCCFDEMSLIDEDGIIDGESLAAMAVDKIKPVAEKIVHDCLPAGKQEKQDG
CEASFKFFSCGIKLNPLTIELLPLQ
>LeryOBP3
RFTTEQIDYYGKACNASEDDLAVVKSYKVPSTETGKCLMKCMITKLGLLNDDGSYNKTG
MEIGLKKYWSEWSTEKIEAINNKCYEEALLVSKEVVATCNYSYTVMACLNKQLDLDKST
>LeryOBP7
MVARKRMYNMLPTNVLLTIIAATVLNDCDAYLSEAAIKKTQQMLKSVCSKKYTVEEDVF
TNIKKGIFPEDNNNIKCYFSCVFKTMQMINQKGSLDKKIFKEKMSMMAPPSVYNILLPAIE
QCIGKDNGEELCQASYNFIKCAHHIDPKSLEFLPL
>DplaOBP3

MISSTFYITSVFGIAMLISCGYGRFTTDQIDYYGKACNASEDDLVVVKSYKVPSTETGKCL
MKCMITKLGLLNDDGSYNKTGMEAGLKKHWSEWSTEKIENINNKCYEEALLVSKEVVAT
CNYSYTVMACLNKQLDLDKST
>BbraOBP3
MISSTFYITLLFGIAMIISCSYGRFTTDQIDYYGKACNASEDDLVVVKSYKVPSTETGKCLM
KCMITKLGLLNDDGSYNKTGMEIGLKKYWSEWSTEKIEAINNKCYEEALLVSKEVIATCN
YSYTVMACLNKQLDLDKST
>AlinOBP1
MNSLIPVLLVVCAAATRADEQTNAMVAKAFNKCREEFPISDDEIGGVREKTTIPESHNAKC
LMACMLREGKMLRDGKYEKENALIMADVLNKDDPASADKAKQLVETCAGKVGTDAGG
DECEFAYKMAVCAAEEAKKLGVRPPDF
>AlinOBP2
MSLKIQFFVFAAICAACVCAYQEQLKQTIRDCQDGKEVTDDELEEFTKPLIPRNREEKCIM
ACVMRTYNIISNGHYDPKIAFGILKGILKDHPEKLNKIKEVMDHCGEDVPSHMDDECDLA
GEIMQCEVKYQKAMGMA
>AlinOBP3
MDIRFGFIIACLAILSVANAISKEYSARMIAAKEKCQKEFNVTDSVVEDFMKRNIKPESKSG
KCMVHCIMEEMGMIDDHKINTEQVKLGNKEKWDDPALVELANQVADTCDQEVFTEGRC
KCLVAVEYMMCLATHGDEVGLPHVDFEDSQDS
>AlinOBP4
MRIFVIFTAALTCVMAGELPEEMKEMAQGLHDSCVEETGVDNGLIAPCAKGNFADDAKL
RCYFKCVFGNLGVISDEGELDAEAFGSILPDSMQELLPTIKSCGGTTGSDPCDLAMNFNKC
LQKADPVNFLVI
>AlinOBP5
MVLKMNLLLVVLVMSQVFFSVTEAAMSQAQMKQAMKTVRNMCIPKSGVDKEALAKMV
NGEFDESDQKLKCYLGCVLGMMQAVKNNKINLTMVRNQITKMLAPERGQRILAAFESCA
TVTGDDNCGLAFRFAKCIYDTDKEAFIVP
>AlinOBP6
MGFKFVKYRSYFFVLVIHIILCIQIKAKELTDEQKEQIFAEIKNCMESTKLTDEEFESIMAKK
ELPTSKEGKCFTKCLMEKMEYLEEGGKINVIAVQAGMEENMEKESEITKAKEIIQQCADS
VPPEDSCEYAYGISQCMYNKMKEAGISGS
>AlinOBP7
MNRPLLLLTAVLTVGSGQQEDCKTAPAGWPRRPPQCCDLPFPLEGMKKEFGSCIRQIGNRQ
SSAVPTAQAVRDARLCIEECVYKGLGFMDEHKLNKDQLLEQLKKGIADKKDWTKPMEGA
VKRCHETITKRETPQEAACQDSAHEFTHCAMRELFLNCPASEWNNNDECNLVKSRMQAC
PNIPPPPPPPPQGFRGQGPPPQ
>AlinOBP8
MDTHFGLLIASLAILHTANAVINKDYLEKVVTAKDKCLKEFNVDDSVVEDFIVKYNKPQS
ESGKCMVACFMEERGMMKDGKTITEQVMLDNQEKWIAATHVNMGKEVIDTCDKEVPNE
ENDKCDLAVDYMMCLVKRGDEAGLPKMDVAQLKH
>AlinOBP9
MMELWKWRLALIIFGLVSCIQQTEGSQRTKQQPKSKTKESVVGATRPRDAKATECVNKVN
ANEEESASFFRKEIPETEAGKCLLACYLEGKGLIVGGKISSSGAARVAARAYPNNRVKTGN
VKHILSHCGTIAGRESNNCEMAYKLADCTTTLSDKFRL

>AlinOBP10
MFFNSVFLLVVCVSSYVTKGQELPPPGDVKNKTVVFKNSFLRSAKYCSSIYETSTLAIMAL
LMSEKSDDQNGKCFLNCMLQRYRLMSQDGSYNKDKFKPFLEYIPDSKFLQSIRGNLKNCI
SEKDPDPCEKASKFIKCFYTRARNKGEIGASKEVIPADGF
>AlinOBP11
MKTFVGLIFAVALVEFASAISKEYHDKAIEAKNTCAKLHNVDDETIMTYWKNHQLPEKEP
ETCIVICYLKEMKLVVDGKVDADAWKASNKEKWDDEKHVAAADEIVDKCSAEVPPTEN
ECEWGLALTKCALKHGKEAGIPPPDMEHPKRR
>AlinOBP12
MTTKLRSIGLVFIVSISYAFAYQELLKETIKKCQNGRDVTDDEVEEFTKPLVPKNEEERCLV
ACVFKEYKVIIDGHFDPVNALNVAKVVYKDYPDKVERIKDVLDHCGEDIPTHNDNECDL
AGDIMKCEVKYLNSVPKMTSLEFLAGSMAATAEP
>AlinOBP13
MNISTRMISLTMAYLAAALVSGHRALDGILPQANQDECREESNFRGELNDDVGRNVTQEL
KCFAACSLMKLGIMNEKDGTVNMTRLDELIASHTPGKDAADVFKTTVVEPCMKEVKKST
DYCEYSYQLIACGMSKVP
>AlinOBP14
MKPPGPPGSGTPPTAEERAARKIAHECADECLYKSSNLLTSAGELDKDAIKALVTKLYTGD
WATAATTAIDKCLASAKGEVEATSKCKSGSFQLSRCFMRSMFLGCPASSWTESTECAAAK
ARLTKCPNAMAPMPHKK
>AlinOBP15
MFAITAALSLLLLNVAIAHPGHFDEDPECRPPHHHRLEEKDCCKIPNLFSKSKDEMHELVH
KCFEEAGIKKHGHHDHHGPPPPPGLGLPPPPPPPPKNDSKFDCVEQCFLKNLELINEEGEV
KVDELKALIAEKFTGDWASVGSSAIEKCLEKSKTEENDSTKCKAGSKRILICLARESFLSCP
ASEWTESDVCTAAKDRLEKCPHAPPPMNH
>AlinOBP16
MKFFASAALVLLVAAAVKANEKKANEKVTEIFNKCKETWPVTDEEIEQVKQKNSIPESKN
VKCILACMLKEAKVLKDGEYNKDNAELMADVLYKDEPEHAEKSKQIIELCSAELGTKTD
GDDCEYAYKMSVCAAKHAKELGVKTPEF
>AlinOBP17
MQAAFVLLGAALLVAVVSGAPPSVKEIVQNVSKKCAAETKASPEQAKIILTQNIPKNDVER
CYLQCVYSGVGVIKDGKFSQEGGNKLVAMRFHDAKEKELAKQLINTCAKEIKAKDGEKC
SLGKGIRQCFVAHGKEVNFFPHA
>AlinOBP19
MNTLLLCAVIAVSACFAYDFSDPEFNIIIDDELFDIAEGKDTLLRNRRDIDDDDERMAFEQD
EADDPSNGPPDHLSEMNDENSHHKGHCKRHHKSCCGKTPLPLSLIQHGKNETKRSTGSEC
YEEIDAKMGNKTSLENDMDPYNCEKVKRMKKKQYCMHECKAKKMGVANEQGVLDFP
KVKDLLLSRVNETWQKEVLGQAVDTCATSKFDQTWKDDQDEYKCNPQALQFKHCVWK
QVELKCPEEYQNTGRHCKKLRTKLTSETNKETATKETTI
>AlinOBP20
MYTFNTFFVLTVASYVIAAPPADVPAECLVPKGKEEEVSKCCKLDAISTKEVAAAKECMKL
VKDKDAESKGLLPKPEGFDCFDDCVMTKMGFMGADKKIDAAKVNAGAKTSYTGDWAE
PGAKMVAKCLAQVSAMKEKEECASGADIYETCIFRESYMNCPEKSWTNSDACKANKERL
TKCPKALPYLEKLDLS

>AlinOBP21
MNPTVAIISIFFVAYTQAHTKELHATEVYKLKVRDECNKDIKATPEQSEIVSKFKEVPKDET
EKCLLECIYIKTGGIDADGKYSVEGFNKLIDMKYKGDENTNAKKINTDCAKKAVSKEGEK
CSLGGSIRECFAAAAKENDFFTI
>AlinOBP22
MATNAKAVLFLALCGIVYVSAYQEVLRATISDCKGGKEVSQEELDEFIKPLIPQTREEKCL
MACVFTAYNVIVEGHFDPKLAYGVAKNILHENPEKLKHIKETLDYCGHEIPTKMDDECEL
ASEVMACRNKYNKDHGYDQDP
>AlinOBP23
MNTFAALVLVASVVALSQGNPTTGAPSTTLVSDQPTGSTASGVSKSPEEIKQKIKEQVGALT
EACKSQSKITGEQAKIVATQAIPKTEAEKCFLECIYTGLQLTKDGKFNEPAARALAQKRFG
NAPDDLQKANSMITACVKEVVVKDTNEKCALGRLIRECFVKNGAKINFFPKP
>AlinOBP24
MPTFSTLSTFLFALALTYGQIVEDPECRPPHPPGREDSCCILPELFIGGIQDVIRKCHDEAGL
KKPSGPHGGGSPPSAEEMAAHWRAHECADECVLKSQNLLTTDGELNKDAIKAQVVKSFT
GDWAKLASDIADKCLASAKGEVTSTATCKSGAGQFIYCFRRNLFLQCPSSSWTETTDCAA
AKARITKCPNAKIPMGHHRH
>AlinOBP25
MDVMILPVFLIVFIAATASPAVLSAECPQRFPKEMRPLSSCCKVEMNPNSSYNSTAGEAIID
KCFGGFNSSATRPTGPPSGYDCEMECLMIEFGFMGKDKTINKDKIVTSIQDEYSADFQEAA
NKAIEICMGRKYQTSCPSGIDGMMECFAVQMMLNCPAKHWSGGEDCKETKQLIEKCGEV
LSIFADYDTE
>AlinOBP26
MKSLWKCCKTVQSSKATHPTSEQMVEIKSCYSNWNHSVETNAAPEGFDCVEECVYSKLG
FMGTDKTINKEKLLQFQKEETHEDFHEAITKSMDMCMGKTFTTKCPSGIDAVIKCEAIQIY
LNCPAKHWDNGDDCQETKKLMEKCADVTSMYN
>AlinOBP27
MTQWKTASFFVAMAALVVIAFAGLPFQNEMAVMQCKTKFDVTSEDIQLLKDRKLPASHS
GKCMMACILKKMKVMTKRGQFDLRNVQKWLRNKYQGDQANLAKGTYVAEACANILPT
LGIQDECEMAAEIMSCVRNKSKLIKKTADGQLPSSTGI
>AlinOBP28
MSSDMANILLLLVIGAGIAVNEGDASMEALMECKKDYKVSREQIMSGDSSEEVKCFAECL
MKKTGGMDEGGNFNTEKIKEEGRKHAKTDDQKRANDAAVDKCISETEAANPTGKCEKG
FEFFKCVRGEMKSPM
>AlinOBP29
MYQLTCPVFVIVMYWVNQGSADVGLLDYSAQCVTEAGVTHDEADKIQQGNLPSNQQGK
CYVACVLKSLGLVDRRGKISAENTNRLIDMYSNEPGDAKDKTKQAVNTCATEANRAWTW
SQCEVAYRMMSCILRTRGSLQQTRSITITLPQSITINPPPLSFTLFSVG
>AlinOBP30
MQLKNTSDSLVGHLFSIALPNTLLNPSQLLPELAITSRQSPDIVSIACFLHAIGSNFLFASKTP
SEANRQSWMHSNSLESVPLVSFESQVVIVLLVKGNT
>AlucOBP1
MCSKYFVMLIGLTVYTSAEVINEECKDRNQSSTEYETFYNCCDLESSFNETKSKEKEEARE
FCENEFEKANNVSEDEAEPSPSSVRQDCYVDCILKKLGAMSEDYKMDKEKVTKWFMEG

THKDFEEVGKQAMEKCYDKTYSKKHCASRVMGLLWCYSEELVMNCPAKYWDQSEKCT
AAKAYMKKCSTNPWRSED
>AlucOBP2
MRSTGSECFEEIDAKLGNKTSLESDMDPYNCEKVKRMKKRHYCMHECKAKKLGVATEE
GNLEFPKVKELLLSRVNETWQKDILGQAADTCATSKFDQTWKDDTEEYKCNPQALQFKH
CVWKQVEMKCPEEHQNTGRHCKKLRSKISSETSKDIAKETSV
>AlucOBP3
MFSSATLVCLFAVALTQGQLDEDPECRPPHPPGKDDKCCTIPELIVGENMQAMMKQCFEES
GMERRPPGPPGSGTPPTPEEIEAHRSAHECVDECFFKAAKFMNSDGEFDLEAMKTAAASV
FTGDWAPLGSETIDKCFASAKSQVSASAKCTSGAHRAKKCILRNFIINCPPSAWNDSTDCA
ALKARLTKCSNAMPPFPHHKH
>AlucOBP4
MEVAACLVLLAALAALTAAVEEGRPLCKAPTTAPRKLEKVINQCQEEIKYALLQEAPSVLG
ETVGLKTALTRNRSKRETFTGEERRIAGCLLQCVYRKMKALDETGFPTATGLVKIYSEGVE
DRNYYLATIQGVQRCLSRELQSRNTNPSIVKAEGYSCDVAYDMFNCVSEQIEQLCGTSP
>AlucOBP5
MNSIIVLCLVASAVTLSQGNPTTPNPSTSHVSSSAGITVSGVSKSPEEIKLKIKEQVATLTGAC
KTQTKLTGEQAKIVASQAIPKTEAEKCFLECIYQGLQLTKDGKFNEPAARAWAQKRFGNA
PEDLQKANTMIDICVKEVVVKDENEKCALGRLIRECFVKNGAKINFFPKP
>AlucOBP6
MYDRFKLFALLALVVSCKSAPPEEPAECKLPESDSAELVKCCKLNVVLDEMADSVGECM
KLVKGKPEKGPPVPEGFDCMDTCVFSKLGFAANNKLDAEKLTKKFSELFKGDWSALSDS
TLKKCLPMAEGAKGSCASGADVFKFCIVRELYMNCPASSWTKSDLCKANVERLEKCPHS
MPFLPGTGIKKN
>AlucOBP7
MNPLILILLVVFAAATRGEEQANALVAKAFNKCFGEFPLGDDEMKEVKDKSTVPSSHNAK
CLMACMLKEGRILRGGKYELENAILMADVLNKNDHAATDKAKQLIETCAAQVGTDASA
DECEFAYKMALCASDEAKKLGVRPPDF
>AlucOBP8
MVLKMKQILVVFVALQVLISTTEAVMTQAQMKQAMKTVRNMCIPKSGVDKEALAKMVE
GEFDESDQKLKCYLGCVLGMMQAVKNNKINLTMVKNQISKMLAPEQGQRILAAFEGCAT
VTGDDNCDLAFKFAKCIYDTDKELLFQAFIVP
>AlucOBP9
MKSFVGLIFAVALVEFASAITKEYHDRAVAAKDACLKKHPSIKESDVQEFLKKHKLPETDD
GKCMIACYMEEMNLMADGKINVEEAKKTNSDKYDGEPDNKELADKLIDHCSSQVSPDG
MSKCEYAYQISKCGLEYGMKNGLTPPKMYEEQRR
>AlucOBP10
MTYHVFFRKFDLPRISRRVRQCYYHSVPRSLSGSSRRMLEETSQHHPKRRSRVSEKHKLPE
TDDGECMIACYMEEKNLMADGKINVKEANQTNSDKYDGEPDNKQLAEKLIDHCSSQVS
PDGMSKCEYAYQFSKCGLEYGMKNGLTPPKMYEEQRR
>AlucOBP11
MGSQYERTLVGVRYLPIMKRVKFILVLSLLSRCSSAPTDDMAACMQITNEDSASMATCCD
YVIPFSNKTMTTCDKKETSGEMSKEFECVQDCLFSSDNVLGADKKFDPVAWRKHATNTIS

GDWKGVIANSGSNCEGFKKVLAQSMEKKCPTSESDVSFNCMTLQWYMNCPKSAWTSSE
SCEASKKKLMSCFGPIFENTS
>AlucOBP12
MTCSHFIALLSVVALSLSSGEINEECKDIENLKTQLENFYGCCDFESMIERVVRTEEEVETD
RFCREERKKINSTDGKVPLASEGHDCFMECVLKRMGAMGQDFKFIREKLDDFFLRGYPE
EVKQAGKLAFDKCLSKNFSKKYCASGINGLMMCLPEELVMNCPANIWSSHESCPIAKEAI
KKCPSYRVMIEQE
>AlucOBP13
MKHSSCVVPVALTIFVVAIVSGFKELDDVLPKPKQDECRKESNFQAELPSDINQNITQELKC
FAACSLVKLGLMNEKDGTINMAQLEDLIAKHTGGKDAADMFKHTVVEPCMKEVNKTTD
YCEYSFQLVKCGMSKVKPPSTGTEG
>AlucOBP14
MALNAKAVLLLGVCGLVYVSAYQEVLKATLKDCKGGKEITQEEVDEFMKPLIPKNEEER
CLMACVFRAYNVIVDGHFDPKLAYGVAKNILHENPEKLKHIKETLDYCGHEIPTKMDNEC
DLAGEVMSCRNKYNIDHGYDQDP
>AlucOBP15
MMRPTAYYLFASYAALLVCVHFASVSAITPELDKRAKAAVAKCADVPRTDEAKKEDCHA
GCFMSAMGYMTNGEINVKNMEEANKQKWDDQEIIKKGIQVDTTCAKQVGDTKGKSECT
IGYEFSTCKKELVKKVGLPPPTPLKE
>AlucOBP16
MKRLVFVLFTLCSLQWVSGITDELKQKAQAARLTCKQQVGLSDKEFNDWVKGIALPTTD
GGTCCEVCACWMRELGYLTGGRVNLENMKAVNAQKWNNLAYVELGNKIDALCSDRVL
QTGRKECEIAVDFRKCKTELIQQFGGPPKPGST
>AlucOBP17
MRILVLFTAALTCVLAGELPEEMREMAQGLHDSCVGETGVDNGLIAPCAKGSFADDPKLK
CYFKCVFGNLGVISDDGELDAEAFASILPDNMQALLPTIRGCGSTTGADPCDLAMNFNKC
LQKADPVNFMVI
>AlucOBP18
MHAAIVLIGSALLVAYVSGAPSANVKEIVQNVSKKCAAETKASPDQAKIVLSKNIPKDDAE
RCFLQCVYTGVGVIKDGKFSEEGGKKLVALRFHDAKEKELANKLIATCAKEIKAKDGEKC
SLGRAVRECFVNHGKQVNFFPSA
>AlucOBP19
MNSRFGIVFASLALLHITNAGNIKEGYVAKIAEIKDKCLKEHNVDHSVVEDLLKKSIKPEV
KAAQCMVACFFEENGMMKDGKIVSEMVKSNNAHQYEDPADVEKANEASDMCDGEVST
DGKDKCLLAADYALCWVKRTEEAGLPQIDFANSS
>AlucOBP20
MYTFKTFFVLTLASYVIAAPPADEPAECKPMKEKEEEISKCCKLEPVTVKEQAAFVDCMK
LVKDTDKKGPPKPEGFECLDDCILSKTGSLGSDKKIDPAKINAAAKTTYTGDWAEPGAKM
VEKCLAQVAENKDKTVCSTSGADVYTKCIFRESYINCPEKSWTNSDACKANKERVIKCPK
TLPYNAEQHKAETR
>AlucOBP21
MKFFVVSAALVLLVAAAVKANEKKANEKVTEIFNKCKETWPVTDEEIEQVKQKQSIPDSK
NVKCILACMLKEAKILRDGEYNKDNAELMADVLYKDEPEHAEKSKQIIEMCSSELGTKTE
GDDCEYAYKMSVCASKHAKELGVKTPEF

>AlucOBP22
MSLKIHFFVFAAIGAACVCAYQDQLKQTIKDCQGGKEVTDEELEEFTKPLIPKNEEERCIM
ACVMRTYNIINNGHYDPKIAFGIIKGILKDHPEKLDRIKEVMDHCGEDVPQHMDNECDLA
GEIMQCEVKYQKAMGLN
>AlucOBP23
MYVFTVALSFALLNIVFTHPGHFDEDPECRPPHSHRHEEKECCKTPNLFSKNKDEMHELV
HKCFEEAGIKKPHHGHHGPPPPGDEPPPPPPPPFHSKNNTKFECVEQCFLKNLELIDEEGDL
KIDDFKALVGEKYTGDWASVGSAALEKCLEKTKTEEKESSKCKAGSKHVLLCIARESFIN
CPASDWTESEVCSDAKERVVKCPDIPPPMNH
>AlucOBP24
MSTKLRSVGMILAIAITHVCAYQEQLKETIKQCQDGREVTDDEVEEFTKPLVPKNQEERCL
VACVFKEYKVIIDGHFDPVNALNVAKMVYKDYPEKWKRIKDVIDHCGEDIPTHNDNECD
LAGDIMNCEVKYLNSMPKGVSLELLAGSIAATAEP
>AlucOBP25
MFTSTIFAVFLFSVALTQGQMDDDPECRPPPPPNKEGSCCTVPRLLDNADKPEVIKKCHDE
AGMKRPSGPPGSGTPPTAEEMAAHKSAHECADECIFKSSNLLKSDGELDQDAIKATTTKM
FTGDWSTIASTAVEKCLATAKSEVGASAKCKSGAHQMVKCFARTMFLNCPASSWTESTEC
AAAKTRLTKCPNAMPPPPHHSRH
>AlucOBP26
MNPTVAIIFTLLVAYVKANTKELSPSEALKQKVKVQCQQEVKATPEQLKIYDNFKDVPKD
DVENCLMECMYTKTGGIGADGKYSVEGFKKLVDMKYKGEENTKARKIAADCEAKAAP
KEGEKCSMGRAIRECLAAATKENEFFTI
>AlucOBP27
MARKFIKSCYTLVALLVFVGSIHVEAKELTEEQRTQLFEDLKQCKNSTDLSDDEFETIIAKK
ELPTSEAGKCFTKCLMEKLDIIEDAEGGKKKISVITMQASLEENMEKEDDIAKGKDIIQKC
GDTVEPEDSCAYAYNISKCIYDRMKEAGISQ
>AlucOBP28
MIIEIICVLTVGISPHFIEGQELPPPGGVGNKTAVFKESFIRTAKYCSSIHETSTVAVLAILMSE
ESDDQNGKCFLNCMLQRYQLMSKQGAYNKDKFKPFLDYIPESRFLQSIKGNLKTCITERD
PAPCEKAYKFIKCFYTRARNKDEFGKIQRK
>AlucOBP29
MNRPLLLLTAVLAVGSGQQEDCKTAPAGWPRRPPQCCDLPFPLEGMKKEFGSCIRQIGNR
QSSAVPTAQAVRDARLCIEECVYKGLGFMEEHNLNKDQILQQLTKGVADKKDWTKPMED
AVKSCHETITKRETPQEGTCKDSAHEFTHCVMRQLFLSCPASEWNNNDECNLVKSRMQA
CPNIPPPPPPPPQGFRGQGPPPPQ
>AlucOBP30
MNAHIVLCLVASVFALSQGTPTTPTPATSRRVTVAPEDLEQAKSLRKFCTAKTGFTGITTTE
TSKGKDQARTATTPRPKTQLEKCYLECLYTGLQLTKDGKFNEPGARALANKRYKNAPEEL
RKVNSIIDFCITEVVVRDIEEMCALGRLIKECFSKYGAKNFPEL
>AlucOBP31
MFTSATFTVFLFAVTLTRGQIDEDPECRPSGPPGKEPECCTIPMKLFGDEVQEAVVKNCFDE
AGMKRPSGPHGGGSPPTAEEMAAHISAHECADECVFKSGNFIKSDGGLDEDAIKAVIAKL
FTGDWAPIATAAVNKCLASAKSGVSASAKCKSGAYQLSKCFQRELFLGCPASLWTESTDCS
AIKARITKCPNAKVPIGHHHKH

>AlucOBP32
MSGRHSLILVLLAAVTSAEVLTDGDCPKTMPKEMKPLYKCCVVEMDSNKTISDDQKAAV
DSCVNTSKSDSDANKHDCMIECIFIKLGYMGEDKTINVDYVLKEMNSLLPEDFHEQTSKS
LATCMGKKFSSTECPSEIDGVMACFSTMVLMNCPAKHWTDDEECKATRKFFQKCGDSIG
YRYD
>AlucOBP33
MHPWKTTCLIGMTAALMVVTAFAGLPFQNEMAVMQCKVKFDVTAEDIQLLKDSKLPSSH
SGKCMMACILKKMKVMTKRGQFDLRNVQKWLRNKYQGDQANLAKGNYVAEACANTL
PTLGIQDECEMAAEIMTCVRTKSKLVKKTLNGELPKEVSP
>AlucOBP34
MEHWKWRLALLIFGMVTCVPQLEGAQKSKQPSKAKTKESQVVAARPKDARAAACVTQI
GPDEEEEASFYRKEIPETDKGKCLLACYLESKGVLSGGKFSSSGAAKIAARAYPNNAAKT
GNVKHILSHCGTIAARETEQCQLAYRLAECTTTLADKFKL
>AlucOBP35
MINVVFVLLIGTGIVSGGFMEALIECKQQHHVSKEEAMTGESEEVKCFSECVLKKSGMMS
DNNEFDEEKIQAEGARMIKNDEQKNREFEGAAKACIEKVNGENPSEKCAKGHALFKCMK
EAMPMSKMRG
>AlucOBP36
MKTFVGLIFAVALVEFASAVSKEYHDKAIAAKNTCAKLHNVDDETIMKFWKAHQLPEKEP
ETCIIICYMKEMKLVVDGKVDADAWKASNKEKWDDEKHVAAADEIVDKCSAEVPPTENE
CEWGLALTKCALKHGKEAGIPPPDMEHPKRR
>AlucOBP37
MDTHFGLLIASLAILHTANAVINKDYLEKVVTAKDKCLKEFNVDDSVVEDFIVRYNKPQS
ESGKCMVACYMEERGMMKDGKTITEQVMLDNQEKWIAATHVNMGKEVIDTCDKEVPN
EKNDKCDLAVDYMMCLVKRGDEAGLPKMDVAQLKH
>AlucOBP38
MGFKFVKYRSYFFVLVIHIILCIQIKAKELTDEQKEQIFAEIKNCMESTKLTDEEFESIMAKK
ELPTSKEGKCFTKCLMEKMEYLEEGGKINVIAVQAGLEENMEKESEITKAKEIIQQCADTV
PPEDSCEYAYGISQCMYTKMKEAGISGGP
>AsutOBP1
MNSLIPVLLVVCAAATRADEQTNAMVAKAFNKCREEFPISDDEIGGVREKTTIPESHNAKC
LMACMLREGKMLRDGKYEKENALIMADVLNKDDPASADKAKQLVETCAGKVGTDAGG
DECEFAYKMAVCAAEEAKKLGVRPPDF
>AsutOBP2
MSLKIQFFVFAAICAACVCAYQEQLKQTIRDCQDGKEVTDDELEEFTKPLIPKNREEKCIM
ACVMRTYNIISNGHYDPKIAFGILKGILKDHPEKLNKIKEVMDHCGEDVPSHMDDECDLA
GEIMQCEVKYQKAMGMA
>AsutOBP3
MATNAKAVLFLALCGIVYVSAYQEVLKATISDCKGGKEVSQEELDEFIKPLIPQTREEKCL
MACVFTAYNVIVEGHFDPKLAYGVAKNILHENPEKLKHIKETLDYCGHEIPTKMDDECEL
ASEVMACRNKYNKDHGYDQDP
>AsutOBP4

MRIFVIFTAALTCVMAGELPEEMKEMAQGLHDSCVEETGVDNGLIAPCAKGNFADDAKL
RCYFKCVFGNLGVISDEGELDAEAFGSILPDSMQELLPTIKSCGGTTGSDPCDLAMNFNKC
LQKADPVNFMVI
>AsutOBP5
MGHIPMSSDMTNILLLLVIGAGIAVNEGDASMEALMECKKDFKVSREQIMSGDSSEEVKC
FAECLMKKTGGMDEGGNFNTEKIKEEGRKHAKTDDQKRAHDAAVDKCISETEAANPTG
KCEKGFEFFKCVRGEMKSLM
>AsutOBP6
MGFKFVKYRSYFFVLVIHIILCIQIKAKELTDEQKEQIFAEIKNCMESTKLTDEEFESIMAKK
ELPTSKEGKCFTKCLMEKMEYLEEGGKINVIAVQAGLEENMEKESEITKAKEIIQQCADTV
PPEDSCEYAYGISQCMYTKMKEAGISGGP
>AsutOBP7
MNRPLLLLTAVLTVGSGQQEDCKTAPAGWPRRPPQCCDLPFPLEGMKKEFGSCIRQIGNRQ
SSAVPTAQAVRDARLCIEECVYKGLGFMDEHKLNKDQLLEQLKKGIADKKDWTKPMEGA
VKKCHETITKRETPQEAACQDSAHEFTHCVMRELFLNCPASEWNNNDECNLVKSRMQAC
PNIPPPPPPPPQGFRGQGPPPQ
>AsutOBP8
MKLALVTAFLSAIVLAEGNINKEYLDKLIAAKEKCVKEFSVDDSIVEDLYVRYNKPPTESG
KCMVACYMEERGMMKDGKTITEQVMLDNQEKWIAATHVNMGKEVIDTCDKEVPNEEN
DKCDLAVDYMMCLVKRGDEAGLPKMDVAQLKH
>AsutOBP9
MMELWKWRLALIIFGLVSCIQQTEGSQRTKQQPKSKTKENVVGATRPRDAKATECVNQV
KANEEESASFFRKEIPETEAGKCLLACYLEGKGLIVGGKISSSGAARLAARAYPNNRVKTG
NVKHILSHCGTIAGRESNNCEMAYKLADCTTTLSDKFKL
>AsutOBP10
MFFNSVFLLVVCVSSYVTKGQELPPPGDVKNKTVVFKNSFLRSAKYCSSIYETSTLAIMAL
LMSEKSDDQNGKCFLNCMLQRYRLMSQDGSYNKDKFKPFLEYIPDSKFLQSIRGNLKNCI
SEKDPDPCEKASKFVKCFYTRARNKGEIGASKEVIPADGF
>AsutOBP11
MKTFVGLIFAVALVEFASAVSKEYHDKAIAAKNTCAKLHNVDDETIMKFWKAHQLPEKEP
ETCIIICYMKEMKLVVDGKVDADAWKASNKEKWDDEKHVAAADEIVDKCSAEVPPTENE
CEWGLALTKCALKHGKEAGIPPPDMEHPKRR
>AsutOBP12
MFQAFVYQELLKETIKKCQNGRDVTDDEVEEFTKPLVPKNEEERCLVACVFKEYKVIIDG
HFDPVNALNVAKVVYKDYPDKVERIKDVLDHCGEDIPSHNDNECDLAGDIMKCEVKYLN
SIPKMTSLEFLAGSMAATAEP
>AsutOBP13
MGDVSNTYGSYDSYGASYGGYIRGSTPSGSGRGYKQSYEGDIGSGTSYARGAGYGGAAS
YGTGYYNGNYGPDRTIYNPDSRTRGSYGGFMRGGGSSSNDGIFGATDFGSYAKQIDTTSY
DSSESYDRVESYGGIPSGTPRGIPYNGYHNNANIRWPDSNSQGNARKNGSSLEDVEPCTIL
CIFRQMKMTNGDSYLEQQSVAAVLMRRARDPQLKDFIGRTVQMCFERFGLANKGRCESA
KLFALCMEEAGKMNCEDWDVNKRFALKNPKPGPVLTMPQPLPPPPPRG
>AsutOBP23

MFAITAALSLLLLNVAIAHPGHFDEDPECRPPHHHRLEEKDCCKIPNLFSNSRDEMHELVH
KCFEEAGIKKHGHHDHHGPPPPPGLGLPPPPPPPPKNDSKFDCVEQCFLKNLELINEEGEV
KVDELKALIAEKFTGDWASVGSSTIEKCLEKSKTEENDSSKCKAGSKRILICLARESFLSCP
ASEWTESDVCTAAKDRLEKCPHAPPPMNN
>AsutOBP31
MGRLTKTQNADLLIHPAGKIRVGVLPELFVGGIQDVIPCHDEARLKKPSGRHGGGGSPPSA
EDMAAHLRAHECADECVLKSQNLLTTDGELNKDAIKAQVVKSFTGDWAKLASDTADKC
LASAKGEVTATATCKSGAGQFIYCFRRNLFLQCPSSSWTETTDCAAAKARITKCPNAKIPM
GHHRH
>AsutOBP32
MDVMILPVFLIVFIAATASPAVLSAECPQRFPKEMRPLSSCCTVEMNPNSSYNYTEGDPIIN
KCFGDFNSSATRPTGPPSGYDCELECLMIEFGYMGKDKTINKDKIVKSIEDEYSADFQEAG
HKAIEICMGRKYHTSCPSGIDGMIECFAVQMMLNCPAKHWSGGEDCKETKQLIDKCGEVL
SIFADYDTD
>MsanOBP9
MIIKKTLLVSVFVIFGCLFSFNKAADDADAADKELISKLFTVVFKCFKDADWGTCGEMLT
TKYDIAQAKYKQCTCHLACAGEELGLINTSGQPEPAKFLEYVNRINNPGIKSQLQLIYDKC
QNVKGSEKCDLAEQFSICAFKESPAVSNNNYTSDGIII*
>MsanOBP2
MKVSAATAVLVALVATVQSSDPCNISTCYKSGTTKPPIAVTPTHLPVQSSTPTNHPQTTYAK
DHVHGSTTTKSGANATVTTASGASVNGTERPAVVKSSVGVTGNSTTPKPTMTEGHVALK
QKLNTIAVKCKDELHAPQEIMALVSNTVVPQNEQQRCYLECVYKNLNLIKNNKFSVEDG
KAMAKIRFANQPEEHKKAVTIIETCEKEAVIDPKTTEKCAAGRVIRNCFVKNGEKINFFPKA
*
>MsanOBP4
MRGNYSLMVFLLFTIGLQDIYCQKQELSGKCRAPDKAPLNLEIIINICQEEIKSALLQEALDI
LNDGNLEQNTPSYSSRSKREADEDLTNEERRVAGCLLQCVYKKVKAVDETGFPVVDGLM
KLYNEGVQDRNYYMATLSAVRHCISIAQQLKQQQPSKSFDDGQTCDLAYEMFECVSEKIE
ENCGVENKSNNLSQRQV*
>MsanOBP10b
MEHLRSSNVVFAIVMALLVVQSSTRPQPDEIEEIKKTLYNACSGKFPITEEIKNDFKNSIISD
DPNFKCFLRCCLDEMSLIDEDGIIDGDSLKEMAIDSIKPIIEQVNSNCLKNVKQDGCQAAFE
FISCGIKLNPLTMQLLPL*

File S2. The amino acid sequences of 77 chemosensory proteins (CSPs) from 10 Hemiptera species.

>AgosCSP1
MNILTIFCYVTVMCDTQVKPAVSAQRLQSVNQNVTPTNDGRKTIRETSSYPTRYDYIDIEA
VMNNERIIKILFNCVMSRGPCTREGLELKRIVPDAIQTECAKCNERQRKQAGKVLAHLLQ
YKPEYWKMLVQKFDPNNVYLRKYMADNDDDEKLSLQKLSNDTTKKKRNI*
>AgosCSP2
MAHLNLFVVLIASLIYFTSAAEEKYTTKFDNFDVDKVLNNNRILTSYIKCLLDEGNCTNEG
RELKRVLPDALKTDCSKCTDVQKDRSEKVIKFLIKNRSTDFDRLTAKYDPTGEYKKNLEK
FEKERASAKPLKA*
>AgosCSP4
MDSRIAVVCVVLAAFAVDQTVGAPQKDAVAASGPAYTTKYDHIDVDQVLASKRLVNSYV
QCLLDKKPCTPEGAELRKILPDALKTQCAKCNATQKNAALKVVDRLQKDYDAEWKQLL
DKWDPKREHFQKFQQFLAEEKKKGFTKF*
>AgosCSP5
MHCKVLIALCCVAVYAVQASPAGTATAAAVSADDEIKDFPAYMKRFDKLNVEQVLNNDRV
LASHLKCFLNEGPCVQQSRDLKRVIPVIANNGCNGCTERQMTTIKKSLNFLRTKKPTEWA
RLVKIYDPSGTKLNKFLDA*
>AgosCSP6
MIKLILAIAFCVSITMTVVQTAPAKYTTKYDNVNIDEILNNDRLVASYFKCLMETGKCTPE
GEEIKRWLPEAIENKCEDCSEKQKLGSEKIIKFLFEKKNDMWKQLEAKYDPQGTYRQRYA
EEAKKLNINV*
>AgosCSP7
MSRSSSSVTMKVFVIAICVCAALARPEDVKVENKPAVIKSETLAAPLPTNIVKRATDTIQLD
SSLPNVSEDVLDKALSDRRFVQRQLKCATGEGPCDPIGRKIKAHAPLVLRGMCVKCSQSEI
KQIQRVMSHIQKNYPKEYTKMLKQYQSGF*
>AgosCSP8
MNNIIMNNSRGRYGIFSLLAVTIAAIMLVHQPATVRCADGGIITPQQQQQQTMMFTAPTGY
YVSTYDHIDVGRLLRNNKVVSGYVKCFVNEGPCTPDGKLVKAYLLPEIIRTVCGKCTPRQ
KDMARMVLKHIYTYRQADFEKIMQIYDTDGKRNEILAFMNH*
>AgosCSP9
MSAFCLNSFILMTMITVIVTHATFTRSTKFDDRTGIDIHLVKRDTDDVNDDENSVESDEGFF
YRFTHFFQDSSDKEDDDDDEKKPDFITTFDIFKLLDEEYAMQQFYCVINEDPCDEVGMRL
KATIPEEINRNCERCTSTERNNIRRILNYVKKHYPQFWKRVEPIYKKKI*
>AgosCSP10
MINTRPRKLVRCIRGVSISVAKGDDAVNAENKDDDSHLVNREEIQRYMSMMEKINIDQML
NNTRLMSNNVKCFLNEGPCTAHLREMKKMVPMLVKDSCSSCTKEQKIMMKKAMDAVK
ARRPNDYEKLSKFFDPEGKYEKKFLENLNESK*
>MperCSP1
MNLLAVFCYITMMCDSQLFKRLEQPAAISQVKRIEQPAMIANRIGQPTVAPRFGQPTIAPRF
GLPTIAPQVGQAAITPQVGQAAIASRFGLPTVAPQVGQAAITPQVGQAAIASRFGLPTVAP
QVGQAATTPQVGQAAIASRIGQNFQNANNSVSPTTDGRKTTRETSSYPTRYDFIDIEAVMN
NERIIKILFNCVMNQGPCTREGLELKRIVPDAIQTECAKCNERQRKQAGKVLAHLLQYKP
EYWNMLVKKFDPNNVYLKKYMADNDDDEKVSLQKLTNDTTK*

>MperCSP2
MAHLNLFVVLVASLVCFTLAEEKYTTKFDNFDVDKVLNNNRILTSYIKCLLDEGNCTNEG
RELRKVLPDALKTDCSKCTEVQKDRSEKVIKFLIKNRSTDFDRLTAKYDPSGEYKKKIEKF
DSEKAAAAKH*
>MperCSP4
MDSRIAVVCVVLAVFAVDQTVGAPQKDAVAASGPAYTTKYDHIDIDQVLGSKRLVNSYVQ
CLLDKKPCTPEGAELRKILPDALKTQCVKCNATQKNAALKVVDRLQRDYDKEWKQLLD
KWDPKREYFQKFQQFLAEEKKKGVVKF*
>MperCSP5
MNCKVLIALCCVAVYAAHASPAGAATAAAASADEEIKDFPAYMKRFDKLNVEQVLNNDR
VLASHLKCFLNEGPCVQQSRDLKRVIPVIANNGCNGCTERQMTTIKKSLNFLRTKKPVEW
ARLVKIYDPSGTKLNKFLDA*
>MperCSP6
MNTLLLAVALCIAITMTVVQTAPAKYTTKYDNVNIDDILNNDRLVASYFKCLMETGKCTP
EGEEIKRWLPEAIENKCENCSEKQKIGSEKIIKFLIEKKNDMWKQLEQKYDPQGLYKQRYS
EEAKKLNLDV*
>MperCSP7
MDRSSSSVTMKVFVIAVCVCAALARPEDSKVENKPAAVKSETLAAPLPTTIVKRATPQVVS
TQQGASLPNVSEDVLDKALSDRRFVLRQLKCATGEGPCDPIGRKIKAHAPLVLRGMCVKC
SQSEIKQIQRVMSHIQKNYPKEYTMMLKQYQSGF*
>MperCSP8
MTNNNMNSPRCRPEIFSLLAVAAIATVLVHQPSTVHCADAGVYPPQQQQQEATMFTAPSG
YYVSTYDHMDVGRLLRNNKVVAGFVKCFTNEGPCTPEGRLAKAYLLPEIIRTVCGKCTPR
QKDMARLVIRHIYTYRRGDFDKIMQIYDTDGKKNEIIDFMNQK*
>MperCSP9
MTSFCLNSVILMTITTVIVAHAASTGMTAFNNRSGSDIHMAQRDYNENKADKAEGFFFTIT
NFFSRRKHDDDKPDFITTFDIIRLLDEKYAMKQFYCVINKEPCDATGLRLKATIPEEINNDC
ERCTATETSNIRRILNYVKKHYPEFWDRVEPIYRNNMTA*
>MperCSP10
MVSKLFVSVFVLMSVVGVSYSVTEGDDDAAKVADKDLHPVNQEELKKFLSMMEKVDID
QILNNNRLMSNNVKCFLNEGPCTGQLREMKKMVPMLVKDSCSSCNKEQKNMMKKAMD
AMKARRPNEYEQISKFFDPEGKYEKKFLENLNESK*
>ApisCSP1
MNLLAIFCYITMMCDSQFRRLEQMTAMPQVKQPATIATRIGQATIAPRFGQPTIAPRFGQAT
VAPQVGQAAVTPQIGQAAIGSRIGQSFQSVNGSVTPTTDGRKTTRETASYPTRYDFIDIEAV
MNNDRIIKILFNCVMNQGPCTREGLELKRIVPDAIQTECAKCNERQRKQAGKVLAHLLQY
KPEYWNMLVKKFDPNNIYLRKYMADNDDDEKLSLQKLTNNTTK*
>ApisCSP2
MAHLNLFVVLVASLVCFTLAEEKYTTKFDNFDVEKVLNNDRILTSYIKCLLDQGNCTNEG
RELKRVLPDALKTDCSKCTDVQKDRSERVIKFLIKNRSAEFDKLTAKYDPSGEYKKKIEKF
DAERAAAAKH*
>ApisCSP3

MVHLNLFVVLVASLVCFTLAEEKYTTKFDNFDVEKVLNNDRILTSYIECLLDQGNCTNEG
RELKRVLPDALKTDCSKCTDVQKDRSERVIKFLIKNRSAEFDKLTAKYDPSGEYKKKLEKF
SA*
>ApisCSP4
MDSRIALVCVVLAVFAVDQTVGAPQKDAASGPVYTTKYDNIDIDQILASKRLVNNYVQCL
LDKKPCTPEGAELRKILPDALKTQCSKCNPGQKNAALKVVDRLQKDYDKEWKLLLDKW
DPKREQFQKFQQFLVEEKKKGVVKF*
>ApisCSP5
MNCKILIALCCVAVYAAQANPAGVATATAADEEIKDLPAYMKRFEKLNVEQVLNNDRVLA
SHLKCFLNEGPCVQQSRDLKRVIPVIANNSCNGCTERQITTIKKSLNFLRTKKPVEWARLV
KIYDPSGVKLNKFLDA*
>ApisCSP6
MNKLFLAVAFCIVTMMTVVQTAPAKYTTKYDNVNIDDILNNDRLVNSYFKCLMETGKCT
PEGEEIKRWLPEAIENKCEDCSEKQKLGSEKIIKFLIEKKNDMWKQLEEKYDSKGLYRQRY
SEDAKKLDIHI*
>ApisCSP7
MARSSSSVTMKVFVIAVCVCAALARPEEAKMENKPAVVKSETLAAPLPTTIVKRATPYVV
STQQDSSLPNVSEDVLDKALSDRRFVQRQLKCATGEGPCDPIGRKIKAHAPLVMRGMCV
KCSQSEIKQIQRVMSHIQKNYPKEYTKMLKQYQSGF*
>ApisCSP8
MTNNNMNCPRSRPEIFSLLTVTAIAAVLVHQPTTVYCADGGTYPQQQLQQQQQQQQQQ
QQQQFTAPSGYYVSTYDHIDVGRLLRNQKVVSGYVKCFVNEGPCTPDGKLVKAYLLPEII
RTVCGKCTPRQKEMARMVLRHIYTYRRADFDKIMQIYDTDGKKNEIINFMNQK*
>ApisCSP9
MSSFCLNSVILMTVITVVVARVAFAESTTSNDRPGSDIRLVKKDVDYNEDDADDREEGFFF
RISHFFGFTSYDDDKPDFITTFDLIRLLDEKYAMKQFYCVINEEPCDAVGLRLKATIPEEINR
DCERCTATETSNIRRILNYVKKHYPKFWERVEPIYRNNTTA*
>ApisCSP10
MVSKRFISVFMFMAVVGVSFSVPEDDDATKVVNKEVDHHSVIQEEIKKFLSMMEKINIDQI
LNNDRLMSNNVKCFLNEGSCTAQLREMKKMLPVLIKDSCSSCTKEQRNMIKKAMDAIKA
RRPNEYERVTKFFDPEKKYEKKLSEKLNES*
>SaveCSP2
FVVLVASLVCFTLAEEKYSTKYENFDVDKVLNDDSLLTSYINCLLDEENCTEEGQALKRVL
PDALKTNCGKCTDTQKMKIEKILKFLMKNRSTDFDRLTAKYDPSGEYKKKLEKFSA
>SaveCSP4
MDSRIAVVCVVLAVFAVDQTVGAPQKDALAAGSPTTYTNKYDHIDIDQVLASKRLVNSY
VQCLLDKKPCTPEGAELRKILPDALKTQCAKCSATQKNAALKVVDRLQKDYDKEWKQL
LDKWDPKREQFQKFQQFLTEEKKKGVVKF*
>SaveCSP5
MNCKVLIALCCVAVYAAQANPAGAATATAADDEIKDFPAYMKRFDKLNVEQVLNNDRVL
ASHLKCFLNEGPCVQQSRDLKRVIPVIANNGCNGCTERQMTTIKKSLNFLRTKKPVEWAR
LVKIYDPSGTKLNKFLDA*
>SaveCSP7

MARSSSTSVTMKVFVMAVCVCAALARPEEAKMENKPTAVKSETLAAPLPTTIVKRATPQV
VSIQKDASLPNVSEDVLDKALSDRRFVQRQLKCATGEGPCDPIGRKIKAHAPLVLRGMCV
KCSQSEIKQIQRVMSHIQKNYPKEYTKMLKQYQSGF*
>SaveCSP1
MNLLAIFCYITMMCDSQFRRLEQPTAIPQVKRIEQPATIATRIGQATIAPRFGQPTVAPRFGQ
PTIAPRFGQATAAPQTGEAAIGPRIGQTFQNVNDSVSPTTDGRKTTRETSSYPTRYDFIDIEA
VMNNDRIIKILFNCVMNQGPCTREGLELKRIVPDAIQTECAKCNERQRKQAGKVLAHLLQ
YKPEYWNMLVKKFDPNNIYLRKYMADNDDDEKLSLQKLSNNTTK*
>AglyCSP1
MNILTIFCYVTVMCDTQVKPAVSAQRLQSVNQNVTPTNDGRKTIRETSSYPTRYDYIDIEA
VMNNERIIKILFNCVMSRGPCTREGLELKRIVPDAIQTECAKCNERQRKQAGKVLAHLLQ
YKPEYWKMLVQKFDPNNVYLRKYMADNDDDEKLSLQKLSNDTTKKKRNI*
>AglyCSP2
MGINIYKLKRIKMAHLNLFVVLIASLIYFTSAAEEKYTTKFDNFDVDKVLNNNRILTSYIKC
LLDEGNCTNEGRELKRVLPDALKTDCSKCTDVQKDRSEKVIKFLIKNRSTDFDRLTAKYD
PTGEYKKNLEKFETERATAKPLKA*
>AglyCSP4
MDSRIAVVCVVLAAFAVDQTVGAPQKDAVAASGPAYTTKYDHIDVDQVLASKRLVNSYV
QCLLDKKPCTPEGAELRKILPDALKTQCAKCNTTQKNAALKVVDRLQKDYDAEWKQLL
DKWDPKREHFQKFQQFLAEEKKKGFTKF*
>AglyCSP5
MHCKVLIALCCVAVYAVQASPAGTATAAAVSADDEIKDFPAYMKRFDKLNVEQVLNNDRV
LASHLKCFLNEGPCVQQSRDLKRVIPVIANNGCNGCTERQMTTIKKSLNFLRTKKPTEWA
RLVKIYDPSGTKLNKFLDA*
>AglyCSP6
MIKLILAIAFCVTITMTVVQTAPAKYTTKYDNVNIDEILNNDRLVASYFKCLMETGKCTPE
GEEIKRWLPEAVENKCEDCSEKQKLGSEKIIKFLFEKKNDMWKQLEAKYDPQGIYRQRYA
EEAKKLNINV*
>AglyCSP7
MYMGNPSPSIDRIWSHYCHHLNTRSMSRSSSSVTMKVFVIAICVCAALARPEDVKVENKP
AVIKSETLAVPLPTNIVKRATDTIQLDSSLPNVSEDVLDKALSDRRFVQRQLKCATGEGPCD
PIGRKIKDILGADPSSRTASVERNVRQVFTVGNQTDSTCHVPYSEELSQGVHQDAETVPER
ILITMRRPCTIFLFMTSGFWHNLY*
>AglyCSP8
MNNSRGRYEIFSLLAVTIAAIMLVHQPATVRCADDGIITPQQQQQQTMMFTAPTGYYVSTY
DHIDVGRLLRNNKVVSGYVKCFVNEGPCTPDGKLVKAYLLPEIIRTVCGKCTPRQKDMAR
MVLKHIYTYRQADFEKIMQIYDTDGKRNEILAFMNH*
>AglyCSP9
MSAFCLNSFILMTMITVIVTHATFIRSIKFDDRTGIDIHLVKRDTDDVKDDENSVESDEGFF
YKITHFFQHHDKEDDDDDEEKPDFITTFDILKLLDEEYAMEQFYCVINEDPCDEVGMRLK
ATIPEEINRNCERCTSTERNNIRRILNYVKKHYPQFWKRVEPIYKKKI*
>AglyCSP10

MNSKIFISVFMFITIVSVSISVAERDDAVKAENKDDDSHPINREEIQRYMSMMEKINIDQML
NNTRLMSNNVKCFLNEGPCTAHLREMKKMVPMLVKDSCSSCTKEQKIMMKKAMDAVK
ARRPNDYEKLSKFFDPEGKYEKKFLENLNESK*
>LeryCSP
MDSRIAVVCVVLTVFAVDQTVGAPQKDTAVVNGPAYTTKYDNIDIDQVLASKRLVNSYVQ
CLLDKKPCTPEGAELRKILPDALKTQCTKCNATQKNAALKVVDRLQRDYDKEWKQLLD
KWDPKREYFQKFQQYLAEEKKKGVVKF
>AsutCSP1
MLPFYVFSLCAVFVACQETYTSKYDNVNVEDALKNDRLYKAYFNCLADRGPCTREGNML
KEALPDGLRNNCSLCTDPQRRGTHQVIRFLFKYRPEDMKLLEEIYDPEGIYKTKYAEERK
KLME
>AsutCSP2
MGHFPPVFSLSPVLLVASLHTMNTSTLLKIAFLLGCVAACLAAETRSSVSDEALEAALKDK
RYLTRQLKCALGEGACDPVGRRLKTYAPLVLRGACPKCTPSEVRQIQQVLSHIQRHYPKE
WAKILKQYAGQ
>AsutCSP3
MKFVAALLVASVAVLAVEAANQYTTKYDNIDLDDILKNQRLYKKYFECLTGKGKCTPDGK
ELKEHLPDALKTGCSKCSEKQRAGSEKVIKHLLKNKPQDYAVLEKIYDPSGIYKKKYEAE
AKKLGINV
>AsutCSP4
MRIILSAFLVAMACSLATCEMTEEEFYTKVFEEVDPDFILDNERILTSYLKCFYSEIECNAHA
EVVKKSIPDVLATVCGRCSDKQKSIFKYSLNKFIPAHPKDWEKILSIYDPSGEAWPKVKAFI
ES
>AsutCSP5
MDYKFFVVMQIGVISSVCAAGTYTDKYDNVNLDEVLNNERLYRNYFNCLQGKGKCTLD
GAILKEVIPSALKTDCALCSVRQKKGAEKVLIFLITKKPDDFKILEDKFDPEGVYRKKYEA
QRKLVEEGKPIH
>AsutCSP6
MVCKLFAVVLMGILAGVWAADKYTDKYDNIDIDEILTNERLYKKYFDCIQGIGKCTPDGIE
LKEKIPEALKTECAKCNEKQKAGVEKVMRYLITKKPEDFKILEDKFDPEGVYRKKYEAQR
KLVEEGKPVEY
>AsutCSP7
MVSKLSMVLLIGALADVWASELYTDKYDSIDIDEILNNDRMYKNYFNCVMGNGKCTPDG
TELKAKIPEALQTECAKCSDKQKKGVEKVLRFLIKEKKDDYKLLEEKFDPEGVYRKKYE
AQKKLVEEGKPIEY
>AsutCSP8
MDYKLSVMLVMGVLACAWAADMYTDQYDNIDIEEILTNERLYKKYFDCIIGNGKCTPDG
TELKETIPDALKTACAKCNDKQKAGVEKVLRHLLTKKAEDYKILEAKFDPEGVYRKKYE
AQKKLAEEGKPIVL
>AlucCSP1
MLKVLVLLAAVVCCVSAAATYTSKYDNIDLDEILSNTRLYKKYFDCLANKGKCTPDGKEL
KESLPDALKTNCAKCTKKQQEGTDKVLRHVLKNKPNDYKVLESIYDPTGIYRKKYEIEAE
KRGIKLPGSH
>AlucCSP2

MVGKLSVVLLIGAVGMVLAADKYTDKYDNIDVDEILGNQRLYQKYFDCIQGKGKCTPDG
AELKKNIPEALQTDCAKCSEKQKAGVEKVLRHLINEKPEDYKVLEEQFDPEGVYRKKYE
HLKKKVEEGKPVEY
>AlucCSP3
MLKVLVLLAAVVCCVSAAATYTTKYDNIDLDEILSNQRLYKKYYDCLANKGKCTPDGKE
LKEALPDALKTNCSKCSKKQQEGTDKVLRYVLKNKPNDYKVLENIYDPSGNYRKRYEDE
ASKRGIKLPGSH
>AlucCSP4
MVSKLSIVLLIGALADVWASELYTDKYDNIDVDEILGNQRLYQKYFDCIQGKGKCTPDGA
ELKKNIPEALQTDCAKCSEKQKAGVEKVLRHLINEKPEDYKVLEEQFDPEGVYRKKYEH
LKKKVEEGKPIEY
>AlucCSP5
MVGKLSVVLLIGAVGMVLAAELYTDKYDNIDVDEILGNQRLYQKYFDCIQGKGKCTPDG
AELKKNIPEALQTDCAKCSEKQKAGVEKVLRHLINEKPEDYKVLEEQFDPEGVYRKKYE
HLKKKVEEGKPV
>AlucCSP6
MVSKLSIVLLIGALADVWASELYTDKYDNIDVDEILGNQRLYQKYFDCIQGKGKCTPDGA
ELKKNIPEALQTDCAKCSEKQKAGVEKVLRHLINEKPEDYKVLEEQFDPEGVYRKKYEH
LKKKVEEGKPIEY
>AlucCSP7
MVSKLSIVLLLGALADVWAAELYTDKYDNIDIDEILNNDRMYKNYFNCVMGNGKCTPDG
LELKAKIPEALQTECAKCSDKQKKGAEKVLRFIINQKKDDYKLLEEKFDPEGVYRKKYEA
QKKLAEEGKPIEY
>AlucCSP8
MLKVLVLLANAASTYTTKYDNIDLDEILSNQRLYKKYYDCLANKGKCTPDGKELKEALP
DALKTNCSKCSKKQQEGTDKVLRYVLKNKPNDYKVLENIYDPSGNYRKRYEDEASKRGI
KLPGSH
>AlinCSP1
MLKVLVLLAAVVCCVSAAATYTSKYDNIDLDEILSNTRLYKKYFDCLANKGKCTPDGKEL
KESLPDALKTNCAKCTKKQQEGTDKVFRHVLKNKPNDYKVLESIYDPPGIYRKKYEAEA
EKRGIKLPGSH
>AlinCSP2
MKVAVLVLLCVGAALSAEVYTSKYDNIDVDKILSNDRILTRYIKCLMEEGNCTNEGKELK
KTLPDALASGCTKCSEKQKAQTEKVLRHLSKNRPRDWALLKTKYDPKGEYSKKYEKEA
KALTA
>AlinCSP3
MISKLSMVLLIGAFADVWAAEQYTDKYDNIDIDEILNNDRMYKNYFHCVMGNGKCTPDG
LELKAKIPEALQTECAKCTDKQKKEVEKVLRFIINQKKDDYKLLEEKFDPEGVYRKKYEA
QKKLVEEGKPIEY
>AlinCSP4
MRIILSAFLVAMACSLATCEMTEEEFYTKVFEEVDPDFILDNERILTSYLKCFYNEIECNAH
AEVVKKSIPDVLATVCGRCSDKQKSIFKYSLNKFIPAHPKDWEKILSIYDPSGEAWPKVKA
FIES
>AlinCSP5

MGHLTIVLLAAAFEVLTGSRAYTTHYDYIDVDQVLNNTRLYTKYVECLLGQGKCTPEARE
LRDKLPEALQTNCARCSERQASESHRVIRFLIQNRQEDFKLLEAKYDPSGLYFKRFEEETK
RNVSLS
>AlinCSP6
MFYKLSVVVLMGILAGVWAADKYTDKYDNIDIDEILTNERLYKKYFDCIQGTGKCTPDGI
ELKEKIPEALKTECAKCNEKQKAGVEKVMRYLITKKPEDFKILEDKFDPEGVYRKKYEAQ
RKLVEEGKPVEY
>AlinCSP7
MNYKLSVILLIGVLASVWAASTYTDKYDNIDLDEILTNERLYKKYFDCIQGKGKCTPDGTE
LKEAIPDALKTECAKCNAKQKAGVEKVLRHLLTKKAEDYKILEDKFDPEGVYRKKYEAQ
KKLADEGKPIVL
>AlinCSP8
MDYKLSVMLLMGVLACAWAADKYTDKYDNIDIDEILNNERLYKKYFDCILGNGKCTPDG
TELKETIPDALKTACAKCNDKQKAGVEKVLRHLLTKKAEDYKILEAKFDPEGVYRKKYE
AQKKLAEEGKPIAL
>AlinCSP11
MKVFFSGLLLVCMASVSLCADEYTDKYDSVDLDEILNNQRLYQKYIDCVMGKGKCTPDG
ALLKEKIPEALQNECAKCSAKQKKGAEKVLRFLINEKADDYKALEEKYDPEGTFRSKYEE
QKKNLKEGKPLSV
>AlinCSP12
MMIIIVFGISALLVVVEGAPLQYSDTRYDDVELTTILSNDELYIKLFQCLIGRGKCTPDWEIL
KDALPGALLDNCSECSNKQKFGTKTLLAHLVHERPSDMRLLEGEFDPDGSYRKELEKEEK
ESNDINRKRSANLEEVEILDKIKRIIK
>AlinCSP13
MKFVAALLVASVAVLAVEAANQYTTKYDNIDLDDILKNQRLYKKYFECLTGNGKCTPDGK
ELKEHLPDALKTGCSKCSEKQRAGSEKVIKHLLKNKPQDYAVLEKIYDPSGIYKKKYEAE
AKKLGINV
>AlinCSP14
MNSAIVLCVVALAGMVLARPDDTYTTKYDNVDLDEILGNDRLLVPYIKCTLDEGKCAPD
AKELKEHIREALENGCAKCTDKQKEGTRRVIAHLIKHKNADWQKLKAKYDPEGKYTHK
YEKELEEVQH
>AlinCSP15
MKLIVAVALLCVVAESWAASTYTDKWDNINVDEILESQRLLKAYVDCLLDRGRCTPDGKA
LKETLPDALENECSKCTDKQKSGSDKVIRHLVNKRPEMWKELSAKYDPNNIYQDRYKDK
IEAVKGQ
>AlinCSP16
MLPFYVFSLCAVFVACQETYTSKYDNVNVEDALKNDRLYKAYFNCLADRGPCTREGNML
KEALPDGLRNNCSLCTDPQRRGTHQVIRFLFKYRPEDMKLLEEIYDPEGIYKTKYAEERK
KLME
>MsanCSP2
RYFRFDQIYFCQNIYSLKQTKMAHLNLFVVLVASLVCFTLAEEKYTTKFDNFDVDKVLNN
DRILTSYIKCLLDQGNCTNEGRELKRVLPDALKTDCSKCTGVQKDRSEKVIKFLIKNRAAD
FDHLTAKYDPSGEYKKKLEKFEAERIAAAKN*
>MsanCSP7

MARSSSSVTMKVFVIAVCVCAALARPEEAKMENKPTVVKSETLAAPLPTTIVKRATPQVV
STQQDSSLPNVSEDVLDKALSDRRFVQRQLKCATGEGPCDPIGRKIKAHAPLVLRGMCVK
CSQSEIKQIQRVMSHIQKNYPKEYTKMLKQYQSGF*
>MsanCSP4
MDSRIAVVCFVLAVFAVDQTVGAPQKDAVATGGSAYTNKYDHIDIDQVLASKRLVNSYVQ
CLLDKKPCTPEGAELRKILPDALKTQCAKCNATQKNAALKVVERLQRDYDKEWKQLLD
KWDPKREQFQKFQQFLTEEKKKGGVVKF*
>MsanCSP1
MNLLAIFCYITVMCDTQFRRLEQPTTTPQVKRIDQPATIATRIGQATIAPRFGQVTAAPQIEQ
AAIASRIGQGFQNVNNSVSPTTDGRKTTRQTSSYPTRYDFIDIEAVMNNERIIKILFNCVMN
QGPCTREGLELKWIVPDAIQTECAKCNERQRKQAGKVLAHLLQYKPEYWNMLVKKFDP
NNIYLRKYMVDNDDDEKLSLQKLTTNTTK*

File S3. The amino acid sequences of 85 odorant receptors (ORs) from 11 Hemiptera species.
>ApisOR2
MDVMQKPERFILTPFQKFCIRWSVFFDSSSDRLSRIETVLRTIQFSTIMITSGMTMTSVLIAD
NKKALESFTYFVICVFMLAIITFAIRTKRFNRAMLLMVVDEFPGYNRPMPDVLKRKMAAI
RTSYGDFTMKVIVSYLTLVLFEIPATAMVPLAAASLTDVKLGSQSTQMVVLWFPADTSQVG
MYAVSYVIQFLIVVTVKFIITGIMCSFSFFVSQMISEFQILSAYVEHAVEIVEYDQSADKTTE
QKLLDHVKNCVMLHDRLIYFKDQLNESYGYIILLELMFSTLYFCLSAFNMIFVGNRFVMV
KGLLTLSNYLAELFIFCMYGSMVEDAHMGLLRASYSVAWYAQPVRFRQSLTMVMSRTQT
PLQLTVGKVFIANLPLFLSVLKVSYSGVNALRAANAK
>ApisOR2a-like
MTTTPRVTELTAPASEDLTIVDNRLFKAICLHQILDPTKGGNRYYRLAFMVVMWVSLSVQI
IQLVGLYFAVNDLQRFAFTTTVIFNALLCLSKGYVLVVNADRLRASLEVARYEFTSCGARN
QRLVRRSRAVLSTILRTFAVLSWVTCFIWALTPLFAMDEYLQVTNADGTVSRYRVTIYNVW
LPVPATVYNETTVWSLVYAVEVIACFVNVFSWLLFDSYVVTMCFTFNAQFRTVSASCTTIG
HHSDSFRSPPPHAPEGTNDDNNTFNCYDELINRIKDNQSIIKIYDDFFEILQPAILFQIIGGSY
SVITLIFLTSLTYLMGFSIISIPVLKVFFGFLSVTFELFLYCYVFNHIETEKCNMNFGLYSSNW
TAMDLKFKKTLLFAMNTNSSHRRVMKVTPMSIINLEMFANVMNMSYSIVSVLLNSRVQK
>ApisOR4
MTSIGKKNQRKFYQTLMTLAFFLDTSQYRYISRFVKQFYIFDWMVLVSVAAAFTILEGNY
RMPFVMELIQYMIVGFYFTSIFVVFIIKKEAIMSNYNCIQTKFIQWSNKRALHSNAAYKRNI
KTVKSLSIPLAILSLSIALGPLISTINDIGKLPLDNRAHFVLFWPTIVDTNKLSMYGIIYTLQV
IFTIILYISVLSFNLGYMVFLNELITQFEMLLNGINDAFKYKMDKQFQTLFIDCIRHHQIIIKF
LDDLKSYFKWMILIEIIVVQVILAILIYNLTKVNASLGYKVKIAGSILFNLLPICFHCHVGEV
VLSLHTRLSNHIYNMPWYDMPNKNKQLIVIMLQRTQRDLTLSSALFSSERASRSLISKVIK
QVYTILNVLLKT
>ApisOR5
MQRIDTINMFLQMTGCTDSKAMLYLTYFEFLITFYYLIATYASIVHFEQSVTIQLFALLCMLI
ECVILLNITFRLYHKNHIREMHQYSRRLGIPDSYRSVINVITKYHLIASNIFVVFPVTYAIFCD
SVRVGDPFTFPFLDVLPMHTDNLAIYACKYLVYAISVYIAHVELCFINTTFIYYVGVLKHRL
ETIVQTIGEAFADNDEQKFKYAIIQHQKLLSYFNTMKIVFSKPILLSMSFNAIYFGLTTSFVI
QAIRGYINQAILSICIASSAAAVINITIYTFYGSELMDLHDKILHVLFDNAFFYVSKSFKSSILI
MMTRVTIPLKFTVGYIFTINLNLLLKILKMSYTVLNVLLSSETIKPHKLS
>ApisOR10
MAHIVDIFFQNMGCSHDHGYGMVFFNCCELAITLFFTVSTYPTIADPTQNLSIRLYGVLCL
LIEAHIFAFIAVRIYHQSQHRDMYQHLHGVEIPENYRRKIATVIKHHFIISNVFVAVSVLYTIS
LDWVRIGDPFTFPFIDVLPIKTTNVTVYVCKYIVYALPVYFAHLETCFLNVTFMFSVGIVKR
HFQILNDQVEEAIVNEDEQKLKIAIKHHQQVLKYFEDMKTVYEKPILMTIEFCGLYVGLTS
CFVIQVIQGFIHQIILGLCIVSSIACLMTIIIYCIYASNMYALHNGILNALFEHRSCYSRNKSFK
RIILIMMTRATIPLEIKAGSVFTINLNLLVKILKFAYTVFNVLLSSINRQFKETAI
>ApisOR17
MTTTPRVTELTAPASEDLTIVDNRLFKAICLHQILDPTKGGNRYYRLAFMVVMWVSLSVQI
IQLVGLYFAVNDLQRFAFTTTVIFNALLCLSKGYVLVVNADRLRASLEVARYEFTSCGARN
QRLVRRSRAVLSTILRTFAVLSWVTCFIWALTPLFAMDEYLQVTNADGTVSRYRVTIYNVW
LPVPATVYNETTVWSLVYAVEVIACFVNVFSWLLFDSYVVTMCFTFNAQFRTVSASTTIGH

HSDSFRSPPPHAPEGTSDDNNTFNCYDELINRIKDNQSIIKIYDDFFEILQPAILFQIIGGSYSV
ITLIFLTSLTYLMGFSIISIPVLKVFFGFLSVTFELFLYCYVFNHIETEKCNMNFGLYSSNWTA
MDLKFKKTLLFAMNTNSSHRRVMKVTPMSIINLEMFANVMNMSYSIVSVLLNSRVQK
>ApisOR20
MRSSSATVVDVMLFKAIGLYQLLCPADRGGYSVRSRRALMTALGLSFALHSFQVPYLYYA
LNDLQRFAYMAAVIIYGMMCSFKGYVLVTNADRLWLVLNAADYGYTGCGHRDPSRLRR
CRATLSALLRTFVALSYGTLIVWIVLPFFVDEYTGITNSDGTVTRYRTTIHNMQYPIPLAVY
NSRPVWALIYVTELYVCIVNVFIWSLFDCYLVTMCFVLNAQFHTMSAGYGTLGIRRTGSSP
PDTTFAGVRRIKFDEIESNHYSDLISHIQDNQNLIKMFDVFFEVVRPVVLVQIANGSYSVISL
IFLTALMYLMGVPVLSAAFLKFICGLISLTIELFIFCYGFNHIETAKSVLNFGIYSSNWTEMD
LTFKKTMLLTMKMNSSHKRAMKVSPNSAVGLEMFARVMNMSYSTVSVLLNSRS
>ApisOR23
MNLNDEQNYIVNLKLMKITGFYHLISSRAPKYFGFNVYKVTAAIEVMTGIFSIIMLFLSSYY
YLDNTNELMSHFMLVVAIFFSTLKIFWVSRNSETIWNNMDMTCINFLSYTGHKKEILKKA
RAKSISTTILFVILWSSVTVAWSISPFFVKDVYLNIKFKDETRRFRYNSLNYVYPISEEFYNE
HFLYFYVVEMLSVVFWGHGTVAYDTFVISICITIAFQLKTIAVSYISLNDKKGDIKNLKDND
LEAMFNLKLLIQDQQNMFKKIKEIYKIFEPVTFVQLAAQSMLIILQAYMIFINHYNGFSLLS
VPIIKLIVTVAPNIIHLFITCYLYTNINHQQDSMNFALYSSDWTAMSINYKKMLLFTMRMND
AEKLKLKISLRKIVNLEMFASVMHLTYSIISVLAKSYGNTNTK
>ApisOR25
MATGIKTVSKNEDNFMINMRLMKKTGFYQLLDSRSLKVFGHNVFKCMSVVQMSILSSVA
FIFVANIYYFSDDINTVMMYSMLITSDVLSILKLYYILQNSDTIWNCIQMTSIDDLSYKYHD
RRILEEGRSKSTSYSILIMFMWLNLIVSWSLGPLFVTNYFLIVEQNDEIYRYRFNIMNFAFPA
TDRFYNDNFMIYYGIEFITLVLWCHCTMNFDVLLLSMNITFKYQLKTISNSFSAFNFTRYN
DFKNNRTKNVKHHKESESMFDFKSLIYDQQRVIENMKNIYRVFRPVVLTQLASESLIIMLL
SCIIMLNYFNGISLLSALNLRIFAAISTFLFHIYVICYLFDDVNEQKDSMNLALYSSDWTTSD
LQHKILLLHAMRMNNAENLRLQVTRNKIVNFQMFTYVRMIFFSFYYSGYCGHYFY
>ApisOR31
MNPTFKHFFKGDCTNITKPSPMETCIDHTCTINLNILKQCGFYQIFDPNSKKIFGWNVYRIS
FIALTVITQCLIGFGNCGFLFELEDTTDNIDLFLIIFSNSYFCLTEWKVVILIINRKKFLELLDV
TDLIFLKSKQCRKNIKILCKHRIRALQLTNLYFKFCIFVIIEWIIFPIMINSFIAHKTENRRLEN
VVNRRYPVDVNTYNKYYILFYVFEIIIGVKTVYLVLMVDILLLSIGWAIVIQYEVLAEAFKN
IGYNENLQKDHDHDVDDYKYFKSILFDQQQLDSKVKLYFPIVKPIVLMHVAINSVLFIMLS
NSFLMVFLSTESFTYKIVNLFKIGTGILYICLQLFLYCHLFDNINLKRKSVNLGIYSCNWTK
MDLKFKKLLLLTMQINDANYITIKASTKTIVNLPIFANVLMTSYNIVSVMVKTMSKYRKT
>ApisOR37
MKWLQDHEVAINLALFKRYQFYQIFNPNGSKLLNYDTYKLTNVMFIVAVTTYNIFSAMCF
FTDTIDTIDSVDLLLMIFIYSIIIISLLKISVLLFNADQIWELFDLTRFDFLTSRQCRKNVGILC
KYRDRSITITNLYQNYSTMVFIIWMITPLVLNTFVVVGGPNQRYHNIFNMQYPVSANIYNQ
YYYLFYLMEIAMGIFVLNYSMIVDNFLISLCWVIIAQYEVITTAFEKIGNDCELTTLQNEKN
NNSFEAYEDLKSILMDQNKLYIKLKSFYRVVWIIVIFLIIIDSVLLIILTYSFVMICSSAESFSIF
NILKISTAFFVFVIQLYLYCYLFDVLNDKKESVNFGLYCCDWTKMDLRFKKLLLLATKFNN
ANTLKIKSTPNKIVNLQLFSSVMTTAFNIVTVMLKTMNGKN
>ApisOR38

MGIDNMSSLKSNEVAINLKLFKVFRFYHIFDPNSGKLCKFNVYHLAWYIINCVIGCILIYGL
LGYFTEMEDVIDSIFHIQIMFCYLLYSLSLLKIITFLYKANNIWDLLRVTRINFLTSTQCQAHI
GILHKHRNKSIKITNLISGFAIVTTLEWILFPLVLRLLSKTDASHSNKRFENIFNFRFPVTVCE
YNNYYFIFYIMESFIAIFMLYAYVVTDVFFISVCYVIIAQYEIIKRAYEIVNCEQTSENNNEN
KNHNNIIVNDCCDDLISIVMDQQNHYAKLRLFYSTYKLIIVSTVVINSGSIIILTYASVVIFTS
PETIPILSIVKLISAFTYMFFVLFFLCYLMECINNKIESVQLGMYSCNWTAMNIKSKKLLLFS
MRMHNANKLMIKTTPNNIINLQLFNSVMMTSYNIVSAMVNTRSK
>ApisOR39
MFSCDFINRTVNMNSENLFNGGSVAFNLSTYKQLGYYQLLDPKGPHIYGYHLYRTILKIFL
LIVQFITIFGVMGFFIEMEDTDPGKSNSFELIIILTNCSLSSLKIYTLISNSKIIWDLFDLTRIDF
LRCSRHSKLITKNFVKRCKKSTTITKWIARSFLVGLILWLMGPFIANEEHTEPNTVHRHKNI
INIKFPVTMKTYNNYYFVFYLMEVAVGFCIVYGSVLIDAYLMSFCWIISAQYQSVTKAFAT
FGYNKQGSPKDIYKDFKSIIIDHQNIYLKMKSFYAVVRPITLIHVFAYSCSLIMYAYVIVTIFN
SKELFIIAEIMKIVMTVSNVTMEVFIFCYLFELIDNKKEDVNFGLYSCNWTGMDIKFKQLL
LMSMKMNNANRFKLKASPDVTINRPFFANVIHTCFKIVSVLIQTQSIDLLN
>ApisOR42
MPNSSEECVMSSSMAKCTGLHYIIDPEGPTVGGHNVFHVTVMVMIGFTVVCLSMCPFGL
YYWANDVTQCIFLLIYIVNFSFGCFKAFTLVRHSDDICRCLDVTRFDFSSGAIMSDPDSARF
FRKCRDASSTFTGWFAASSHFVLLVWTLLPFVVVGKGVEINNRDGSTSYYHFNPYNMYF
LVSSETYNRLHLVFHLVEWAFGLCFVLIMVAFDTFMVTLCVAITCQMRGIGNAYSKLGHD
RCATASNVCSDGGIESNKSNNEYLRDLKLIIKDHQAVLGKMNDFYKIVGPVILPQLIVASFT
IIFVSFIITRNYFNGMLLTSTQSLKMCCFPIFFYQVYYTCHAFGNLSHRKNVMNFALYSSDW
TQMEIKFKKLLLLAMQMHDANKLDMKLTDKLVINLELFTRVINMCYSIFSVLVNSQLKIA
DKQ
>ApisOR43
MDSKQEKQYIFNMKLARIMGLYQILFPNSTSFFGYNIYHVVTVFFVSFTFAISMLFPIGLLY
LRNDIIAIMYYMGCISNFLLSCFKMVNILYHSKDIWKCIDVTSFNYILYKHYDRNVFKNWQ
TRSIRITYIYIVIALFAFFCWIFSPCIMNKSVIAIRNIDGSYSKYRMNIFNLYLIASNETYNKNF
YIFYVIEIIISICYVVYFTIVFDVLMLLVCFAISYQLETISNTIKSLGHEIYTRDNIRSGNSIKLKE
KHGILYNDLITIMTDHQNVLKKLNDFYNIFRSITLTQIFIASSSHVFIWFIAAMSIDEGDNAD
SILSFKLFIVLPLINFQLFMTCSLFGTINEKKDSIIFALYSSNWTNMDLKSKKMILFNLTINNA
SQLKMKFTNTKIVNLEMFSHTMRFCYSIFSMLINYNKNKMK
>ApisORCO X1
MLPMHSFSPSYTHMGYKKDGLIKDLWPNIRLIQLSGLFISEYYDDYSGLAVLFRKIYSWIT
AIIIYSQFIFIVIFMVTKSNDSDQLAAGVVTTLFFTHSMIKFVYFSTGTKSFYRTLSCWNNTS
PHPLFAESHSRFHAKSLSRMRQLLIIVSIVTIFTTISWTTITFFGESVWKVPDPETFNQTMYV
PVPRLMLHSWYPWDSSHGLGYIVAFVLQFYWIFITLSHSNLMELLFSSFLVHACEQLQHLK
EILNPLIELSATLDSSVHNPAEIFRANSAKNQSINGIDHDYNGSYVNEITEYGTKGENEPNR
KGPNNLTSNQEVLVRSAIKYWVERHKHVVKYVSLITECYGSALLFHMLVSTVILTILAYQA
TKINGVNVFAFSTIGYLMYSFAQIFMFCIHGNELIEESSSVMEAAYGCHWYDGSEEAKTFV
QIVCQQCQKPLIVSGAKFFNVSLDLFASVLGAVVTYFMVLVQLK
>MperOR22b-like
MEDFRDEEVVINLKLLKQYRFYHMLKFNETKILNCNVYRLILFLYGSIMTCMVVYGSIVL
FVEMDDIISEADLFIVIFLTINFFFCVWRICTVLSKSNTICDLINVSRFNYLTSKHCCKHLNVL

YDYRERTIKITNYFFVFSMIVLMQWIIFPILAITFKKSDFENIRSENVMNFRFPVSTHTYNQY
FFIFYIMEVAIVTFPIYLIIVMDTLVLSFCCVIIAQQEVLSLAFRNIGHEENSQLEYYEDFKSV
LGDQIQLNLKIKSYYSLMRNIILVQVAMSSTFFIMVAYVLIVVCFSKDSNQILTIIKLGSSVIFI
GSEIFLYCYLFGSMNLKRESVNFSLYSCDWTKMDNKFKKLLLLTMRMNNANNLMIKASP
KKVVDLQMFANVISIAYNVISVMLKSMDSNN
>MperOR46a-like
MFTDTSFDVTHLEKRFDALYDDLKIIITDHQTVIRKLKEYCTIFRPVALFQIFITSSSHIVIWF
VAAMSFGENDLGDSTTTFKLFTVHPLISFQLFMTCWLYGSINEKKDSIIFALYSSNWTAVDI
KCKKLILLAMKINNANHLKLKFTNTKIVNLEMFTQTMRFCYTIFSMLVKYNYNKTTN
>MperOR43b-like
MPHIDTINMFLQMTGCTDNKKMLYLTYFEFLITFYYLIAAYISILHFEESVTIQLFTLLCMLI
ECVILLNIVFRLYHQNHIREMLQYSRRSGIPDSYQSIINLITNYHLIASNMFVIFPATYTILHD
SVRVGDPFTFPFLDVLPIQTGNLAIYACKYMVYAISVYIAHIELCFINTTFIYYVGVLKQRLD
TIVQTIQEAVVDDDEQKFKYAIIKHQKLLTYFNTMKIVFAKPILLSMSFNAIYFGLTTSFVIQ
AIRGYINQAILSICIASSAAAVINITIYTFYGSVLLDLQDEILHVLFDNAYFYVNKSFKSSILI
MMTRVTIPLNFTVGYIFTINLNLLLKIVKMSYTVLNVLLSSETIKPHKLS
>MperOR4-like
MSSSRPFVSVIDVMQKSERFILTPFQKFCIRWSVLFDSSSDRLSRTETVLRSVQFSTIMITSG
MTMTSVLIADNKKALESFTYFVICVFMLAIITLAIRTKRFNRTMLLMVEDEFPSYNRPMPD
ALKRKITDIRTSYGDFTMKVIVSYLTLVLFEIPATGMVPLAAATLTEVKLGSQSTQMVVLW
FPADTSQVGMYALSYVIQFLIVVTVKFIITGIMCSFSFFVNQMISEFQILSAYIEHAVEIVEFD
QSVDKTTEQKLLEHVKSCTMLHDRLIHFKDQLNESYGYIILLELMFSTLYFCLSAFNMIFV
GNRFVMVKGLLTLSNYLAELFIFCMYGSMVEDAHMGLLRASYSAQWYAQPVRFRQSLM
MVMSRTQTPLQLTVGKVFIANLPLFLSVLKVSYSGVNALRAANAK
>MperOR67a-like
MVISYQYKLLASAFEVLEYRMDNKDDSILSDEKLLETFISIVSDSQIIHKKLKMLYDIIRPIG
LIQLMADALGMICMPYLIVVYFVKYGSLFNPETLKFVFTLGIAGVQSYMYCSLFQRVNDR
RDGVNFGLYCCDWPGMNIHMKKMILFTMQMNSSNKLNMNITTHKAINLPLLSTIIRLSYR
ISSVLINSNIN
>MperOR22c-like
MENPERVDYDSNGVTGNPAEKAEGGEMDNPPKNDTEGGTILDVELFKIIGVYQLLRPDEF
GLNARLCRTTAIVVVCLTLGLQSMQVCRLYLARHDLQMFANVGVMIINGLMCLLKGYMV
AANADRMSATLNAARYAFTGCGNRDQSKLRLCRARLSTILRTFVRLSFGTLIVWVVMPW
FMASEYDDTPSIWATVYVIESIILTVNVFCWTSFDCYLVTMCFVLEALFCTMSTGYETLGR
HRAAAKSSAGQQLAIAGTISDVSISDVNYDDLTSHILDNQNIIEQYDEFFDVVRPMVLVQIA
NGMYSIITLIFLTLLTYLSGYSIVSAPFLKFVCGLASLTIELYIYCYGFNHIEDGKSTVNFGLY
SSNWTEMDLKFKNTLLMAMIMNSAHKRVMKVSPNSIVNLEMFTGVMNMSYSIVSVLLN
>MperOR33b-like
MEHQAMMEKYEDFLILFRPMILLQIFISSFSVIMLLFTFMMGFSNVEIFESSTFVQTRLFCTI
LPILIQIFTICYFYGNIHDQKDSILFALYSSNWTEMDMKCKQLILLTMKLNNANHKQLKFTR
TKIVNLEMFFKTMGDCYTVISVLVNYIQQNVDQI
>MperOR2a-like
MTTTGATTFAPASEDLTIVDNKLFKAICLHQILDPTKGRNRYYRSALLAVMWLSLSMQITQ
LVGLYFAVNDLQRFAFTTTTVTNAFQCLSKGYIIMTHADRLRASLETARYDFTSCGARDQR

IVRRSRNVLSTVLRTFIVLSWVTCFIWALTPLFGMDEYLQVTNADGTFTRYRVTIYNVWLP
VPATVYNATAVWALVYSAEVIVCFVNVFSWLLFDSYVVTMCFTFNAQFRTVSASCATIGH
GDCSGSPSPHATGTHNIIRDDNNILNCYDELINHIKDNQSIIKKCDDFFEIIKPAILFQIIGGSY
SVITLIFLTLLTYLMGFSIISIPVLKVFFGFLSVTFELFMYCYVFNHIETEKCKMNFGMYSCN
WTAMDLKFKKTLLFAMNNNSAHRRVMKVTPKSIINLEMFSNVMNMSYSIVSVLLNSRVQ
K
>MperOR64
MDVRNEKNYVFNIKLAKFIGLYRILDPGTVKCRGRNVYHIIMACILVYMFAISMILNLNGL
YYWTVNIPISIDYFWKSETTLYVIYKIWIVVRHSNDIWNCLSITRYCFTSSSNQNRHIILDRW
RERSVSLTTIYAIMYSMSTITYMVITLAFSEDVSPVKNHDGSVGYYRHNIMNFYLIVSDET
YNTHFYIFYIAEALYLIFLTISFLIFDILLVTLCFGMGCQLQLICCASESIGHKKLSDSNSPIDY
TDEYNKIPNEHDIIYDDLKTVIMDHQAVMEKYEKFILLFRRVMLLQIVVSSLSVITLWFIFI
MSFSNDDRFKASEVVIKKMFCSIPPLLFQIFMVCYLFGNLHNQQDSIIFALYSSNWTEMDM
KCKKLISLTMKLNNAYYKKLKFTRTKIVNLEMFFKTMGDCYSIISVLVNYIERKVE
>MperORCO X1
MQCQPLFTRRFVLRLQILNLRNLHNIIQRIGHMAMGYKKDGLIKDLWPNIRLIQLSGLFISE
YYDDYSGLAVLLRKIYSWITTIIIYSQFIFIVMFMVTKSNDSDQLAAGVVTTLFFTHSMIKF
MYFSTGTKSFYRTLSCWNNTSPHPLFTESHSRFHAKSLSRMRQLLIIVSIVTIFTTISWTTITF
FGESVWKVPNPETFNQTMYVPVPRLMLHSWYPWDASHGLGYIVAFVLQFYWIFITLSHS
NLMELLFSSFLVHACEQLQHLKEILNPLIELSATLDSSVHNPAEIFRATSAKNQAINGIDRDY
NGSYVNEITEYGTKGENESNRKGPNNLTSNQEVLVRSAIKYWVERHKHVVKYVSLITDCY
GSALLFHMLVSTVILTILAYQATKINGVNVFAFSTIGYLMYSFAQIFMFCIHGNELIEESSSV
MEAAYGCHWYDGSEEAKTFVQIVCQQCQKPLIVSGAKFFNVSLDLFASVLGAVVTYFMV
LVQLK
>RmaiOR2a-like
MSSFEVPDVAINIELYKLSCFYHVFNPNTTEIFGYHFYRFTGILLTVCIQLCVLFGLLGCCME
MEDTINYIERFIFIFVNSSNFLSVVKICVFIYKAKDTWDLFDVTRVNFLKSEQCCKFRNEILE
KVRNKSIRLTNFIFGFATMTFIIWIIYPLVVNFFLIATDQNNHQRYQNIFNMRYPVSINTYNK
YFFIFYAIEVIMGLFILYNSILIDTFLVSFCWVFIAQYKILSKAFENIELKDVDKLNKNCSINA
YNDLKLILGDQRRLNSKLKLYYSIIWYMVLTYVVFSSCSIITLTYSFIMVCISTTKSLPVLSII
KIIAPFIIVSFQLFLHCYFFGLINFKKESVTYGMYSCDWTSMDLKFKKLLLLSMRMNDVDT
LMIKATPTKIINLEFFVKVMITTYNIVSAMLNTFNSKI
>RmaiOR67a
MNTTDKRYAFDLTLFKIIGYYQMIDPNSKKIFGYNIYNVINMAIVIFTSIVTVIGLLGFFYKP
DNIKYEKISFKDIQILFYLACIMVGNLKIGITIYNAKAIWKLFNVAHESFLSNKYWKQNKH
KINNCGKKFARIFPWYFFMFLMTAFAWSIVPIVVNNHVTSKEIQNNENIYITNIANMRYPIT
AKTYNKFYKIFYVSEFIVVYYSAYGLAVFDLLILGFLQLMATHYETIASAYENFICKIENEN
GKLSNEEIQKEFVLIILDCQTVYEQLETIYGIARPIVLIYMVGDSIGMITMPFLIVMFYMQD
KSIFNSNVIAYSWTLFVVGIQLYMYCSLLQNVNEQKENINFGLYSCDWTWLDIEIKKLILL
AMRMNSSNNLKMNVTFTKFIDLPMFANIIRSSYSVTSVLINSNIHKINK
>RmaiOR43b-like
MPRIDAVNVFLRMTGCTDSKRMLYLTYFEFFITFYYLITTCVSIIHHEQSVSIKLFTLLCILIE
CVILLNITFRIYHQNQFCEMEQYSKRSGIPDDYQSKINIITMYHLIASNMFVIFPVTYAILSDS
VRVGDPFTFPFLDVLPIKTDNLVIYAGKYFVYAISVYIAHVELCFINTTFIYYVGVLKNRLE

AIVQTIREAVVDNDEQKMKYAVIQHQRLLTYFNIMKKVFTKPILLSMSFNAIYFGLTTSLVI
QAIRGYINQTILSICIASSIAAIINITIYTFYGSVLLDLHDEILHVLFDNSYFYLNKSFKSSILIM
MTRATIPLTFTVGYIFTINLNLLLKIVKMSYTVLNVLLSSEIIKPHKLS
>RmaiOR22b-like
MENCDYLSSAYNVLRWIGVFPSSDSHSTIWTRLAFNFYRVIIFIILALLTILMMVQMLVTTD
LTMLAKTIDIWTMFFSGLYKWIYMTMFNWEFVQLKTALTQLQTQGSMAYGRSADAFTAD
YLKQMQKISYWYLFGGIVAGIFIIVSPLLTYSKGDRSDFQYYNDPKSYPLSCWIPFTLNENY
MFLVVFVCHSIALFLIILVYLGIDTYFFGAIYAVGGQIELLNTSLNNNENNLAQLENSSCTNQ
TLICTEKQRMRCYSTLRECVKHHILVLDYIKNIRKLFSTLILMDYLHGITSLTFALFQLTISAS
VIETISVICFICMSVWHQYLNNFFGEFIIQKQLSVCTALYNVPWWRCDKRVRQLLTLMILRS
IKPTLISGYYMYKLSYESFISFVKALYTYYMVLRRVNMEDINA
>RmaiOR46a-like
MDHQKIMKKYEDFLALFGKAMLIQIFVSSLSLILIWFIFIMILSGEDRFVASEITTIKMVSLIP
SFSFQIFMVCYLFGNLHNQKDSIIFALYSSNWTEMDMKCKKMILFIMKMNNANYKKLKLT
STKIINLEMFFKTMGNCYTVISVLINQIKTTNE
>RmaiOR22a-like
MDNLKIEEVAINLKLLKLYRFYHMMKPNGTKIFNCNAYRLLFFLYGAIVNSMVIYSIIGFFV
DMDDSLAISDVDLFLILFVMINFFFCSWRISFILKKSITIGDALSISRFNFFTSEHCRKHFSVL
YDNRERTIKITNYFFIFSMIVLMQWIIFPIMVITFTAPDIEYYRLPNIMNLRFPVSTHTYNQY
YFIFYLMEVVISTFPIYVIMVTDTLILSFSLAIISQQEVLSRAFKNVGHKENSQSKCYEDFKSI
LGDHIQLNLKIKSYYSIVRPVILANVAMSSTFFIIVTYVLIVVCFSKESNQILTIIKLGSSALFI
SAQFFLYCYLLDRMNLKRESVNFAIYSCDWTKMDLKFKKLLLLTMRMNDANNLMIKISP
KKVVNLQMFTNVISMSYNIISVMLKTTNSKNVISE
>RmaiOR33b-like
MEHQEVMKKYEMFLALFEKVMLMQILFSSLSLIILWVILLMIFFDDNRFVNSEVTTLQMV
CLIPSYSFQIFMVCYLFGNLHNQKDSIIFALYSSNWTEMNINCKKMILFIIKMNNANYKKLK
FTSTKIINLEMFFKTMGNCYTVISVLINQIKTKNE
>RmaiOR22c-like
MRSPASATAVDETLFKTIGLHQLLCPANRGGYSACFRRALLIVLGLSFALHAFQVPWLYYA
LNDLQRFAYMAAVIIYGMMCAFKGYVLVTNADRLWSVLGAAGYAYTECGRRDPSWLRR
CGVTLSAMLRSFVALSYATLIVWIALPFFVDEFTAIINLDGTVTRYRTTIHNMQFPVPLALY
NSRPVWMLIYFTEVAVCIVNVFIWSMFDCYLVTMCFVLNAQFHTMSAGYVTLGRRRARP
SPPETPDTSVRMKLNDVESNHYDDLIGHIQDNQKLIKVYDVFFDVVRPVVLVQIGNGSYS
VISLIFLISLMYLMGIPVLSAPFLKFICGVISLTIELFIFCYGFNHIETAKSVINFGLYSSNWTE
MDLKFKKSLLLAMKMNSSHKRVMKISPNSAVGLEMFARVMNMSYSIVSVLLNSRS
>AcraOR1
MNFVYPATDQFYNDNFMIYYCIEFVFLILWCHSTMNFDVLFLSMNITFKYQLKTIANSFST
FNIAHYIKICYSHNHTKNVKHRKESELMLDFKSIIYDQQRVIENMRNIYQIFQPVVLTQLAI
ESIVIILQSCIIMMNYFNGISLISAMNLRLFAAIMTVTLQIYIICYVFDDVNQQKDSINFALYS
SDWTQSHAQHKNLLLHAMRMNNAENLRLQVTRKKICFLLSVLRNIILRHYIFCIKLLFTH
HAKSVFNTISAGKDVCKENAYFAFFSSNNFQALII
>AcraOR10a-like
MKSIRYNSITYKNCDYLSFAYNVIQWIGVFPPSDSTKWTRLVFNFYRAIIFIILTLFTTLMTV
QMFVTTELTILARTIDIWTMFFSGLYKWIYMTVFNWEFSHLKFSLAQLQTQGSKAYGRSA

DVFTADYLKQTKKISYWYLFSGFVAGFFIIVSPLLTYSKGDRSNFQYYNDPKSYPLSCWIPF
TLNENWMFLTIFICHSIALALIVLVYLGIDTYFFGAIYAVGGQIELLNTSLNNNENNLAQFEN
VSCTNQTSVYTEKQKMRCYSTLRECVKHHILVLDYIKNIRKLFSTLILVDYVHGITSVTFAL
FQLTISASVIETISVICFIFVSVWHQYLNNFFGEFIIQKQLSVCTALYNVPWWRCDKRVRQLL
TLMILRSIKPTLITGYYMYKLSYESFISFVKALYTYYMIFLDMRSASLLPILATILNTKIL
>AcraOR22a-like
MYLFNSRVLYILLSATKLCVMDNLRDEEVTIHLKLLKLFRFYHMIKLNSTKIFNYNYNAYR
LLTFLYVAIMNSIVIYSNIGFFVGTDDSQNINYVDLYLIMFLMTNIFFCSWRICIILIKSNTIFE
VLNVSRLNFFTSEHCRKHLNVLHKNRDQTIKITYYFFVFSTIVIIQWLISPIMAIMFTTTDVE
NVRLSNILNLRFPVSTHTYNQYHLIFYLIELTISIIPSYVIILTDTLILSFSLAIISQQKVLSRAFK
NIGHEENSQSKCYDDFKSILEDQIQLNLTLNINFMSIIYTYNVILKKKIKSYYSIVKPVILAN
VAMSSTFLVITTYVFIVKFLLNLISTGDMVLFSNESYEIFIIIKLGSTAFFIPAQFFLYCYLLDN
MYHKRESVNFAIYSCNWTKMDVKFKMLLLLTLRMNDANTLMIKISPKKFFNLQMFNSNI
NVLQYYLRHVENYKFKISLIARDCKDFTTKLVSQPPRGVFWVMLIG
>AcraOR22b-like
DIRQHFYIYYALVECCGISALFSTTKANNYKNNSKIDYFLMIFASTYVYLSIWKLFKCLKDR
KNFLDLFKIGKLDFLTSKECSKYSKVQYKHRDKNLKFVNYFLIFSFVVILQWFIFPIVINEII
NFENSENLKV
>AcraOR43b-like
MEHIVDMFLKKTGCNDDRSYDTMCQVFFIYFELAITLFFAVSSYLSIANSTEGLSVRLYGLL
CFLIESHIFFFIAVRLYYLPQFRDMYQRTLKMGIPENFRQRIAIVIKHHLIISNVFVSVSMLYT
ISMDWVQMGDPFTFPFIDVLPIKTTNLTIYVCKYILYTLPVYIAYFETCFLNVTFMYSTGVV
KRYFQILDGQVEEAIANKDEQQLKIAIKHHQEVLKFFDDMKTAYEKPILMTIEFCGLYVGL
TSYFSILVIQHDGILSALFEHRSVYSGNNSSFKRLISIMMTRATIPLEFKVASIFTINLNLLIKIL
KFVYTVFNVLLTSISRKLKETAI
>AcraOR4-like
MVGRSSFGSVDAMQQQPESFVLTPFQKFCIRWSVFFDSTSDRLSRIETVLRSVQLSTIMITS
VLTMTSVLIADNKKALESFTYFVICVFLLAIITFAIRTKRFNRAMLLMIVDEFPGYERPMPD
TLKRKMSAIRKSYGEFTMKVMVSYLTLVLFEIPATAMVPLTAARLTDVKLGSQSTQMVVL
WFPGDTTQIGTYAFSFVIQFLIVMIVKFIITGIMCSFSFFVSQMISEFQILSAYVEHAVEIVEY
DMSTGKTTDQKLLDHVKSCVMLHHRLIDFKDQLNESYGYIILLELMFSTLYFCLSAFNMIF
VGNRFVIAKGLLTLSNYLAELFIFCMYGSMVEEAHMGLLRASYSAAWYSQPVRFRRSLM
MVMSRTQTPLQLTIGKVFIANLPLFLSVLKVSYSGVNALRAANAK
>AcraOR42b-like
MATASSSEELTIVDNRLFKAISLHQILNPTHGGNKYYRIALLAIMWISLVVQSMQLVGLYFA
VNDLQRFVFTTTVVSNSFLSMSKAYVLVTNVDRLRDGLEAAQYEFTSCGSRDQRTVRRA
RAALSTIVRTFTVLSYVTCFMWAINPLSAIGEFLPVTNADGTVSRYRVTIYNVWLPVPVTV
YNTTAVWAVLYAVEVIVCFFNVISWLLFDSYVFTMCFTFKAHFSTLSASYATIGHPDALRSQ
TPHVSGTYYTTTVIKPVILLQIIGGSYAVITLIFLTSLTYLMGFSIISTPVLKAFFGFLAVTFQL
YLYCYVFNLIETEKTAVNFGLYSSNWTAMDLKFKKTLLLAMNMNSAHRRVMKVTPKSIIN
LEMFAKVMNMSYSIVSVLLNSRAGK
>SflaOR4-like
MDAQRPRARFHLTPFQKFCVRWSVFYDSSSDRLSRVETTLRVYQLTTIMVTSGLTMYPILF
EDNKKALESLTYFIICAFLLSLIVLAIRTKRYNRAMLLMVEDEFPGYGGPVPDALRRKMSA

IRSSYGRFTMHIIVSYLALVVFEIPATGMVPLVAASFTDAKLGSQSTQMVVMWFPMDTSEI
KMYAVSMVIQFFIVLTVKFIITGIMCSFSFFVNQMITEFQILATFIEHATEIVEYDRSTNRTTE
QKLLDHLKMCTKLHNRLIFYKDQLNESYGFIILLELMFSTLYFCLSAFNMIFVGNKFVMV
KGLLTLSNYLAELFIFCMYGSMVEDAHINLLRASYNSKWYAQPLRSRRSLMMVMYRSQT
PLQLTVGKVFIANLPLFLSVLKVSYSGVNALRAANAK
>SflaOR46a-like
MAMLVVCGTLSVYKGHVMVANADLIWDTLHVARYAFITCAGRDESELRRAGSLLRALLR
AFVAVSYQTLAIWIAIPWLTDDRVPVANGDGTMAEYRMNVNNLWTPLPVAVYNATAVWA
VVYAIEVFLITVNVFFWALFDCYLVTMCFVLNAQFHTIAAAYEKLAWSPSPHRHSGIRENN
DGFELDHYDNLILHIKDNQRIMMKFNDFFDIVQPVILVQIVNGSFLVITLIYLTLLMYFTGW
SIKSLPILKFFSGMASLTIELYIYCYAFNHIETKKNVVNFGLYSSNWTAMSIKFKRTLLATMK
MNAAHQRLMKITPISIVNLEMFSKVMNMSYSVVTVLLNSNSTQTKEME
>SflaOR49b-like
MDDDDHQNYIINLRLMKLTGFYQLINPNTSKYYGYSPYKVVATIEIMFGVFSVSVLILSSY
YYLYNTNELMNHFMLAVAIFFSTFKLFCVSRNSELIWDCMDMTSVQFLSYTGHRRDALRT
ARAKSITLSILFLLLWGSVTVAWCLSPFIVDGVYLDVEINGKVRQYRYNSLNYVYPVGERF
YNDHFLAFYAVEMLQVVSWGHATIAYDTFVISMCIAIQFQLKTIADSYSTLCYGTVSEAYG
NKRNIDGNEFETALSLKLLIHDQQNMLKKIRDIFRLFEPVTFVQLAAQSMLIIFQAYMIFIN
NYNGLSLVSVPVIKLLLTITPNIIHSFITCYLYGLINEQKESMNFALYCSDWTKINTKYKKML
LLIMRMNNAEKLKLKVSINRIVNLEMFSSIMHTTYSIISVLAKSYGNTKTK
>SflaOR43a-like
MKFLTLAYDILQWIGVFPQWIWSKWKLWVYSIYRIMIFVMIALFTVLMTVQMFVATDLTIL
ARTIDIWTMFLSGLYKWSCMSLFHEKFVKLKTKLIEIQVQGSVAFGRRSADIFTTNYLKQT
QKVTFGYMMSGLVAAGFLIMSPLLTYPKGGRSDFEYYNDPRSYPLSCWMPFMIDERWMF
WTIFISQSISLIVVVCMYLGIDSFMFGAIYTVGGQIELLNWALNSIKNSLEEIGSSTSTDQLIQ
YTEKQQMKCYSILRQCVKHHILILNYIKMVHTLFSSLIIIDYLHGITSVSFALFQLTISRGIGE
KISVISFIALSVWHQFLNNFFGEFIIQKQLSVSVALYHIPWWRAGKSVRQLLSLMISRSIRPT
FITGFYMYKLSYESFISFVKALYTYYMVLRRVNAEDKNA
>AgosOR43b
MVHYMAEMFFKKAICSDDDLHGRDAMRMVFFTYGELAITLFFAVSTYLSIVHSTEDLSVL
LYGVLCLIIQLLIFAFISFRLYHRSHFRDMYQRSRGMEISENSNRKIAAVIKHHLIMPNVFVV
ISALYKISSDRVHIGDPFTFPFMDVLPIETTSVAVYVCKYVVYALPVYLTQIEVCFLYVTYM
YSTSIMKSHFQILEKQVEEAMVNKDEHKLMIAIKHHQELLKFFKEMKTVYEKPIFLIIVSC
GLYIGLTSSLIIQVIQGFIHQILLGICIVSSLECALTIIIYCVYATNLYDLHDRILNALFQHQLLY
SQNKSFKQLILIMMTRTTIPLEFKAGSIFTVNMNLLVRILKFAYTVFNVLITSINHQLIKTAV
>AgosOR10a-like
MTVQMFVTTELTILARTIDIWTMFFSGLYKWIYMTVFNWEFAHLKFALAQLQTQGSKAYG
RSADVFTADYLKQTKKISYWYLFSGFVAGFFIIVSPLLTYSKGDRSNFQYYNDPKSYPLSC
WMPFTLNENWMFLTIFVCHSIALFLIVLVYLGIDTYFFGAIYAVGGQIELLNTSLNNNENTL
AQFENVSCTNQTSVFTEKQKMKCYSTLRECVKHHILILDYIKNIRKLFSTLILVDYLHGITS
VTFALFQLTISASVIETISVICFICVSVWHQYLNNFFGEFIIQKQLSVCTALYNVPWWRCDKR
VRQLLTLMILRSIKPTLITGYYMYKLSYESFISFVKALYTYYMVLRRVNTKT
>AgosOR43b-like

MPRIDAINVFLQMTGCTDSKRMLYLTYFEFLITLYYFIASYVSIIYYEQSVSIQLFTLLCMLIE
SYILLNITFRIYHKNQFREMDQYSKQLGIPDDYQSKINIITMYHLIASNMFVIFPVTYTILYD
SVRVGDPFTFPFLDVLPIKTRNLLIYACKYFVYAISVYIAHIELCFINTTFIYYVGVLKYRLET
IVQTIREAVVDNDEQKMKYAVIQHQRLLIYFNTMKIVFTKPILQSMSFNAIYFGLTTTLVIQ
AIRGYINQTIVSICIASGIAAIINITIYTFYGSVLLDLHDEILRVLFDNSFFYVNKSFKRSILIM
MARATIPLTFTVGYIFTINLNLLLKIVKMSYTVLNVLLSSEAIKPHKMS
>AgosOR2a-like
MYGSSCTDKIALSSENSATDVVGEICLTPRSDTVLTLVKIGSSVIYLTVRLFIYCYLFDSINK
KRELVNFSIYSCNWTKMDLKFKKLLLLTMQMNDANQMVMKASPKKIIDLQLFASIMSTSF
NMVPVLLKIKNSEYYKSQ
>AgosOR4-like
MVGHSLFASVDVMQQQPESFVLTPFQKFCIRWSVFFDSTSDRLSRIETVLRSVQLSTIMITS
VLTMTSVLIADNKKALESFTYFVICVFLLAIITFAIRTKRFNRAMLLMVVDEFPGYERPMPD
DLKRKISAIRKSYGEFTMKVMVSYLTLVLFEIPATAMVPLTAARLTDVKLGSQSTQMVVLW
FPGDTTQIGTYVFSFVIQFLIVMIVKFIITGIMCSFSFFVSQMISEFQILSAYVEHAVEIVEYDL
STGKTTDQKLLDHVKSCVMLHHRLIDFKDQLNESYGYIILLELMFSTLYFCLSAFNMIFVG
NRFVIAKGLLTLSNYLAELFIFCMYGSMVEEAHMGLLRASYSAAWYSQPVRFRRSLMMV
MSRTQTPLQLTIGKVFIANLPLFLSVLKVSYSGVNALRAANAK
>AgosOR42b-like
MATASPSEEESTIVDNRLFKAICLHQILNPTHGSNRYYRIAILECIWMSIVVQITQLVGLYYA
VNDLQRFAFTTTVVVNSFLSLAKAYVLMANVDRLRDGLEAARYEFTSCGSRDQRTVRRA
RAALSTLVRTFTVFSYVTCFFWMLNPLSAIGEFLPMTNADGTVSHYRVTIYNYWLPVSAT
VYNTTTVWALTYAVEMTVCFFNVNTWLLFDSYVLTMCFTFKAHFRTLSASYATIGHLDTF
RSLTPHASDDNFIKTGNNNTLNCYDELINHLLDNQRIIKKYDEFFEVIKPVILFQIIGGSYTVI
TLTFLTSLTYLMGFSIISIPVSKAFFGFLVLNFQLYLYCYVFNHIETEKSAVNFGLYSSNWTA
MDLKFKKTLLLAMNMNSAHRRVMKVIPRSIINLELFAKMMNITYSIVSVLLNSRAGK
>AgosOR33b-like
MDLSNLIVIIQDHQKLTKKIHDIFDEMRPIILFQLLSESILMSLIPLVLFLNSNNGISLTSTESIK
LLSAEIVNTGHLFSACYLFSLIDIYNDTINFALYNCNWTEMNINFKKLLLFTMQMNNANNF
KLNISTNIIVNLKLFTNLKFAVASYKTYAN
>AgosOR5
MPRIDAINVFLQMTGCTDSKRMLYLTYFEFLITLYYFIASYVSIIYYEQSVSIQLFTLLCMLIE
SYILLNITFRIYHKNQFREMDQYSKQLGIPDDYQSKINIITMYHLIASNMFVIFPVTYTILYD
SVRVGDPFTFPFLDVLPIKTRNLVIYACKYFVYAISVYIAHIELCFINTTFIYYVGVLKYRLET
IVQTIREAVVDNDEQKMKYAVIQHQRLLIYFNTMKIVFTKPILQSMSFNAIYFGLTTTLVIQ
AIRGYINQTIVSICIASGIAAIINITIYTFYGSVLLDLHDEILRVLFDNSFFYVNKSFKRSILIM
MARATIPLTFTVGYIFTINLNLLLKIVKMSYTVLNVLLSSEAIKPHKMS
>AgosORCO
MGYKKDGLIKDLWPNIRLIQLSGLFISEYYDDYSGLAVLLRKIYSWITTIIIYSQFIFIVIFMV
TKSNDSDQLAAGVVTTLFFTHSMIKFMYFSTGTKSFYRTLSCWNNTSPHPLFTESHSRFHA
KSLSRMRQLLIIVSIVTIFTTISWTTITFFGESVWKVPDPETFNQTMYIPVPRLMLHSWYPW
DSSHGLGYIVAFALQFYWIFITLSHSNLLELLFSSFLVHACEQLQHLKEILNPLIELSATLDSA
VHNPAEIFRANSAKNQPINGVDYNGSYVNEITEYGTKGETELNRKGPNNLTSNQEVLVRS
AIKYWVERHKHVVKYVSLITECYGSALLFHMLVSTVILTILAYQATKINGVNVFAFSTIGYL

MYSFAQIFMFCIHGNELIEESSSVMEAAYGCQWYDGSEEAKTFVQIVCQQCQKPLIVSGA
KFFNVSLDLFASVLGAVVTYFMVLVQLK
>DnoxOR46B-like
MQFPVPLAVYNSRPVWALIYVIEVYVCIVNVFIWSQFDCYLVTMCFVLNAQFHTMSAGY
GKLGSGRAESTSSDITSRTDVRRIKFDDVESNHYDDLISHIQDNQKLIKKFDVFFEVVRPVV
LVQIANGSYSVISLIFLTALMYLMGVPILSAPFLKFVCGLISLTIELFIFCYGFNHIETAKSVV
NLGLYNSNWTEMDLSFKKTMLLAMKMNTSHKRAMKVSPNSAVGLEMFARVMNMSYSI
VSVLLNSRS
>DnoxOR22c-like
MRFSCVADIKNDVSIKFPNHYDDLITHIKDNQKIVEKYEIFFDVVRSTVLLQITDGSYSVITL
IFLISIAYLNGDSIVSPTILKFVCGLASLLIELYIFCYGFNHIEDGRSTVNFGLYSCDWTNKDL
KFKKTVLLAMSMNSAHKKVMKLSPNSIVNLEMFSRVMNMSYTIVSTLLS
>DnoxOR85b-like
MYAFSYVIQFLIVVTVKFIITGIMCSFSFFVNQMISEFQILSAYVEHAVEIVEYDQSVDKTTE
QKLLEHVKNCVMLHNRLIHFKDQLNESYGYIILLELMFSTLYFCLSAFNMIFVGNRFVMV
KGLLTLSNYLAELFIFCMYGSMVEDTHMGLLRSSYSAAWYAQPVRFRRALMMVMSRTQT
PLQLTVGKVFIANLPLFLSVLKVSYSGVNALRAANAK
>AlinOR1
MSINDYKPGEIFQNNLKPMKKLRPLKKVSYQQPDKRGTYKKVAKRLSEEEALKKGFNDN
QGLYLVLGTLYRDSFGSWVHTIVFIIACLFMLFCLGRQTLLITDDLSLLFETIHYITIIGGVLV
IVPPMMKNQFRFQKIFKIFAREVYCYDYLDEETAQEILRLRAEGNKEKQLLTKAFTVMLLG
TFAGFSVLLPGMYIINGQFFAPQREDGVIMGIPCVIWFPFRVDDKWVVTVRILLLALEEYAS
FTVVAFIIGQQTTAICIGHTLLYEFKVLALTMNKFEQRAKLMDKKFIGDGTLKPASQTRKYI
TSCLNESIKHHDVLLDVSEQYSSIFYVPELVILLSSTMVICLSAVSLTSDNIPLEAKAVSVIFT
GAEMMNVFVNCYYGQILLDAHNIIGDAMYESNWTSYSSIVHQHVLIILSRVQKPLSLTAG
GFAAVNLDTFAQVVKSSFSYFSLLQALKE
>AlinOR2
MTVDELTLHDMVGFPLWIQNVLYMKITGHWVGAVPGPTPLRVNILRAVGGFPVFLVLLYV
AGANINGMVHNSDMTDISMNLIVLSTTVSALHKYSVFTNQQQALGRLGRWVKSVAAERK
ANNVPDTYADRVLKKALKAFYISGNVASPILIVKMMLTGNTFNVNPGVENFPKPFLMFLIA
VSFQAIAWEAVVDGCILMNSLFVFRSELVRFALEWEKLNFDPHNPEISRRQLKAMVKKHV
MLLGVKKDLKEYNNSMFGYQVFAAVFTTCALIYGCAKDTKFLGQAVIQVLPTSTASLLTF
SILCWSGEEVTYLFQQIHRNIYMTNWFEAPREDKKSIIVILEFAKNPIIFTGFTVFTCTLTTFV
ETMKQSFSLYTILKAVL
>AlinOR3
MTLKSYIKETLKWDEPLGLITTIAVVAGAFNTIAPPKRIRRFIYWLSWYQTISYILFLMSAGT
NIFTSTDFFDECLESLHFLVTAFHVFMKYLTLRFRERDFLELFDHIKRVWSSYRIHNEHFLTS
KLSSVNITSVLIFTSIFNVVFVNVGAAYLKNILDPTKVHLPIQIWIPSFTKSSFLVGTTIQVVL
FTWPLFIVAMSTTFLNSISSHVEALGLALAEDIGREKVWSRDVARDFYKKHQDVISIVLRV
NALMAGNWGFEMICASVQLTLPAYRTLRAFRMNDVEVFNHAVILCLNMMVIYMIFSSGN
RILSMGEKIHTKVYESNWFELPVKERKNVLFMLFRTTVPVEYRYKIIHFDLPGFTKVVNTV
FSYMALLRFLDSGGSEDEGALM
>AlinOR4

MGYVSSKFKSSVQEWHSWEDEYSVEAMRLRYRGFHRIGFLVLDLSPKYALLSVIMCVIAA
AVLFIVSFCLTFSCYQMSDDFEDCSGVCNLGFLCVLAFSFLLNHNFYRKKILDLHHMLGK
GFHDYQEPQYFPDELEKFKKVVTKQNVALIILASYVALIGFLVVVVCPLIDESLGFGWTEP
YDENGVNRQLPVPIWLPYPSHEGFLHWFSFLFLEGFGGAMICLSIGGTALLFTCLSGGLML
EQKLLVLSIKSIEKRAKRRYRELHKGKPGIDEDGNKIALNDDNKYQECIGYCLRQNILHHH
KILTYTNHYLDLARSPLLFAFLVETMAIAMSMVKLNEGSNKWGANIAFACIAIAEVANMI
MLCVLGELVTSGSIEINDELYYTKWYTFNKSNKKVLLQFLLETRNPVVLAALGLVVCNMD
QFSSVMHTAYSFFNMVKLSKLREETVTMGANT
>AlinOR5
MPPNQLLNTSKHEHDVYKRLDKLYYYGMRLLLLGITPHKFFGKCYFKGAILYVTVILLYIL
YGLGELLWAIIIPGGILERLSHAYVCSYCASYGVIWVYLMVKLETIHQNRIDFKSFNCSRLL
GNTEVESILQKNINYFVKTLFAASLLAGANVTSYILGFVVELIVQYAETGTLEEICVLSCFPF
PLWGQMIIAAANLVTLFMCFSIIMSMYIITGLLSLEIETQCEILTRTMAYDEVNDDFKTFVID
HIRLIKQTKWVVRLFENINNSLFFSSYVCLAMQMFSLSVIKPEGYYYLGVFSDFCVEFIVM
ASQCWLSSAVTNSVLSISEGVYNTPWYRKNKSNAIDVILMTQMAQRPYIQRVFLGTMKIE
KETMIQVIQQSYSFYALLMILQSKK
>AlinOR6
MTDEHLAGVSRIYRDALGFSQLDVFLDAKPPQNGRFSWHIKRIVAQFFVCFLAPSFISLQIC
GVLTAESQNLKQLSFDLGFLSHNVQNFVKMTYWLTHLKSVRSLCIDVSTFNVNKYRPILSS
WVLKKETDVTRKFMNRCFLISYGNLIFWVALPTIVSICNYFRYVAGVTEGQDSYIPRLSPT
RFPVDMSSLRNRLLVGFFEYGLITMGFVYFQPIDMFFSSIVNMVRTQFFILNSSLFEMPADL
EEFWGSRIPVQDTQPPMDLRLFVEDHQRLVRYGVQLRKFLNPVLGMVTVDCFNIMCSLLI
VITEILEGDMNFTALLELISGLLVILSSLVVFYTYTSTSGMLKEAEESVFEALYAHKWYGKN
DEHKKNVIFMQIRTESANKIPMFHIGDVGRDTFIEGLRMCYTYYNFLKQFK
>AlinOR8
MSLRTNAADGKNHLLDLRDVEGLTMGLNTFGIKTFWHILDYFHTTGKRHWLMTTYIVM
YHLIGGTYCLLGFAAVFFIDQEDIPRMAAAIMNPLIATQAIFKCWTFSYSTAEYLKLFVLLK
KNFMSCVPPEKKLAVDEVTKKNIGLTNQFVKYAMRWNCFTLSMVSFMPYLRSQAFREFF
HLGVGPIVPNKVFENEYPFEWNSSPTYEIIWFYEQICAFLAVVTSSAYQAILLYFVMAIVGH
LKVLGFVMGNMKATDFTSDSNETMDETAKAKSYKQLVLCIRDHGKINEAGDLLAERYNT
FLTFHIGIAIVVGIIAIFNCTVATELADKIKFGIMCVYGLLEVAIYCFCGQLLENACDDVLRQ
VYSCEWERMDPKFRKAAQLMMVRANSPICLRAGRLYRVNLETLEAIQQLVYTSLTMLTS
MVQ
>AlucOR2
MVLVPYLKPQKERNAAVDRGYDITGMFYARLAGLYPDLEIGWRYWFFGSYQILVVVAYF
YYVLAYVIANVIAIKYMDVELIGSTLCFGSYTYTYALIALTFYIKRSKIDKLLEIIGNELYIYQ
CPLSQKQLKIRNEETTRAKNFGRYSFFVPCLVALTHMSVVPAIHGFKGEYSSIVNGSAPINK
YTPLPVWTPVQATSGMSFFVVFWCQLCPGFVEFLIFHGSCTFFVGVVCVLVSEIKILLESLN
SITDRAKYLYHVKGGRGSDIDNLYDDPIYQQCMVDCLKENVKHHIKIKEFRNLFQDIISYCI
FFIFGGAAVTISTPPYTILKIMESGDTDKLYSAGVVMMGHTFLSVYLLSRYCKFGQNFESE
NSKLLEAFYCTPWYNTNMDYRKILIIAMSNSQKTLQIKGSVVGVSLSAAAFLDVIKSSYSL
LNFLATAGS
>AlucOR7

MIPFVFKRDDSVDHEVVKGYQSTYNYIMRFCGLYPDFRGFWYYISGAHLNTVHLAYIWFL
AAYMISTYYAFAYRDMDLLSYELCYGLVTLIWFTVTHYTIYKRDQLDSLFRKVGRGFFTY
EKPIDSEEEAIIDECNTNCRKTFQKTLALTTILAFWTCIIPPLPKAVMGDYSSIVEGGVPVNK
HLALPTWNPYPTDTHLTYWTMWMYQALAGCTEAYIIGATCILYCNFCTIINRELKLLRFSL
GNIKNRAIHAFKMRGYSLQLGQKYENSQLYQVCLVHCIDESIKHHIELKQFHGAIQNLLGF
PIFAIFSGSALTISSPMFMFLQMIGEHEESSFTLVMNIFQYTIIIFGFTYFLANYCLFGQSITDE
SALLHFAFYDTPWPEAGLNFRRKVLMGMIHSRKPFVLTAHGLASASSETLVDMLKTVYSY
FNLLAAT
>AlucOR12
MKFIDKLAEEEDDELIEILKGNYWHFLFYSMTFIRWKRPRIAIALISAYAIWIIVHLVIGIYSI
YLAADERNWAVVGLVTHHMVLGALAIYLPIFCNTGGFREVMADMHRTFTTDIGQYSGGN
MYAEQACIDIKKDVRRQTFVYYINPALVAAAGSLALAGPFLTKWFSGMENPYSPNGLSLK
LPTALYYPFPTDSGVVFYAIVLTQVISGTILGYLILAPQLVFINLSQNLKRELRFVGYSMETL
VRRAMRMTFENNVWKRKVTELDVDDTEFQQNVELSIKETIIHHQKASKLLSTAQVSVKG
PLAASYIFGLVTIAISLYNITLALKTNDIGSLTTFLLLLSSEVIGTFINCGLIGSELTEQSEDVTE
KLYFIEWYNFSVKNRKMFFTFQTAITQPYEIKAGGVTPMNMETFSDIMNSAYSFFNILQTIE
>AlucOR18
MSFSFVEKYQLSPETEKTMVTEYSYLLYVGGLLINYRPKVWIISIAQTSIFIGLITSYTIIFIIS
TAKSSNFVAFSQNLNYASLCCICLGLYFAGLSHRSAFVRLMEIIHDDFYDYGDSFDNAEVA
MWKSSLRTFKIIIVVGIPTYLIIIAVSIVLGDYIDTALGYDSTDEDYLGEIYQKAPLNLWYPF
VVTNMFLRVAVTLSQMTTAAILATTLATGDVMMLFLGQTVALQLRILCLAATKMDQRAN
LMYEKGLARSSSGDKEDLDGCYKLCIKQLVQHHLIIKEFYKTYYTIAKWPTAIAFMNGSL
MIAMSIIVAMNGNEETPSTYISTYLLLVAEVLSMWLLCETGQNVNTWSEKLFMDTYEFNW
NGLSVPNKKMLLIFKENIKKPLLMMAGGLTPINRDTFATIMNTSYSYVNLLRASERRSND
>AlucOR30
MVEKSNFHVKRAQLFKAYNSIHWLTLTKWFYEDYPVEKLWSDKRLWIHLSIVIICQSSITM
FKVFHLISEENFFIFLTSLTSFLVIVLVAVRTYILYQFPTFRQLYFKPEVFNCNLHRPTRSLALL
TEAITHSRKVGMWCLVLFITFDVAFLVLPIVPPILEIIDGTNKTYDELIPQYPSINPVSLSWLS
KELKYAFDLVMAVFNTIPWVGFVVVYYTVVQLFRAQHKIMMTAMLPGPPVPGDGREPLE
LKLWIQDHALIRKLVYKLRNTISPALAGTICVNVFTVGLNMLALVSSPIGSDAPMFTRYLY
YFSFGTYSALSIFDIFIHCWLASEITNCGEDLSYALLKSDWQNDLKRSHHHYVLPLMLCKK
QIRFTGLGLIPVTLTTFTETIRVSYSYFTLLRKTDD
>AlucOR46
MGYRVYPQQDLSDPSHMFSFQLNALKSTTMWKPDNQKYYIPFMILFAVNVFVLAICTVGL
LLKGCSTKDLVDRSEAMDIFTLTGSALYKMVFFLYHYEQLVDMVTCGLALVRNLPEGWT
KNCGLLSRIHYTAGFLVLLIWGLAPILKVMYGETTWAEMKLPINTYDPFDSTGFLFFLFYIT
GQYVLVLSAVIYMAADCYLFTSIYVAVGALQYIVDQFENMRDLNNNNKHTVADTMHDCL
QECIEIHVHVLDYLRKTDKLFKSMILADVVHAVISLSFAMLQTSESKGIFEGVKMVLFVQV
CFVHQFLNSHFGQELIDKQDNLAKQIITDIPWTDASRKFKKSYYIMLTCVREPFKLSAWNV
YFLQYATFLEFSKTMIQYYMVLQEVQDEAEVS
>AlucOR56
MVEDLTVKDLAGTYLLPHSVAYMHFTGHWIGAVPGPTPFRVKMYRAFGGTFTWLIVIATA
IASLNGMLHGSGMSDISMNLIIISTSISSLHKYSVFIHQEQGLGRLGRWMKRANEQNKISEN
PDTTTDRILKKSLVSFYYSGIVAASLLLVKIVVTGYTYNALIPGLDQRYQPLILVFMEAFSFS

SLSLEVIMDALILMNSLFVFRRELMKNVDEWRKMNYKSDNPQQFRQQLKTNVQNHVEL
LTIFQDVKNYCNSMFGYQVFAIVFTTCALLYGMAKDSGFDKAVLVQVMPTSLASFLEFFIL
CWCGEEIQHGFQQVHTSIYDTNWYEAPLREKKSMTIVLEFSKNTIQLTGFTVFKADLKTF
VESMRQSFSLYTLLQKLV
>AsutOR4
MVLVPFFIKPQIGRNAAIDRGYKLTSMFYARLAGLYPDLEAGWRYWFFGTYLNFVYVAYV
YYVSAYMIANVIAVKYKDFELIGSTFCFGSYTYVYALITIMFLIKRQKIGRLLEIVGNDVYK
YRRPPTKEEILIKETESMKAIVYGRYTFYIPCSVALMQMAVVPAIHGLRGEYTSIVNGSTPID
KYSPLPVWTPVEATTGLSFFVLYWCQLCPGFVEFLIFHGSCTFYIGVTCALVSNLKLLHHSL
GRIVDRAEYLYDIKNGKYDHQRKIPLNTELFDECMVECLKENVQHHVEIIKFHHLFQDIVG
YSNLFIFSGAAVTISTPPFTIIKIAELGDRHQLLTAGIVMIGHAFLSLFLLAQYCKYGQSIEDE
SEKILESYYFTPWFKASKSFRQVLVVAMSNSLKPLEIKSAVVGISASAATYMSIIKSAYSML
NFLVTAK
>AsutOR6
MFIFIREKETERNPDNMKGRKLLEARFAVFSGIYPDFYGWQYYYFAFFLIVIHPGLYTYYLY
AYGKSFYYGMVYADIELVGQVLCLGTITVIYCIVSIYYIARKTDMDDLITMVGKGFINYNR
GVTEQEQSIIDEMEKVTHRYAFGSTVMLTTISLVHMGLLPVIRGLKGQFTSVTNETAPINKY
TPLPVWMPYDCNSVGIFIFTFIFQMVPGCMEYAIINACCILYVGLAQQLSGNLKILANSIRDI
HTRALIMFDNEGGTVSKNTSELYANAHFLKCMNACLNENMEHHVKLIEFFNKFQGVAGF
SMLAIFSGTGLIISTAAYSLLLIAQTGGDKELLITNIFVWTFYLLVYTFLLMVYCYYGQEVT
DKNDEILPALYETPWLEADLPFRRSVLISMSYCQRSMQLSAMGLIYSSYATVLDIIKTAFSY
LNMLMAVQ
>AsutOR9
MSLRTNAADEKNHLLDLKDVEGLTMGLNTFGIKTFWHTLDYFHTTGKRHWLMTTYIVMI
HLIGGTYCLLGFAAVFFIDMDIPRMTAAIMNPLCGLQTIFKCWTFSYSTAEYLKLFVLLKK
NFMSCVPPEKKLAVDEVTKKNIGLTNQFIKYAMRWNCVTLCMVSTMPYLRSQVFREFFH
LGVGPIVPNKICENEYPFEWNSSPMYEIIWFYEQICVFLAVVTSSAYQAILLYFVMAIVGHL
KVLGFVMENMKATDFTSDSNETMDETAKAKSYKQLVLCIRDHGKINQAGDLLAERYNTF
LTFHIGVAIVVGIIAIFNCTLATELADKIKFGIMCVYGLLEVAIYCFCGQLLENACDDVLRQV
YSCEWERMDPKFRKAAQLMMVRSNSPICLRAGRLYRVNLETLEAIQQLVYSSLTMLSSM
VQ
>AsutOR10
MSPQSNAGDGKKHLLDLKDVEGLNMGLNSRGISIFWHTLDYFHTTGKRHWLMKTYIVFL
HLVGFTYCLLGFAAVFVIEMDIKRVTAAIMNPICGLQTVFKCWSFSYSTAEYLKLFELLKR
NFMTCVPPEKKLAANEVTTQNISVTNQFVKYAMRWNCVTLCMVSFMPYLRSQIFREFFH
LGVGPIVPNKICENEYPFEWNSSPVYEIIWFYEQVAVTIAIATSSAYQAILLFLVMALVGHLK
VLGFVMETMKASDFTGDSFQRMDESAKAKSYKQLIKCIKDHQKINHAGDLLAERYNTFL
TFHLGTAIIVGIIAIFNCTFATELADKIKFAIMCVYGLLEVAIYCLCGQLLENASEGVLRQVY
SCEWEEMDPKFRRAAQLMMVRANKPICLRAGRLYRVNLETLGAIQQLVYSSLTMLSSMV
Q
>AsutOR46
MIPFIFKKKDSNDPAVMRGYELTYSYAMRLGGLYPDFRGFRYYIFGFHLNCIHVAYLWFILA
YIISAYYGFVYNDMELVSYNICYGLLTIIFFLVGHSVMYKRNHLDRLFETVGKGFFTYEKPL
DADEQAIVDECDMKCKKTAKRNILLTMVLTTWTCVVPPLPKALKGEYSSVVAGGVPINK

HLPLPVWSPYPIDTPLTYWSMYALEFTAGITEAFIIATTCTLFCNLCTIVSRELKLLRLALRR
TRSRANYTFKMRGYSYLAGSNYAKYKLFQQCMVHCIEECIKHHIALKKFQEEFQGFMGF
PIFAIFSGTALTISSPMFMLLTMTEAEDSFLVLMKILQYSSIIFSFTCFLSSYCLFGQMITNESS
LVHFAFYETPWIDGDLDFRRKVIMGMIHSRKPFILTAKGLAAASSETLVDISKTIFSCFNLLA
ATQ
>AsutORCO
MQKVKMHGLVGDLWPNIRLMQLTGHWLLEYHEETGGMARLIRIAYCWMTTFIVYLQYA
FLVCFLILETYNSDEMAAVTITTLFFLHSVTKFTYFAIRSKYFYRTLSAWNQVNSHPLFAES
NARHRAAALSRMRKLLMIIGVVTILAVFGWTTVTFLDDPVWDKTDPDNVNETISVEIPQL
MVYAWYPWDAKTGMTYFMTFALQLYWLFITLAHSNLLDVLFCCFVIFSCEQLKHLKEILQ
PLMELSAALDSVVPNSGDLFKSGSAGSNIALISNGDGGNDFDVRGIYSSQRDFSGFQRGM
TNGTTVGPNGLTKRQELLVRSAIKYWVERHRHVVKFVASIGDTYGTALLLYMLTSTVTLT
LLAYQATKIEGVDVYASTTIGYLVYTLGQVFVFCIHGNELIEESSSVMEAAYSCHWYDGSE
EAKTFVQIVCQQCQKSLTVSGAKFFTVSLDLFASVFGAVVTYFMVLVQLK
>MsanOR2
MTKTPRVAAVFTAPESSEDLTIVDNKLFKAICLHQLLDPTNGSNRYYGLVLMAFMSVSLG
MQIIQLVGLYFAVNDLQRFAFATTTVSNSLLELFKGYVLVANADRLRASLEVARYEFTTCGS
WDQRLVRQSRAVLSTVLRTFTVLSWFTCFIWALAPLFAMDEYLQVTNADGTVSRYRVTIY
NVWLPVPTTVYNTTIVWTLVYAFEVIACFVNVFIWLLFDSYVVTMCFTFNAQFRTVSTSC
MNIGHRDHCFRSPSPHASEGASDDNDILNCYDELINHIKDNQSLIKKCDDFFEIIKPAIIFQII
GGSYSVITLIFLTSLTYLMGFSIISGPVLRSFFGFLSLTFELFLYCYVFNHIETEKCKMNFGLY
SCNWTAMDLKFKKTLLHAMNMNTAHRRVMKVTPMSIINLEMFANVMNMSYSIVSVLLN
SRPQK*
>MsanOR43b-1
VDATSAFSKLETEKLSHSVCQWKRVIAHMVYNFFQKTGRSDVRRYATTCVEFFSYFELVVI
LIFVIFAFLSVVYSNEDLYNRIYSFSWLCFEANVFAVILIRLYYQSKFRDMYDRSQLIGGTLG
NYRRTLVILVVYYFVTSTALVIVPLLYVISFDSAEMGDPFSFPVADALPKKTGNVTVYVCK
YIVYSFILYAAHLENGFVNGTFIYFTGVVKKYFQILDVEVQEAMVNKDEQKLKIAIKRHQE
LLKLFIDMKTVYDKSILIIIEFCGLFNGFNGYIMIQCIQGVIHPKILGFSIACILASLLTMSIYCI
CGSNIYYLHDGLLKSLFEHNSCYSKNNSFKKLVIMMMTRATVPLEIKAGSIFAINLNLLVKI
LKFTYTVFNLLLTSVNRQIKENSAI*
>MsanOR43b-2
MADIVDTFFQYTGWSDDHSFVATYSAILTYSDLAVFLFFLIFTFWSIVYWTEDLSIRIYCFL
WLFIVVHMFVSIYSRLYYQSIFRDMYRHSLIVGLPENYRRKILIVIIYYFIVSNVYVYIPMVY
TIAFDSVQMGDPFTYPFMDVFAVKKPTITVYLCQYIIYAIPVYLTHLEGCLLNTTFMHSTGV
MKRLFQTLDKQVEEAMIIRDEQKLKIAIKQHQELLKFFRKMETVYEKQILLSIEFCGIYIGL
TCFIMIQVIQGVIHPLLLGLCIGSIFEGLASITVYCIFGSIMHDLHDGTLNSLFNQQSCYFRD
KSFKQLVVIMMARVSIPLEFKAGSIFTINLNLFVKILKFTYSVFNLLLTSVN*
>MsanOR46a-1
MMDIRDDQNNLFNITLAKYMGIYQMLDPQTTRFRGLNVYHIVMIFIILFLCVFAVIINISVV
YYWTDNMLLSIDFIWKGMIMLYACCPIWVIVNYSNDIWDCLSITCYGFTSYSLRYRHILDR
CKERSVFITTAITFLYFTSFVIYIVSSLTLLNDIIPVKNRDGSISNYQHNIINLYLLVSGDTYNA
HYNIFYMLEVLILVFVIIPYCIFDFVTVTLCLAIRCQWQMICTAFESIGHTSLGDNLSLVDCG

EEKKKLPNKHDLIYDELKTIIMNHQAVKKKYDKFLTIFQQALLLQIVVCLTTFIILWICFILV
KINMMFNQKKFFSA*
>MsanOR46a-2
YMGIDFLWKSENTLYVIYKAYFIVRYSKDIWKCLSITRYDFTSFKYQNRHILDYWRKRLTR
LTTIYAIMHLTTVSSFLLISLAFTKYKLPVKNHDGSIGYYRHNVMNLYIIVSDETYNAHYY
MFYIIETLFVSLVTSIFFTFCLLLITLCFSICCQMQVICSAFESVGHKSLRDLDSPIGDDYTAE
NIKIPSNEHDLNYNELKTIINDHQAVMEKYEMFLTLFRRVILLHIFVSSLLAITLLLTIIMSFS
NDDRYKTSEVVIGKLLCCIPSIFFDIFVVSYLFGNIHDHNDSMVFALYGSNWTEMDMKCK
KLVLLTMKLNNA
>MsanOR64
MDIWKNKKHLFNIKLSKIITLYQILDPETVKYHGRNIYHIVIACTMLCTSIVLMIGVLSGLN
YFKSNIPLSMNFFFLISFGAYTIYKIWFTIHHSNDIWDYLSITQYDFTSIGNRHRHILDRWRE
RLTWLTNIYAILVLMLTVYFIAMTSAFSEDKSLIKNHDGSIGYYRQNVLNLYLIVSDETYNT
HYYTFYIVEASVFVYLSIYFCLFDILLITLCYSMCCQMQIVCSAFELVGHRSLRDHHSPIDQ
SVGNISTNEHDLIHEELKTIIMDHQLVMKKYEDFVSLFRRMILLHIFVSSLLVIVLIFTITMSF
SSDERFKTSTNAVHKLFVFIPPTLFQIFMVCYLYGSMHNQKDAIIFALYSSNWTEMDMKCK
KLILLTMKLNNANQKK
>MsanORCO
MGYKKDGLIKDLWPNIRLIQLSGLFISEYYDDYSGLAVLFRKIYSWITTIIIYSQFIFIVMFM
VTKSNDSDQLAAGVVTTLFFTHSMIKFMYFSTGTKSFYRTLSCWNNTSPHPLFAESHSRFH
AKSLSRMRQLLIIVSIVTIFTTISWTTITFFGESVWKVPNPETYNQTMYVPVPRLMLHSWYP
WDSSHGLGYIVAFVLQFYWIFITLSHSNLLELLFSSFLVHACEQLQHLKEILNPLIELSATLD
SSVHNPAEIFRANSAKNQSINGIDRDYNGSYVNEITEYGTKGENESNRKGPNSLTSNQEVL
VRSAIKYWVERHKHVVKYVSLITECYGSALLFHMLVSTVILTILAYQATKINGVNVFAFSTI
GYLMYSFAQIFMFCIHGNELIEESSSVMEAAYGCHWYDGSEEAKTFVQIVCQQCQKPLIVS
GAKFFNVSLDLFASVLGAVVTYFMVLVQLK*
>MsanOR4
SYQAVVKINKMTSIIGKKNYQRKFYQTLMTLAFFLDTSQYRFISRFIMQFYIFDWMVLVSL
AAAFTILEENSTTTLIMELIQYIIVGIYFSLIFVVFIFKKEAIMSNYDCIQTKFIQWSNKRALH
PNAAYKENIKTFKSLSIPLAILSLSIAFGPLVSTFNDIGKLPLDNRAHFVLFWPKIVDTNKPS
MYGIIYTLQVIFTISLYISVLSLNLGFMVFLNELTTQFEMLLDGINDAFKFKMDKQFPSLFID
CIRHHQIIIKFLDDLKSYFKWMILIEIIVLQVILSILIYNLTKVNASIGYKMKVGGSLLFNILPI
CFHCHIGEVVLSLHKRLSNHIYNMTWYDMPNKNKQLIVIMFQRTQRDLTLSSALFSSEKA
SRALISKVIKQVYTILNVLLKT*

File S4. The amino acid sequences of 39 ionotropic receptors (IRs) from 9 Hemiptera species.

>SflaIR25a.X1

MLTMYTLIRTTTILWITNLLDVGWSQNLQTINILFISDRNNLVAEETLNVALNYIRRNPRIGL
MLDGIYSVKIGGDDASEILETLCVNYNASIRNNKPPHLVIDTTINGVTSEAVKSFTAALALP
TVSASYGQSGDIRQWRNLDGEQQKYLIQISPPADIIPEIIRSIVVAQNITNAGIMFDDTFVMD
HKYKSLLQNIPTRHIIASIDDTTSIKLHLSRFRDVDIVNFFVLGKLSIIKSVLDHANSNKLFG
RKYAWHVITQDKGSLKCGCSNATILYVKPEPDAGSREKLSNLKTTYGLTSTPELKAAFYFD
FYYRSLLAIRSMMNSVEWPTNMTYTTCDEYNEENPLPRRNIDLRRYLKDMTEPPSYAPFLI
DTNGHSYEEFTMRLEKVTVLNSQSVSAENVGSWKASLNSPIVVKDAANMSHFSAVTVYR
VVTVLQNPFMIQVDDEDGKGVVKFKGYCIDLIEEIRKLIGFEYEIYIAPDNNFGNMDENGQ
WNGMVKELVEKRADIALGSLSVMAERENVVDFTVPYYDLVGITILMKKPQTPTSLFKFLT
VLENDVWMCILGAYFFTSFLMWVFDRWSPYSYQNNRNKYKDDEEKREFNLKECLWFCM
TSLTPQGGGEAPKNLSGRLVAATWWLFGFIIIASYTANLAAFLTVSRLDTPVESLDDLSKQY
KIQYAPLNGSATMTYFQRMADIETRFYEIWKDMSLNDSLSEVERAKLAVWDYPVSDKYT
KMWQAMKEAKLPNTLEEAIERVKSSKSSSEGFAYLGDATDIRYQVMIDCDLQMVGDEFS
RKPYAIAVQQGSPLKDQFNNAILLLLNKRKLEKLKETWWNMNPERIQCEKQDNQSDGISI
HNIGGVFIVIFVGIGLACFTLAFEYWWYKYKKSSKVANTMNPKQMVIGRGEFTYPIIPNYE
TSGMRSRNIIQGLRRSISQSTPKQQ

>SflaIR25a.X2

MFDDTFVMDHKYKSLLQNIPTRHIIASIDDTTSIKLHLSRFRDVDIVNFFVLGKLSIIKSVLD
HANSNKLFGRKYAWHVITQDKGSLKCGCSNATILYVKPEPDAGSREKLSNLKTTYGLTST
PELKAAFYFDFYYRSLLAIRSMMNSVEWPTNMTYTTCDEYNEENPLPRRNIDLRRYLKD
MTEPPSYAPFLIDTNGHSYEEFTMRLEKVTVLNSQSVSAENVGSWKASLNSPIVVKDAAN
MSHFSAVTVYRVVTVLQNPFMIQVDDEDGKGVVKFKGYCIDLIEEIRKLIGFEYEIYIAPD
NNFGNMDENGQWNGMVKELVEKRADIALGSLSVMAERENVVDFTVPYYDLVGITILMK
KPQTPTSLFKFLTVLENDVWMCILGAYFFTSFLMWVFDRWSPYSYQNNRNKYKDDEEKR
EFNLKECLWFCMTSLTPQGGGEAPKNLSGRLVAATWWLFGFIIIASYTANLAAFLTVSRLD
TPVESLDDLSKQYKIQYAPLNGSATMTYFQRMADIETRFYEIWKDMSLNDSLSEVERAKL
AVWDYPVSDKYTKMWQAMKEAKLPNTLEEAIERVKSSKSSSEGFAYLGDATDIRYQVMI
DCDLQMVGDEFSRKPYAIAVQQGSPLKDQFNNAILLLLNKRKLEKLKETWWNMNPERIQ
CEKQDNQSDGISIHNIGGVFIVIFVGIGLACFTLAFEYWWYKYKKSSKVANTMNPKQMVI
GRGEFTYPIIPNYETSGMRSRNIIQGLRRSISQSTPKQQ

>SflaIR2.X1

MSADAMLTNGGGGPCWLLVTVVWSLHVLDDSAAVAALPPVIRIGAIFTQEDKDSTTEVAF
KYAVYRINKDKAILPNTTLVYDIEYVSQQDSFKASKKVCRLLESGVHAIFGPSDSHLAGHI
VSICESVSMPLLLTIADGVTDLTGRHFVTDMFPAREHLGQAFRDLINFLNWTKIAIVYDDE
EGLLLVQNLMKMSKADFYIRQVDQHTHRQVMREIKNKHIFNIIVDIHPRNINGFFRSILQL
QMNDYRYHFLFTTFDLETFDLEDFKYNSVNMTSYRIVDDENHRVVNVLREMERFQRVGQ
NMLHKSGIIRAEPALMYDAVNVFANSIGSFEGSSDSMKSANISCKSSNRWANGTLLYNRLN
AVVIEGLTGRVHFDEGRRSDIKLDLLKLHQEKVKKVGFWTPSTGINITRHSVFYGQQSSNV
TLIVVTRVEKPYVMIKEDKNLTGNSKYEGFCIDLLHRIASQVGFHYAITLVPDNKYGAYDP
TTKQWNGIVRELMDKKADLAMASMTINYARESVVDFTKPFMNMGIGILFKVPSNEPSKL
FAFLNPLATSVWSFMLLAYMAVSFSLFFLARFSPYEWRPHTEENYRENRFTISNCFWFIAG
VSLKQDAGITPKATSARILGGIWWFFTIIIIPSYTANLTALRTVERLQKPIQNVAELSSQEKISF

GTLEGGSTMSFFRDSKIPIYQKMWKFMEKYPSVFVSTYEEGTKKVLGGNYAFLMESTMID
YAVQRDCNLTQISGLLDSKGYGIATPKGSVWRDKLSLAVLELQEKGVIQMLYDKWWKNA
ADICIKDEKVKEFKPKPLDLNDLGGVFVFVLCGLTVAVIIAILEFCWHSKKKSTDQQQSVC
TEMAEELRYAIRCDGTKHKATLKRTCNGCSPITTYVPAPMHMNHVTSQIDNVPMMELTKP
SISIDHEDK
>SflaIR2.X2
MVPRGNGPVWRYHRYHFISPSGRGSHQPLLRSGDSRILGAIFTQEDKDSTTEVAFKYAVYR
INKDKAILPNTTLVYDIEYVSQQDSFKASKKVCRLLESGVHAIFGPSDSHLAGHIVSICESV
SMPLLLTIADGVTDLTGRHFVTDMFPAREHLGQAFRDLINFLNWTKIAIVYDDEEGLLLVQ
NLMKMSKADFYIRQVDQHTHRQVMREIKNKHIFNIIVDIHPRNINGFFRSILQLQMNDYRY
HFLFTTFDLETFDLEDFKYNSVNMTSYRIVDDENHRVVNVLREMERFQRVGQNMLHKSG
IIRAEPALMYDAVNVFANSIGSFEGSSDSMKSANISCKSSNRWANGTLLYNRLNAVVIEGLT
GRVHFDEGRRSDIKLDLLKLHQEKVKKVGFWTPSTGINITRHSVFYGQQSSNVTLIVVTRV
EKPYVMIKEDKNLTGNSKYEGFCIDLLHRIASQVGFHYAITLVPDNKYGAYDPTTKQWNG
IVRELMDKKADLAMASMTINYARESVVDFTKPFMNMGIGILFKVPSNEPSKLFAFLNPLAT
SVWSFMLLAYMAVSFSLFFLARFSPYEWRPHTEENYRENRFTISNCFWFIAGVSLKQDAGI
TPKATSARILGGIWWFFTIIIIPSYTANLTALRTVERLQKPIQNVAELSSQEKISFGTLEGGST
MSFFRDSKIPIYQKMWKFMEKYPSVFVSTYEEGTKKVLGGNYAFLMESTMIDYAVQRDC
NLTQISGLLDSKGYGIATPKGSVWRDKLSLAVLELQEKGVIQMLYDKWWKNAADICIKDE
KVKEFKPKPLDLNDLGGVFVFVLCGLTVAVIIAILEFCWHSKKKSTDQQQSVCTEMAEELR
YAIRCDGTKHKATLKRTCNGCSPITTYVPAPMHMNHVTSQIDNVPMMELTKPSISIDHEDK
>SflaIR2.X3
MYTSHRWSNLIRRNSITCAIFTQEDKDSTTEVAFKYAVYRINKDKAILPNTTLVYDIEYVSQ
QDSFKASKKVCRLLESGVHAIFGPSDSHLAGHIVSICESVSMPLLLTIADGVTDLTGRHFVT
DMFPAREHLGQAFRDLINFLNWTKIAIVYDDEEGLLLVQNLMKMSKADFYIRQVDQHTH
RQVMREIKNKHIFNIIVDIHPRNINGFFRSILQLQMNDYRYHFLFTTFDLETFDLEDFKYNS
VNMTSYRIVDDENHRVVNVLREMERFQRVGQNMLHKSGIIRAEPALMYDAVNVFANSIG
SFEGSSDSMKSANISCKSSNRWANGTLLYNRLNAVVIEGLTGRVHFDEGRRSDIKLDLLKL
HQEKVKKVGFWTPSTGINITRHSVFYGQQSSNVTLIVVTRVEKPYVMIKEDKNLTGNSKY
EGFCIDLLHRIASQVGFHYAITLVPDNKYGAYDPTTKQWNGIVRELMDKKADLAMASMTI
NYARESVVDFTKPFMNMGIGILFKVPSNEPSKLFAFLNPLATSVWSFMLLAYMAVSFSLFF
LARFSPYEWRPHTEENYRENRFTISNCFWFIAGVSLKQDAGITPKATSARILGGIWWFFTIII
IPSYTANLTALRTVERLQKPIQNVAELSSQEKISFGTLEGGSTMSFFRDSKIPIYQKMWKFM
EKYPSVFVSTYEEGTKKVLGGNYAFLMESTMIDYAVQRDCNLTQISGLLDSKGYGIATPKG
SVWRDKLSLAVLELQEKGVIQMLYDKWWKNAADICIKDEKVKEFKPKPLDLNDLGGVF
VFVLCGLTVAVIIAILEFCWHSKKKSTDQQQSVCTEMAEELRYAIRCDGTKHKATLKRTCN
GCSPITTYVPAPMHMNHVTSQIDNVPMMELTKPSISIDHEDK
>SflaIR2.X4
MYTSHRWSNLIRRAIFTQEDKDSTTEVAFKYAVYRINKDKAILPNTTLVYDIEYVSQQDSF
KASKKVCRLLESGVHAIFGPSDSHLAGHIVSICESVSMPLLLTIADGVTDLTGRHFVTDMFP
AREHLGQAFRDLINFLNWTKIAIVYDDEEGLLLVQNLMKMSKADFYIRQVDQHTHRQVM
REIKNKHIFNIIVDIHPRNINGFFRSILQLQMNDYRYHFLFTTFDLETFDLEDFKYNSVNMTS
YRIVDDENHRVVNVLREMERFQRVGQNMLHKSGIIRAEPALMYDAVNVFANSIGSFEGSS
DSMKSANISCKSSNRWANGTLLYNRLNAVVIEGLTGRVHFDEGRRSDIKLDLLKLHQEKV

KKVGFWTPSTGINITRHSVFYGQQSSNVTLIVVTRVEKPYVMIKEDKNLTGNSKYEGFCID
LLHRIASQVGFHYAITLVPDNKYGAYDPTTKQWNGIVRELMDKKADLAMASMTINYARE
SVVDFTKPFMNMGIGILFKVPSNEPSKLFAFLNPLATSVWSFMLLAYMAVSFSLFFLARFSP
YEWRPHTEENYRENRFTISNCFWFIAGVSLKQDAGITPKATSARILGGIWWFFTIIIIPSYTA
NLTALRTVERLQKPIQNVAELSSQEKISFGTLEGGSTMSFFRDSKIPIYQKMWKFMEKYPSV
FVSTYEEGTKKVLGGNYAFLMESTMIDYAVQRDCNLTQISGLLDSKGYGIATPKGSVWRD
KLSLAVLELQEKGVIQMLYDKWWKNAADICIKDEKVKEFKPKPLDLNDLGGVFVFVLCG
LTVAVIIAILEFCWHSKKKSTDQQQSVCTEMAEELRYAIRCDGTKHKATLKRTCNGCSPITT
YVPAPMHMNHVTSQIDNVPMMELTKPSISIDHEDK

>MperIR25a

MFMTRIHIFLLWLTVVYTNGEAYKIGILTQSEDDTNFVQLKSIMTNYNVSIEPILNDPSYNITN
KFCSIASNNTLATIDLLIPSCIPCWKISNANSMAYFRTDFSYIQPTIQLIESFVTWLNITKEITFI
FTNQEYANQAVTYLTSGKSSLRAIVLSHLTSNEIDQLKNTKIGIRHVALIGNNLDQYVQIIN
QEKLIKLDESWIIVTNDTTKLKLESSVTLMKFTSWENGYTNKMTRARTLFNFIYYFLGSVS
RDRSKLSCDLISDVLVLEKRKEIEDKLNSYENKSELQYDIDTNRMTYNEQAMIFRVSQDG
VPNQLGTWTINGGLEMKHNASTVVSGRRFFRVGTAMSIPWTFMEDVWKGYCIDLIEKLS
KEMNFKYELVVKDKFGSLDPETNQWNGLIGGLVEGELDIVIAALTMTSEREEVIDFIAPYF
EQTGISIVIRKPSRKTSLFKFMTVLKPEVWLSIVAALAMTAVMIWILDKYSPYSAQNNRTK
YEQFRHFTLVESFWFALTSFTPQGGGETPKAISGRVLVAAYWVFVVLMLATFTANLAAFLT
VERMQTPVQSLQQLARQSRINYSVIDGSDAHHFFRNMKMAEDILYNVWKEIALNQTNNR
KDFRVWDYPIKEEYGQILAAIEKTGTVPNRSVGYQMVLDNEQGEFALIHDSSDIEYEVYN
NCNLTEVGEIFAERPYSIAVQQGSLIQEEISRKILDLQKDRFFELLNAKYWNASKVSMCPNA
DDSEGITLESLGGVFIATLVGLLIALITLAFEVVYFKHKRAKVAEVSVVNNTIHKDKLMYG
HELFMTLGRNSNSDDQTRWVNKIKLDSTTGRLNNALFFRRNKFQN

>MperIR2-like

MESGIGAVFGPSSLESSNIVQSLCETMEVPRIDTSWENTPVENFNFYFNPYPEPTLLAKGYT
AIVHDMDWKSFTLLYQRPECLQRLQDLIQDYSGKTKPNDKQVAAISILQLPEGNNFRPILK
DIKKSLEGHIVLDCDADLILTVFKQAKEVNLLDDYHSFIITSLDAHTVDFSGIVQNLRTNIT
TVRLIDPLSPFVENIVRDLNFVQQRMNVNMEPLKADKLTVNSILIYDAVNVFAKALKGLG
MINKIITEPLQCKNSPFIPWSNGFKLINFMRVIETEGLTGLLRFDNKTGHRSYFTLEMVELV
DTGFKKIGLWDPERGMTYTRTSLEMLRDLYAGSKNKTFIVSTKITEPYLMLKEGHNKLEG
NDKYEGYVVDLIQMIAKENNMTYEFRLRSDGNGKRDKKTNKWNGIIGEVQEMRADLGI
CDLTITHERRSAVDFTMPFMNLGISILFSKPEEPQTNLFSFTQPLSFQVWIFTATAYLGLSLVL
FFLARITPNEWQNPHPCNPHPQELENSLSLLNCLWFSMGSILCQGSDILPRAFPTRLCAAM
WWFFALIMTQSYTANWTAFLTSNRMETTIKNVEDLDKKGGTDSIKYGCVTDQSTASFFQV
GARCTLHTYLF

>MperIR1.X1

MKLKIVVWIIHFASRVTALPDTIRIGGLFHPNDINQENVFKHAIHDVNANRHILSRSNLSGQ
VEKVSPQDSFHASKRVCSLLRLGVAAVFGPQSAQISSHVQSICDTMEIPHLETRWDYKLRR
ESCLVNLYPHPTVLSKAYLDLVKKLGWKSFTIIYESNEGLVRLQELLKARNGALSAYPITIR
QLGSGRDHRPLLKQIKNSAESHVVLDCSTEKIYDVLKQAQQIGMMSDYHSYLITSLDLHTI
DLDEFKYGGTNITGFRLVNPDTPVVQKVLKQWGENFTVMSTETALIYDAVHLFARALHDL
DSSQKIDIKPLSCDASDTWSHGYSLINYMKIVEISGLTGVIKFDNQGFRTDFELDVVEVNKE
GLSKIGTWNSSQGINFTRSFVEAYSSIVDNLHNKTLVVTLILSSPYTMRRESSQKLVGNDQF

EGYAIDLIYEISKLLGFNYTLKLVPDGRYGSYNEDTKEWDGMMGELLQQRADLVVADLTI
TYDREQAVDFTMPFMNLGISILYRKPIKQPPNLFSFLSPLSLDVWIYMATAYLGVSVLLYIL
ARFSPYEWENPHPCNSEAPDVFENKFSLNNSLWFTIGSLMQQGSDMAPKAVSTRIVAGM
WWFFTLIMISSYTANLAAFLTVERMDSPIESAEDLAKQTKIKYGALRGGSTAGFFRDSNFIT
YQRMWSFMESSRPSVFMASNNEGVERVVKGKGNYAFLMESTSIEYVIERNCELTQVGGL
LDSKGYGIAMPPNSPYRTAISGAVLKLQEIGKLHKLKTKWWKEKRGGGACRDDTSKSNS
AANELGLANVGGVFVVLMGGMGVACVVAVFEFVWKSRKIAVEERIIKYHTKYRK
>MperIR1.X2
MKLKIVVWIIHFASRVTALPDTIRIGGLFHPNDINQENVFKHAIHDVNANRHILSRSNLSGQ
VEKVSPQDSFHASKRVCSLLRLGVAAVFGPQSAQISSHVQSICDTMEIPHLETRWDYKLRR
ESCLVNLYPHPTVLSKAYLDLVKKLGWKSFTIIYESNEGLVRLQELLKARNGALSAYPITIR
QLGSGRDHRPLLKQIKNSAESHVVLDCSTEKIYDVLKQAQQIGMMSDYHSYLITSLDLHTI
DLDEFKYGGTNITGFRLVNPDTPVVQKVLKQWGENFTVMSTETALIYDAVHLFARALHDL
DSSQKIDIKPLSCDASDTWSHGYSLINYMKIVEISGLTGVIKFDNQGFRTDFELDVVEVNKE
GLSKIGTWNSSQGINFTRSFVEAYSSIVDNLHNKTLVVTLILSSPYTMRRESSQKLVGNDQF
EGYAIDLIYEISKLLGFNYTLKLVPDGRYGSYNEDTKEWDGMMGELLQQRADLVVADLTI
TYDREQAVDFTMPFMNLGISILYRKPIKQPPNLFSFLSPLSLDVWIYMATAYLGVSVLLYIL
ARFTPYEWYNPHPCNPDPDNLENQFSLMNCMWFAFGSLMQQGCDILPKAVSTRIVAGMW
WFFTLIMISSYTANLAAFLTVERMDSPIESAEDLAKQTKIKYGALRGGSTAGFFRDSNFITY
QRMWSFMESSRPSVFMASNNEGVERVVKGKGNYAFLMESTSIEYVIERNCELTQVGGLL
DSKGYGIAMPPNSPYRTAISGAVLKLQEIGKLHKLKTKWWKEKRGGGACRDDTSKSNSA
ANELGLANVGGVFVVLMGGMGVACVVAVFEFVWKSRKIAVEERIIKYHTKYRK
>MperIR40a.X2
MATTAIPERDDYRHHLNILRRQRRAVVGGTGGGGWRDLDYGNLTNAIRDIVEAMTVDCT
LAVHSGVENAAADFLSNTIKSLHGRGVTTTHHALLSEDHVQSLLIDIRRAVADGHHTSYIV
LSTSALMENLLSAIRRSNLMSRNVVYVFLWLRSSVSRTFKADILEAMRVCVITSPRLGFYQ
IYYSQASARPGYGSSLKMVNWWSAMDGLVRFPLLPPPKQVYKNFEGRYFNVPVLHKPP
WTFVEYLNDSFRVEGGRDDKLINLLADKLHFQFRYIDPPDRTQGSGLDRGSSMQGVLGLI
WQREADWFVGDLSITYERNLVVDFSFLTLVDNEAFLTHAPGRLNEAFSLIRPFHWSVWPL
LLITVIFAGPILYILVDTTDGHPQGKSMLYWKCVWWSVTVFLQQAAIIPSENNKIRFVAGLL
MLSVTYVIGDMYSASLTSILARPPKEPPINTLNELSEAMRDSGLQLLVEVQSASQAMLENG
TGVYEELSQLVTRQREYLIGSTEKGMQLVRDNKNYAVIGGRETFYYDIKRFGAQHFHLSE
KLNTRYSAIAFQRACPYRDNFDDVLMRLFEGGILSKITEEEYQKLNDKLMGSEKFDSTSV
VIEPVLEGSEPRQEDDDKQLTIAMSMKTLQGAFYVLAIGSILAGFLLLIEMRSHDKFEKDK
RIKRVEAPFVYKRKVPNKFQNRLYDLKE
>MperIR40a.X1
MATTAIPERDDYRHHLNILRRQRRAVVGGTGGGGWRDLDYGNLTNAIRDIVEAMTVDCT
LAVHSGVENAAADFLSNTIKSLHGRGVTTTHHALLSEDHVQSLLIDIRRAVADGHHTSYIV
LSTSALMENLLSAVMSIYIQIFTVLLGLHRDCDFLFFTQIRRSNLMSRNVVYVFLWLRSSVS
RTFKADILEAMRVCVITSPRLGFYQIYYSQASARPGYGSSLKMVNWWSAMDGLVRFPLLP
PPKQVYKNFEGRYFNVPVLHKPPWTFVEYLNDSFRVEGGRDDKLINLLADKLHFQFRYID
PPDRTQGSGLDRGSSMQGVLGLIWQREADWFVGDLSITYERNLVVDFSFLTLVDNEAFLT
HAPGRLNEAFSLIRPFHWSVWPLLLITVIFAGPILYILVDTTDGHPQGKSMLYWKCVWWSV
TVFLQQAAIIPSENNKIRFVAGLLMLSVTYVIGDMYSASLTSILARPPKEPPINTLNELSEAM

RDSGLQLLVEVQSASQAMLENGTGVYYEELSQLVTRQREYLIGSTEKGMQLVRDNKNYAVI
GGRETFYYDIKRFGAQHFHLSEKLNTRYSAIAFQRACPYRDNFDDVLMRLFEGGILSKITE
EEYQKLNDKLMGSEKFDSTSVVIEPVLEGSEPRQEDDDKQLTIAMSMKTLQGAFYVLAIG
SILAGFLLLIEMRSHDKFEKDKRIKRVEAPFVYKRKVPNKFQNRLYDLKE
>ApisIR25a
MLTMYKPIRGITILLWINTLFNIGTSQNVQTVNILFINDRTNEVAEDTLNVALNYIRRNPRVG
LMIDGLYSVKIGGDDASAILETLCVNYNASIRNNKPPHLVIDTTINGVASEAVKSFTAALAL
PTVSASYGQTGDIRQWRNLDGEQQKYLIQISPPADLIPEIVRSIVVAQNITNAGIMFDDTFV
MDHKYKSLLQNIPTRHIIAAIDDTTSIKLHLTRFRDVDIVNFFVLGKLSIIKSVLDHANSNKL
FGRKYAWHVITQDKGSLKCGCSNATILFVKPEPDAGSRERLSNLRTTYGLTSTPELKAAFY
FDFYYRSLLAIRSMMNSGEWPTNVTYTTCDEYNEENPLPRRNVDLRRYLKDMTEPPSYAP
FLIDTNGHSYEEFTMRLEKVTVLNSQSVSAENVGSWKASLNSPIIVKDAANMTHFSAVTV
YRVVTVLQNPFMIQIDDEDGKGVKFKGYCIDLIEEIRKLIGFEYEIYIAPDNNFGNMDENG
QWNGMVKELVEKRADIALGSLSVMAERENVVDFTVPYYDLVGITILMKKPQTPTSLFKFL
TVLENDVWMCILGAYFFTSFLMWVFDRWSPYSYQNNRIKYKDDEEKREFNLKECLWFC
MTSLTPQGGGEAPKNLSGRLVAATWWLFGFIIIASYTANLAAFLTVSRLDTPVESLDDLSK
QYKIQYAPLNGSSAMTYFQRMADIETRFYEIWKDMSLNDSLSEVERAKLAVWDYPVSDK
YTKMWQAMKEAKLPNTLEEAIERVQSSKSSSEGFAYLGDATDIRYQVMIDCHLQMVGDE
FSRKPYAIAVQQGSPLKDQFNNAILLLLNKRKLEKLKETWWNLNPERIQCEKQDNQSDGIS
IHNIGGVFIVIFVGIGLACFTLAFEYWWYKYKKSSRVANTMNPKQITMNRGGEFTYPVVPT
FNANSGMRSRSIIHGFRQSIGQSSPKQK
>ApisIR1
MFVSYDFVVIICLCNVVAQENDNIPLSIVGFFDKENSIEQQTFELSIQKVNIDPKFANVYLK
AEIKIIDSTDTYMTGLKVCETMESGIGAVFGPSHLESSNIVQSLCETMEVPRIDTSWENTPV
ENFNFYFNPHPEPTLLAKGYTAIVHDMDWKSFTLLYQRPECLQRLQDLIQDYSGRTKPND
KQAAAISIIQLPEGNNFRPILKDIKKSLEGHIVLDCDADLILTVFKQAKEVNLLDDYHSFIIT
SLDAHTVDFSSIVQNLRTNITTVRLIDPLSPFVENIVRDLNFVQQRMNLNMEPLKADKLTV
NAILIYDAVNVYAKALKGLGMTNKIITEPLQCTDSPFIPWSNGFKLINFMRVIETEGLTGLL
RFDNKTGHRSYFTLEMVELVDTGFKKIGLWDPERGMTYTRTSLEMLRDLYAGSKNKTFIV
STKIVSKNDCLTYSQRADLGVCDLTITHERRSAVDFTMPFMNLGISILFSKPEEPQTNLFSFT
QPLSFQVWIFTATAYLGLSLVLFFLARITPNEWQNPHPCNPHPVELENSLSLLNCLWFSMGS
ILCQGSDILPRAFPTRLCAAMWWFFALIMTQSYTANWTAFLTSNRMETTIKNVDDLDKKG
GTDGIKYGCVTDQSTASFFQNSDVNLYQKMWSVMELNGDSVMVSDNKQGVDRVKKER
NHYAFFMESSSIEYEVQRNCDLTEVGYWLDNKAYGIAMPFNAPHRTLVNMAVLKLSESGA
LMNLKNRWWSVSDDKRCKDLKKDSAELDVNEVGGMFVILILGCLIAFLFSILEFLWNIRK
VAVEEKLTLWEAFMVELKFVLKCHGTSKPVRHVEDSSSDVTTK
>ApisIR3
MCASVVTLCWSFVMLLLLVGASEDDIVKPSASNKVVKIVALFNENDDSANQLAYNVSAKI
VNIMDTVLPKNKIVPRSEYVVNDMYNVTRVVCDSLKLGIAAFIDGTDDEDIANVVRSISTR
TQIPFIETHWKTFHRPPDPYAINIYPDPSLLAKAIRDIILDMDWNSFTAIYESPESLIRLKALL
MHFDYGHKAPGKSIKIVQIPPTDDFRPLFKELKLSGEKHIILDYEVEHIMHILGQAEEVHFM
GDYQSFVITNLDAHTLSFSEFHKSMANITLIRLINPESQHVRNAVEEIVFNEQKSGRISVLSP
DTMKTKTALLYDAVNFFATSLHGLVATQTMGPMRIACDDIKPWVHGYSLINYMRVMEVN
GLTGKMRFDAETGNRNYFKLDVVRVQESKKHRLGSWDPELNMTLTRSVSEINSEFAQSIT

NKTFIVVGKLVQPYLMRCNATEKGMDKDEECFEGFAYDLVEEMAKYNGFKFKFTTNQDY
GIMNHKTGKWNGMIGELQSMRADLAICDLTITFDRRNAVDFTTPFMTLGISILYAKPEKKK
PQLFWFLNPLSFSVWMYTATAYLGVSLFLFMLARMTPFEWELPHPVKPGDDTLENSMTLL
NCLWFSIGSVLCAGCEVLPKAVSTRLVAGMWWFFALIMTSSYTANLTASITSGRLDTPIKN
VDDLSKDSNIEYGCYEEGSTASFFQKSNLSLYQRMWSVMEASNPTVFTKSNQEGVDRVL
KGKGRYAFLMESSSIEYQTERNCNLMEIGNTLDSKGYGIAMPMNSPYRTLISESVLRLQES
GFMRELKDKWWKVQGDNKCEEEDESDELGFTKIGGVFVVLVLGCLIAFMFSILEFLWNIR
KVAIEEEITPKEALILEWKFAMKCDGGVKPLRRRHHIDTGTNNSDTS
>ApisIR2
MQSAATAVVVLFLLSGFADCAAEDVERITIVGLFPSEDSVEQMAFELAIHKVNLDPTLSND
VKLEGRVEIVDIDDGYQTSKIVCESLESGVGAIFGPAGYESSVIVQSICDSMEIPHIETHWK
MNLRQQPNYYINVYPDPVVLSRGYTAIVRDMDWTSFTLLYQRDEGLARLQHLIQDYSGL
TKLSDTELSAITIIKLSENYDYRPMLKEVKKSLESHIVLDCDTDIILTVLGQAEDVGLMDDY
HSFIITSLDAHTIDYGHLQFKRTNITAVKLIDPSSPTVTNIMADLEFVQQRMNLNMEVFRAE
TITVNALLMFDAVNVYAKALRGIGGTKAIKAEPNSCANRSITGWSSGFSLINFMRVVETDG
LTGKLRFDQDSGYRNYFTLEMVELTNTGFKKIGVWDPQKEMSYTRTRNQMLDDLVNAN
MNKTFIVASKITEPYMMLKEDHKNRVGNDKYEGYVVDLIHMISEEINITYEFKLRNDGNG
KKDKKTGKWDGLIGEVHELRADLAVCDLTITHDRRTAVDFTTPFMNLGISILFSKPKEPET
NLFSFTQPLSFHVWVYTATAYLGLSIILYILARITPNEWQNPHPCASEPEELENSLSLINCLW
FSLGSILCQGSEVLPRAFSTRVCASMWWFFALIVTQSYTANWTAFLTSSRKESAIKRVEDL
DKQSTIKYGCVRGQSTAGFFENSDVNLYQKMYSVMETYGDTVMMYDNKQGVDRVKKE
REAYAFFMESSTIEYEVQRNCDLTEVGTWLDNKAYGIAMPFNAPHRTAVSMALLKLSESG
KLMELKDKWWSVSEDKMCPIPKKDSAELDVNEVGGMFVILILGCMLGFLFSLLEFLWNIR
KVAVAEKLSPWEAFKLELKFVLKCHHSTKPVRHTTYSDEPSSED
>ApisGluRDelta-1-like
MGKFVIFMLFMVFKTAISADYNEMLNVLKTFFVRRHVQTVTAANTCWSFDVNKKLLDG
LSSADISVSFSPPTQQTQNYSTWYRCAFIIDLSCRNSTETLQQISNDRLFNTQNDWILFDEG
SLANDMSLAQFAMRTFQIYLANAYILPDAGVFLFLETYNSDIWEIWSGFRASKLDTIRVFE
YGRASSNQLTISESHDERRNFRGITLKSTTVIIDRDHFFGFDKKISSDLDVFAHMHYEMIVT
LTNQLNFKIDLTIDNDYGWSLGNGSFGGVTGLLQREEIDFSATGVFIRPDRMSVIDFTVGTI
ALRTTAIFRQPSLSSVHNILLLPFTFDVWLGICATLCAFVLTLVFLMRVNNYFAEKKYDTLN
VLEIVTLVHGAICQQGSNNSTLTLGSIRVVISILFFTSLFIYTSYSASIAALVQSNSNSIKSIKNI
VESPMTFSAQISQYGKHYFEETEDLELRKLYKTKIVPSGNKSFVRAAEGMERIRTEFHGFQ
VEVMSAYKIISKEWREEEKCGLGEIQLFKIPLLSIALVKKSGHKDIFKQKLIQQMEVGLNKR
IASQWLPPKPSCGSSGRAKQYVSVSVKETYVTLAIFGFGVCISLLIFILEVLQHAWTNRGSK
KNLWK
>MperGluRkainate-4-like
MTSAQFALRTFQIYLANAYVLPDAGVFLFLETNNDVWEIWSGFRASKLDSIRVFEYGTASS
NRLTVVRELHDERRNFRGITLKSTTVIIDRDHFFGFDKKISSDLDVFAHMHYEMIVTLTYQ
LNFKIDLTIDNDYGWSLGNGSFGGLTGLLQREAVDFSATGVFIRPDRMSVIDFTVGTVALR
TTAIFRQPSLSSVHNILLLPFTLGVWLGVCATLCVFVLTLVFLMRVNNYIVEQNYGTLNIPEI
VTLVHGAICQQGFNSTSTLGSIRVVVTILFLTSLFIYTSYSACIAALVQSNSNSIKSIQNIAESP
MTFSAQISQYGKVYFEETEDAELKKLYRTKMVPSGKKAFVKAAEGMERIRTEFHGFQVE

VMSAYKIIGKEWREEEKCGLGEIQLFKIPLLSIALVKKSGHKDIFKQKLIQQMEVGLSKRIA
SQWIPPKPSCGSSKQYVSVSVKDTYLTLAIFGFGVCISLLIFILEVLHHAWTNRGSKKNAWE
>DnoxIR2
MKIQHRNSLILELLICWSLVRTLKSNKIKIGALFTHDQINSSLEIAFKFAVDQINRDAHLLPH
TQVSYDIQYVPHDDSFYTIKTACSQLDLGIAALFGPSNSYLASHLQSMCDTLDLPHIEARL
DLEMDPKRLSINLYPKQSLLNQAYSDVISFLNWTKMAIIYEHDYDLFRMKDFMKTVPDRK
TVEVYLRHATPDTYRSVLNEIKNMEIYNMLVDTKSEHVDHFLRGILQLQMNDYKFHYLFT
SFDMEMFDLEDFKYNFVNLTTFRMVDIEDVGVREVLKHMEQYSTEKNQSINKFMLTQCE
PALIYDSVQVLAVGLGALQQNNILEYPNISCKDEXPWSNGSSLINYINSVEIKGLTGPIQFK
NGQRTDFKLDLMKLKQHALAKVGEWTPGLGVNITDMSAFHDTAPYNITLVVVTIVEFPY
VMMHLEKNYTGNARFYGFCVDLLEIIAKQVGFDYILDLVPDNKYGAQDPVTLEWNGIVE
QLIKHKADLAVGSMTINYVRESVIDFTKPFMNLGISILYKVPSNQPAQFFSFMNPLALEVWI
YWLIAYVVVSVTLYIVAKFSPYEWEDQTSGKLFSSPNLATNVPQINRFTLSNSFWFTIGSLM
QQGSDISPKAISTKIIGCVWWSFTLIIVSSYTANLAAFLTVERMVSPIENAEDLAAQTNIAYG
TLDSGTTMTFFRDSMIETYKKMWAYMQKKKPSVFVTSYDEGIERVLQGNFAFLMESTML
DYVVQRNCNLTQINGLLDSKGYGIATPMGSPWKDQISLSILDLQEKGEIQMLYNKWWKPP
NDMCVPEGSGAGAKTNTLDFKNICGVFAVLLVGLIISIMVSMFEFYHYRKKIKQIEQRIGPQ
RSTWRDLVDELWFVLKCQGSRQRTVLTHRCEQCHPKVRTAERRSRRDFIEASRL
>RmaiIR2-like
MKMRHWSSLILEVLICWSLVRTLRSNKIKIGALFTHDQINSSLEIAFKFAVDQINRDAHLLP
HTQVSYDIQYVPYDDSFYTIKTACNQLDLGIAALFGPSNSYLASHLQSMCDTLDLPHIEAR
LDLEMDPKRLSINLYPKQSLLNQAYNDVISFLNWTKMAIIYEHDYDLFRMKDFMKTVPDR
KTVEVYLRHATPETYRSVLNEIKNMEIYNMLVDTKSEHVDHFLRGILQLQMNDYKFHYLF
TSFDMEMFDLEDFKYNFVNLTTFRMVDIEDVGVREVLKHMEQYSTEKNQSINKFMLTQC
EPALMYDSVQVLAVGLGALQQHNILDYPNISCKDEVPWSNGSSLINYINSVEIKGLTGPIQF
KNGQRTDFKLDLMKLKQHALAKVGEWTPGLGVNITDMSAFHDTAPYNITLVVVTIVEFP
YVMMHLEKNYTGNARFYGFCVDLLEIIAKQVGFDYILDLVPDNKYGAQDPVTLEWNGIV
EQLIKHKADLAVGSMTINYVRESVIDFTKPFMNLGISILYKVPSNQPAQFFSFMNPLALEV
WIYWLLAYVVVSVTLYIVAKFSPYEWEDQNSGKLFSSPVLETNMSQLNRFTLSNSFWFTIG
SLMQQGSDISPKAISTKIIGCVWWSFTLIIVSSYTANLAAFLTVDRMVSPIENAEDLAAQTNI
AYGTLDSGTTMTFFRDSMIETYKKMWAYMQKKKPSVFVTSYDEGIERVLQGNFAFLMES
TMLDYAVQRNCNLTQINGLLDSKGYGIATPMGSPWKDQISLSILDLQEKGEIQMLYNKWW
KPPNDMCVPEESGTGTKTNTLDFKNICGVFAVLLVGLIISIMVSIFEFYHYRKKIKQIEQQV
GPQRSTWRDLVDELWFVLKCQGSRQRTVLMHRCEQCHPKVRMTERRSRRDFIEASRL
>RmaiIR1.X1
MKLEIVVWIIHFVSRVYTLPDTIRIGGLFHPNDINQENVFKHAIHDVNANRLILSRSNLSGQ
VEKVSPQDSFHASKRVCSLLRLGVAAVFGPQSAQISSHVQSICDTMEIPHLETRWDYKLRR
ESCLVNLYPHPTVLSKAYLDLVKKWGWKSFTIIYESNEGLVRLQELLKARNGAFSAYPITIR
QLGSSRDHRPLLKQIKNSAESHIVLDCSTEKIYDVLKQAQQIGMMSDYHSYLITSLDLHTI
DLDEFKYGGTNITGFRLVNPDTPVVQKVLKQWGENFTVMSTETALIYDAVHLFARALHDL
DSSQKIDIKPLSCDASDTWSHGYSLINYMKIVEISGLTGVIKFDNQGFRTDFELDVVEVNKE
GLSKIGTWNSSQGINFTRSFVEAYSSIVDNLHNKTLVVTLILSSPYTMRRESSLKLVGNDQF
EGYAIDLIYEISKLLGFNYTLKLVPDGRYGSYNEDTKEWDGMMGELLQQRADLVVADLTI
TYDREQAVDFTMPFMNLGISILYRKPIKQPPNLFSFLSPLSLDVWIYMATAYLGVSVLLYIL

ARFSPYEWENPHPCNSEAPDVFENKFSLNNSLWFTIGSLMQQGSDMAPKAVSTRIVAGM
WWFFTLIMISSYTANLAAFLTVERMDSPIESAEDLAKQTKIKYGALRGGSTTAFFRDSNFIT
YQRMWSFMESSRPSVFMSSNNEGVERVVKGKGNYAFLMESTSIEYVIERNCELTQVGGLL
DSKGYGIAMPPNSPYRTAISGAVLKLQEIGKLHKLKTKWWKEKRGGGACRDDTSKSNSA
ANELGLANVGGVFVVLMGGMGVACVVAVFEFVWKSRKIAVEERIIKYHTKYRK
>RmaiIR1.X2
MKLEIVVWIIHFVSRVYTLPDTIRIGGLFHPNDINQENVFKHAIHDVNANRLILSRSNLSGQ
VEKVSPQDSFHASKRVCSLLRLGVAAVFGPQSAQISSHVQSICDTMEIPHLETRWDYKLRR
ESCLVNLYPHPTVLSKAYLDLVKKWGWKSFTIIYESNEGLVRLQELLKARNGAFSAYPITIR
QLGSSRDHRPLLKQIKNSAESHIVLDCSTEKIYDVLKQAQQIGMMSDYHSYLITSLDLHTI
DLDEFKYGGTNITGFRLVNPDTPVVQKVLKQWGENFTVMSTETALIYDAVHLFARALHDL
DSSQKIDIKPLSCDASDTWSHGYSLINYMKIVEISGLTGVIKFDNQGFRTDFELDVVEVNKE
GLSKIGTWNSSQGINFTRSFVEAYSSIVDNLHNKTLVVTLILSSPYTMRRESSLKLVGNDQF
EGYAIDLIYEISKLLGFNYTLKLVPDGRYGSYNEDTKEWDGMMGELLQQRADLVVADLTI
TYDREQAVDFTMPFMNLGISILYRKPIKQPPNLFSFLSPLSLDVWIYMATAYLGVSVLLYIL
ARFTPYEWYNPHPCNPDPDNLENQFSLMNCMWFAFGSLMQQGCDILPKAVSTRIVAGMW
WFFTLIMISSYTANLAAFLTVERMDSPIESAEDLAKQTKIKYGALRGGSTTAFFRDSNFITY
QRMWSFMESSRPSVFMSSNNEGVERVVKGKGNYAFLMESTSIEYVIERNCELTQVGGLLD
SKGYGIAMPPNSPYRTAISGAVLKLQEIGKLHKLKTKWWKEKRGGGACRDDTSKSNSAA
NELGLANVGGVFVVLMGGMGVACVVAVFEFVWKSRKIAVEERV
>RmaiIR3-like.X1
MNAVVDLLVPLVLILAGGKRCAGDTTITLGILYNEENSMLETAFKSSVDVAKAKMIDSGV
QLDVVSKIVPLYDSFETQHHVCEMLVDGVSGMFGPSAGDTAPIVQSICDYKEIPHIQTRWD
VNQKRGSCQINLYPHPNTLAEALIDIIVAANWESFTIIYENNDSLMQITNILKSPPTNYPIRIR
QLSSGPNYRKELREIKDSGETKILLDCSFSILTDVLLQAQQVGLMGSEHNFIIASLDMHTLD
LDPFRYSGTKITGMRLVKPLDIEFQNIVSQWTDNLSPLEPDDKIVLPETIQLESALIYDAVQL
FTTSIYNLSKEFEISETPTPCNSSLSWKHGFTLINYMKMAKDFKGLTGKIKFDQEGFRTDIE
LELVDLTQDGLRVTGTWNTKTGINVSATPKPEIVSSGKDSDLRNMSFVVITALTQPYGMLK
LSSNTLKGNDRYEGFGIDLIKELSEMSGFNYTFIIQEDSNSGYKDDKTKKWSGMIGEVING
QADLAIADISITRQREHDVDFTSPFMNLGISILYKKSTKSSPSLFSFLAPFSSFVWLWVITAY
CGVSVLLFIMARISPYEWTNPYPCIEEPEYLENQFSLSNAFWFTIGSLMQQGSDIAPIAVSTR
LVAGIWWFFTLIMVSSYTANLAAFLTVESVSEPFKDVEDLVNNQNIISFGLKKKGSTEEYFK
ESTNPTYKKLFDILQKNPAWYTANNDEGVEKVLKENYAFFMESTSIEYMVERNCKLAQIG
GLLDNKGYGIVMKKNASFRNVLSANILSLQEKGKLTALKNKWWKEKRGGGACQDTDNN
EASELSMKNVGGVFIVLCSGVAVAAVLAALEMFWTLWKTTSKEKVSFKSEFKDELKFIAK
CRGSTKPARKNQNSSADNSNPTGSNQYMNSDRHYDYES
>RmaiIR3-like.X2
MNAVVDLLVPLVLILAGGKRCAGDTTITLGILYNEENSMLETAFKSSVDVAKAKMIDSGV
QLDVVSKIVPLYDSFETQHHVCEMLVDGVSGMFGPSAGDTAPIVQSICDYKEIPHIQTRWD
VNQKRGSCQINLYPHPNTLAEALIDIIVAANWESFTIIYENNDSLMQITNILKSPPTNYPIRIR
QLSSGPNYRKELREIKDSGETKILLDCSFSILTDVLLQAQQVGLMGSEHNFIIASLDMHTLD
LDPFRYSGTKITGMRLVKPLDIEFQNIVSQWTDNLSPLEPDDKIVLPETIQLESALIYDAVQL
FTTSIYNLSKEFEISETPTPCNSSLSWKHGFTLINYMKMAKDFKGLTGKIKFDQEGFRTDIE
LELVDLTQDGLRVTGTWNTKTGINVSATPKPEIVSSGKDSDLRNMSFVVITALTQPYGMLK

LSSNTLKGNDRYEGFGIDLIKELSEMSGFNYTFIIQEDSNSGYKDDKTKKWSGMIGEVING
QADLAIADISITRQREHDVDFTSPFMNLGISILYKKSTKSSPSLFSFLAPFSSFVWLWVITAY
CGVSVLLFIMARISPYEWTNPYPCIEEPEYLENQFSLSNAFWFTIGSLMQQGSDIAPIAVSTR
LVAGIWWFFTLIMVSSYTANLAAFLTVESVSEPFKDVEDLVNNQNIISFGLKKKGSTEEYFK
ESTNPTYKKLFDILQKNPAWYTANNDEGVEKVLKENYAFFMESTSIEYMVERNCKLAQIG
GLLDNKGYGIVMKKSILSNIVREFSKCSERKHIEPAREGQTDSVKEQMVERETRGRSVSGH
RQ
>RmaiIR40a-like.X1
MSGRGRKKNENRTRFTFRRGMAAAERCVLALTVFAVVLATMVIPERDDHSNIWRRQRRA
VVGDGGWRNLDYGNLTNAIRDIVEAMTVDCTLAVHSGVENAAADFLSNTIKSLHGRGVT
TTHHALLTEDHVQSLLVNIRRAVADGHHTSYIVLSTSALMENLLSAIRRSNLMSRNVVYVF
LWLRSPVSRTFKADILEAMRVCVITSPRPGFYQIYYSQATARPGYGSTLKMVNWWSAMD
GLVRFPLLPPPKKVYKNFEGRYFNVPVLHKPPWTFVEYLNDSFRVEGGRDDKLINLLADK
LHFQFRYIDPPDRTQGSGLDHGSSMQGVLGLIWQREADWFVGDLSITYERNLVVDFSFLTL
VDNEAFLTHAPGRLNEAFSLIRPFHWSVWPLILITVIFSGPILYILVDTTDGHPQGKSMLYW
KCVWWSVTVFLQQAAIIPSENNKIRFVAGLLMLSVTYVIGDMYSASLTSILARPPKEPPINT
LNELSEAMRDSGLQLLVEVQSASQAMLENGTGVYEELSQLVTRQREYLIGSTEKGMQLV
RDNKNYAVIGGRETFYYDIKRFGAQHFHLSEKLNTRYSAIAFQRACPYRDNFDDVLMRLF
EGGILSKITEEEYQKLNDKLMGSEKFDAASVVIEPVLEGSEPQQEDDDKQLTIAMSMKTL
QGAFYVLAIGSILAGFLLLIEMRSHDKWKNDKRIKRIKAPFVYKQKAPIKFQNRLYDLKE
>MsacIR40a.X1
MAAAERCVLALTVFVVLATMERLPERDDHYSNIWRRQRRAVVGGDGGGWRDLDYGNLT
NAIRDIVEAMTVDCTLAVHSGVENAAADFLSNTIKSLHGRGVTTTHHALLTEDHVQSLLI
NIRRAVTDGHHTSYIVLSTSTLMENLLSAIRRSNLMSRNVVYVFLWLRSSVSRTFKADILE
AMRVCVITSPRPGFYQIYYSQASARPGYGSTLKMVNWWSAMDGLVRFPLLPPPKQVYKN
FEGRYFNVPVLHKPPWTFVEYLNDSFRVEGGRDDKLINLLADKLHFQFRYIDPPDRTQGS
GLDHGSSMQGVLGLIWQREADWFVGDLSITYERNLVVDFSFLTLVDNEAFLTHAPGRLNE
AFSLIRPFHWSVWPLLLITVIFSGPILYILVDTTDGHPQGKSILYWKCVWWSVTVFLQQAAI
IPSENNKIRFVAGLLMLSVTYVIGDMYSASLTSILARPPKEPPINTLNELSEAMRDSGLQLLV
EVQSASQAMLENGTGVYEELSQLVTRQREYLIGSTEKGMQLVRDNKNYAVIGGRETFYY
DIKRFGAQHFHLSEKLNTRYSAIAFQRACPYRNNFDDVLMRLFEGGILSKITEEEYQKLND
KLMGSEKFDATPVVIEPVLEGSEPRQEDDDKQLTIAMSMKTLQGAFYVLAIGSILAGFLLLI
EMRSHDKLENNKRIKHIKAPFVYKQKAPIKFQNRLYDLKE
>AlinIR1
MIREWMNMFTAAAFLLACAHVASALPEVIKIGAVFEEGDEWSRYAFEAAIKIINEDESTLP
GIKLEAAIAEEPIPQYDIVGVENHVCDMMKAGVVGIVGPHSTEMSNHVQSLCDTMEIPHIS
ARWDGQQRRSSCLVNLYPHPSVLAQVAADMVRTWDWKGFTVLYDDFNALRKIGELVKV
ADDKGLIVSVRQIKGTEEEESNYRYVLQQVKHSGETNIVVEVSRERLFDVMMQAQQVGL
VGGEYSYIITSLDFQSIDLEPFKWAGTNITGIRIVNPDQPHFKEVMKLIMEMKTQGEGGEG
AGEEGGGEEGEKEEVGENNGDEAEEGADAEDELGEGEEEGGGKEEENKRRKRRTQQDD
EENEVIGGEEEGAEGGEEEGGGEEEVGENGEGGGEGPDDGAEGNQEEENGEGGEEEAGG
GAPEEAPAEEEEEYEIPPIEALLIYDAVNLVAEALHNLDIVEPREIDCRQNNAWESGYSVINF
VKMSEQAGLTGLVKFDNEGFRSEVVLEIVELVQEGLRVKGNWTKQDGVTIHYIGGEAGPT
DIGDDLRNTTFVVLIALTHPYGMLKEDSRQLMGNDRFEGFGIDLIHELSMMSGFNYTFRV

QEDKSSGSPKTLENGTRVWSGMIGEVLAGRADLAIADMTITRERERDVDFTMPFMNLGIS
ILYRKPMAMPPSLFSFLSPFSYEVWGYILSAYLGVSFLLFIMARISPYEWTNPYPCIEEPTEL
ETQFSLSNSLWFTTGSILQQGSDVAPISVSTRMVAAIWWFFTLIMVSSYTANLAAFLTIEQKI
EPFTDVEGLANSEGIKYGAKKGGATANFFRDSNEPIFQKMWTFMEANPDVMPTSNEAGV
ARVQENTDYAFLMESASIEYEQERKCELTMVGDLLDSKGYGIAMRQNSSYRNVLSRNVIK
LQEKGKLTQLRDKWWKEKRGGGACNAEEEGGEASELGLDNVGGVFVVLLGGCILSVFL
AFGELLCDIYGREDKVSFKDELIEEIKFIARCHGTVKPVRKGEPANSSSSSSKSGSRSGSKS
GSKTRSSRTRTRSGSGGSPSVERHRFSSRLYSDPLSLD
>AlinIR8a
MRTCLWFHVVLFVAHEVAGQGVKLLVVKDNNAGIWDSVSTSFFEQLPVTVDKEDTNSTI
NDLCEVLKEGVWGVLDLTWSGLDEIKAVCNTWGLPYVRLEYGITQYLRGADKSLATIRK
APDAALIFQTEEQLDQSLFYLIRESSMRVILFKGLSDKEAETLTTMRPTPNFNIIFADTPSMN
VMFLKAVERNLVRYDDRWILVFLDNEHNSFDRKTLVKRVTLATPTIDANAAANFAENVAE
TLEEVAKTSGIDLSPVPAQCEGSSTAAKDLTVFQEKLSEIVEKKPWLDWRQQESTMALHL
DMDWTAESSKGEKLFIGSWNSKKGLTIAGNVTKIPRFFRVATGYMVPFAYPVIDPSTGAPK
LDDKGNEVWEGYCIDLINRLAEDMDFDYELTTSYNFGRKLPNGSWDGLIGDLASGRVDII
VAALTMTSEREEVIDFVAPYFEQTGFSIVIRKPLRKTSLFKFMTVLRVEVWFSILAALCLTAF
MIWFLDKYSPYSARNNKDKYPYPTREFTLRESFWFAVTSFTPQGGGEAPKSLSARTLVAAY
WLFVVLMLATFTANLAAFLTVERMQSPVQSLKQLARQSRINYTVVQDSDAHSYFRNMKF
AEETLYRVWKEITLNASANQSQYRVWDYPIKEQYGHILISMEKTGTVNSTEEGFQKVREN
EDAEFALIHDALEIKYEVYRDCNLTEIGEPFAEQPYSIAVQQGSHLNEEISRRILDLQKDRYF
ESLSGKYWNSTMKGKCDSSDEDEGITLESLGGVFIATLFGLVLAMLTLGIEIVYERKAKKN
VIKVKSAKPEKSEKSEKKEKMMNNPFFNDDKLFSREFGSFPKKPSKLLAPKPKVSFITVFP
RDQLY
>AlinIR21a
MKLEVLFVTTLVHLTHSVKITKLLNSMAFADLETISCMAPDGIPYMVPLINSIAKRYLKDH
ATVILYDDYFYYHPRLKNMIDILISNYAYPLRHGLVNTTMAKPTVPAGILEARENEQMAFI
VFTKESEIGAEAIREFTGHNTMTLLIAQTSVYHVKLFLQTKLAADITNLLVFVDPMIKIDHF
VQKTARVLKECDILIFSHKVITDSLGISMPVIVTAWRRNHLTRQVQLFPPKYKRGLGGLHL
VASASEIPPFVFRKHGHDSGAGYTITKWDGIEVHLLYMLSQMLNFTVEYKEPEFNEEEDVA
QTVIKDLHTKKTTLAIGGVYLTPERIGGLTFSFPHTQDCASFISLASTALPKYRAIMGPFLW
DVWLALTAVYLLAMFPIAFSVWHSIKPLLNDIREVENMFWYVFGTFTNCFTFTGKNSWSK
ADKTATKFFIGTYWIFTIIITACYTGSIVAFITLPTYPETIDSSKQLLEEDYKISLLGSGGWEGL
FNDTEDPVASKLYESVERVPNLYSGLRNVTRNVHSWRQSAFLGSRRLLEYTVKTNFTPDE
DSKRLMFHLSDECFVPLFVSIVMDKRTNYLEEFNNALERIIQSGFMTKIVREVEWQEYRSA
SGKLLTMHKGLKGAPEDRELNLDDTQGMFLLLGAGFGIGLLVLIIEISVWSSEQRKNRQFG
ELTLKQRAINKLKEHWETLYACLLAPANSGIIYFRERRVSSAFGEYVTQPYASWSITSIPSPT
VEPPSPSPPNGEISSAPTVGNAASLSMDQLSFPPEKPIRMMSF
>AlinIR25a
MPSFTARLPGTTSVAKVFTVFMFYVSLLQKVHSQSATSINVMFVTEDRNDIARLAFDVVS
DYVKRNSKLGIEMEVFRVTESGSDAKFLLENLCETFNASAKAGKPPHIILDTSVVGVTSEA
VKTFSRALGIPTLSASYGQEGDLRQWRALEVEIAKYLLQINPPADIIPEVVRSIVILQNISSA
GIVFDDSFVMDHKYKSLLLNVPARHIMGRVRNIQEIRNQLTRFKELDIVNFFILGSLSTIRN
VLNEANGMKFFDRKYAWHAITQDKGQLKCDCSNATILHIKPEPDPGSKERLDNLRTSYNL

VEEPEITSVFYFDFFLRGLLAAKALIEKAPWPKDYNKTSCDNYDENHDFIRKDLDLRSSLR
DVKEAYSYAPFLISTNGKSFMEFNMKIEKVVIVNSIAESAEAIGTWKAGLSNQIQTKDIAS
MRNFSAVTVYRVVTVKQKPFVIETFENGKPKYSGYCIDLLEDIRSFVHFEYDIYVAPDNAY
GNMDPSGNWNGMIKELIEKRAEIGLGALSVMAERENVVDFTVPYYDLVGITIMMKKQTT
QTSLFKFLTVLENEVWLCILASYFFTSLLMWVFDRWSPYSYQNNREKYKNDEEKREFNLK
ECLWFCMTSLTPQGGGEAPKNLSGRLVAATWWLFGFIIIASYTANLAAFLTVSRLDTPIESL
DDLAKQYKIRYAPINGSEAMTYFQRMADIEERFYEIWKDMSLNDSLSEVERAKLAVWDY
PVSDKYTKMWQAMKEAGLPATLEEALDRVRKSQTTSEGFAFLGDATDIKYLVLSSCDFQI
VGDEFSRKPYAIAVQQGSPLKDQFNNAILQLLNKRKLEKLKEKWWTENEDRMQCEKQEE
QSDGISIHNIGGVFIVIFVGIGLACITLGLEYWWYKYKKPASPKQVGPMAQIISTNATNKQL
SVTGLMDYNTREPRARYPIRRTAVNATQDYSRPTFPAQQERLSHW
>AlinIR75d
MGALLPYSLITQYFINIHVSSIIVVSCCTTSQTAQLLRHLSQRGITASWAVDNLSPLEVRRSG
IVLDLSCNQSKEILHDMSSRKMFGLEMEWLLMSEGSAPEEAELPDLYILPGSSVTLSVTSPS
SISFYDTYRITRRLPYKFTLLGAVARDEDVLPQWKRPSRVNYEQNLLTTVSVIHSLDIRKLT
DPDVAEEDRWPAIHFPVVVNVAYQLNFKFDLRLESVHGWKFPNGSFEGMIGVMEREEVD
FGASGVIMREDRRKHVDYTVDYFEFKTGIIFKQPSLSSVSNIYLLPFSRHVWAACGGLLLF
VLIILCIAVSSGDAQTFTPPATFLDMVNIVLGFVCQQGSYLAPVTISGRIVVFVSSLAALFLY
TSYSANIVALLQSTSSVLKTLKDLTNSHLGLKVQINEYHLGYFLEAVDEDVITLYNKKVKN
QPETFVNGTRGVEFMRTGDFAFCVEFDLAYKQISKTFQEEEKCGLGEMHLFFVPRLSIPVI
KRSGHREHFTQTIIWQWESGMLDRISRIWLARRPRCESTGGGYLRVGLKDFNPALKVILVG
IIISIWFFLCELITDRGFKAYYRKIKHNQEKIMGDDGHLIADLCFPSWKMLFKNKRF
>AlinIR76b
MSPFVHLMLVAMCANYQTNLLATDNQNFTCILKSEEQIKKEVYKGKVIKILTFDEMPLSG
ARKDGKGGMIGEGVAFELVETLKEKFGFDYTVERMAPIVGDESHGALGKLVSREIDMVA
AFIPVLPDAHEFVKFGKDLSQAAYYVMLKRPADSNSGSGLLAPFDTVVWLLILVSLAVVG
PVFYGVMWLRDRLCPGDIDQVYPLSTCVWFVYGALMKQGSTLNPLADSARMVFATWWI
FILILTAFYTANLTAFLTLSISTLPIKEIDDVAKDNRHWFALQGGPIEHAIKDKEDEKLRKLRD
SAASGRATFLETKQESIILQKITNDWYYLDDSYSLTRMMYDDYNRKSDMNAESSLRCAYV
LTEKPFLVRSLAFAYGKDSPLPDLFNPILERFIESGILQHKLNLDLPDAVICPKDLGNKERKL
RNADLWTTYLVVFSGVSVAFMIFLIEIIWRFYRKVKGSNQGVFNKQQMFQSRLNTDKLLA
MRDQVQTKINGRDYYMVTNKGGNSHFIPLRTPSALLFQYG
>AlucIR21a
MRIEFFLLVVIANLAVPTKISKLLESLAVVQHESIACMPPDGVPYMVPLLNAIARRYLKDH
VTVVLYDDYFYFHPRLKSMLDHILLNYAYPLRHGRINTTMAKPKVPPGILEARENEQMAFI
VFTKETEIGAESIREYTSRNTMTLLIAPTSVYMVRQFLGTKLAGDITNLLVIVDPMIRVKLL
SQKVGQVFKECDILIYSHEFASDSLGSTTPVIVTAWRRTQFTRQVQLFPSKFKQGLGGIHLT
VAASEIAPFVFRKRGQESGAGYTITKWDGIEIRLLNIVSQMLNFTVEYKEPELIEEEDVAQA
VIKEVLAKKANLAVGGVYLTPERINGLLFSIPHTRDCASYISLSSTALPKYRAIMGPFLWDV
WLALIAVYLFAMFPIAFSVWHSLKPLLDDLWEMENMFWYVFGTFTNCFTFSGENSWGKS
ERTATKLFIGTYWVFTIIITACYTGSIVAFITLPVFPKTIDTSKQLLEEDYKISVLSSGGWERL
YNETDDPVAVKLYKSVNLVPDLSAGLLNVTRNVHSWRQSAFLGSRRLLEHTVRTNFTPDE
DSKRLLFHLSDECFVPLLVSIVLQKRTHYLEEINGALERALQAGFMTKVTQELEWEEYRS
ATGKLLKVHKGLKGAPEDRELNLDDTQGMFLLLGAGFAIGLFVLIIEVSVWSTGQVKHRQ

FGDLTLKQRAFNKLKEHGEALYNCLLAPAKSGIIYFRERRVSSAFGEYVTRPYTPWPRSSIP
SPTVQSPSPPPPNGMVSSAPPIDVGADSLSMDQLPLPRERPVRLMSF
>AlucIR25a
MLSPAATTTGIATLTFFILWVCFQTAHSQSATTINVMLIKEERNDIARMAFEVTQDYIKRNS
KLGLEMNVYKVTESGSDAKLLLENLCETFNASAQAGKPPHMILDTSVVGVTSEAVKTFSK
ALGIPTLSASYGQEGDLRQWRALEGEIAKYLLQINPPADIIPEIVRSIVKLQNISSAGIVFDES
FVMDHKYKSLLLNVPTRHIMGQVRSIQEIRNQLTRFKELDIVNFFILGSLSTIKNVLTEANG
MKFFDRKYAWHAITQDKGPLKCDCSNATILHVKPEPDPGSKERLDNLKTSYNLMEEPEITS
VFYFDFFLRGLLAAKSLLEKANWPKDYNKTSCDDYDENHDFVRKDLDLRSSLREVKEPY
SYAPFLIESNGKSFMEFNMKIEKVVIVNSIAESAEAIGTWKAGLNNPIQTKDQASMRNFSA
VTVYRVVTVKQKPFVIETFENGKPKYSGYCIDLLEEIRSFVHFEYDIYVAPDNAYGNMDAS
GNWNGMIKELIEKRADIGLGALSVMAERENVVDFTVPYYDLVGITIMMKKQTTQTSLFKF
LTVLENEVWLCILASYFFTSFLMWVFDRWSPYSYQNNREKYKNDEEKREFNLKECLWFC
MTSLTPQGGGEAPKNLSGRLVAATWWLFGFIIIASYTANLAAFLTVSRLDTPIESLDDLAKQ
YKIRYAPVNGSEAMTYFQRMADIEERFYEIWKDMSLNDSLTEVERAKLAVWDYPVSDKY
TKMWQAMKEAGLPATLEEALERVRSSQSTSEGFAFLGDATDIKYLEMTSCDFQIVGDEFS
RKPYAIAVQQGSPLKDQFNNAILQLLNKRKLEKLKEKWWTENNDRKECDKQEEQSDGISI
HNIGGVFIVIFVGIGLACITLGMEYWWYKYKKPVSPKQVGQPAQTIPGNATNKQLSVTGL
MDYGSREPRARYPIRRANANGPLDYDRPSFPAQQERLSHW
>AlucIR41a.1
MEGIRIVMILEVALLFSGSTKALNFTQNTESIEDALTTLAEEISTKYYVQISKGCIVLMTRPG
FMGTFSVPGAAIIRLVSDNFCNDTDALNAMVTVFDEMCYNFIVQISEVHCFFKVWLDAQ
WKTIQRWHPEILFLPAYPEQTETADDVFTLKESDYSSSILAINIDETNSDWPFSIYTSNFYER
VDKPGRNPKIFLDQWSSYASFRHDANLMIDFIQNLQNKTLKIMTFDYDPYTHFEPLDGTEI
KLIQEFCKKHNCSLVAVDDGHYWGDIFENGTSDGLAGMVYDGRADFGAAAVYLWLPYF
YFVDYSTSYLYSASTLLVPKPHPVSGWRTPFLPFDMLTWISYGLSVLMAAVFMYVITYLTV
KYTRFAEAVQKRRMFLDKLDCIFRALGLAVLQQPSTPLVPHTPIRHLFTSFEFLFLIASSIYA
AELASYLTVPRYEKPIDTLIEYSDSGMIWIGEHESWTYSLRGMTDPEIVNIVNNYLICSHEK
LMKLAPTGEYGLIVERLPGGHYTEQDHVTDDIVAQSHMMAENLFGSPPVIAVRKGSPYRK
YFNKVISNVLCGGLYLYWEGEMSRKYLHSRRQLALREADHPHYKDIPKNLEISHIQGGLF
LYAIGATISIFVFMFELFFHYRKMSRKFIRKFT
>MsanIR25a
GDTMFVTRIHIFVLWLTVYTNGEIYKIGVLTQSEDDKNFVQLKSIMTNYNISIEPILNDPSY
NITNKFCSVASNNTLATIDILIPSCIPCWKISNANSMAYFRTDFSYIQPAIQLIESYVTWLNIT
KEITFVFTNQEDADQAVTYLTSGKSSLRAIVLSHLTSNEIDRLKNTKIGIRHVALIGNNLDQ
YVQKINQEKLIKLDESWIIVTNDTTKLKFISTVTLMKFTSWENGYTNKMTRAKTLFNFIFY
FLDHASRDHPKLNCDSISDVLMLEKRKEIEKLLNSYENKNEFQYDIDTNLMRYNEQAMIF
KVIPDSVPNHLGTWTINGGLEMKYDASTVVSGRRFFRIGTAKSIPWTFMEDVWKGYCIDL
IEKLSKEMNFKYELVVKDKFGSLDPVTNQWNGLIGGLVEGELDIVIAALTMTSEREEVIDFI
APYFEQTGISIVIRKPSRKTSLFKFMTVLKPEVWLSIVAALAMTAVMIWILDKYSPYSAQN
NKTKYEQFRHFTLVESFWFALTSFTPQGGGETPKAISGRVLVAAYWVFVVLMLATFTANLA
AFLTVERMQTPVQSLQQLARQSRINYSVIDGSDAHNFFRNMKMAEDILYNVWKEIALNQT
NNRKDFRVWDYPIKEEYGQILAAIERTGTVPNRSVGYQMVLDNEQGEFALIHDSSDIEYE
VYNNCNLTEVGEIFAERPYSIAVQQGSLIQEEISRKILDLQKDRFFELLNAKYWNASKVSM

CPNADDSEGITLESLGGVFIATLVGLLIALITLAFEVVYFKHKRTKVAEVSVVNNTVHKDK
LMYGHELFMTLGRNSNSNDQTPWANKIKLDSTTGRLNNALFF
>MsanIR40a
DDDRYARRRRVVERETAKEERSERKPQAHRRGMATAERWVPALMLFTALATAAIPGQDD
HCRHHSNIWRRQRRAVVGGGGGWRDLDYGNLTNAIRDIVEAMTTDCTLAVHSGVDNAA
ADFLSNTIKSLHGRGVTTTHHALLSEDHVQSLLIDIRRAVADGHHTSYIVLSTSTLMENLLS
AIRRSNLMSRNVVYVFLWLRSSVSRTFKADILEAMRVCVITSPRPGFYQIYYSQASARPGY
GSSLKMVNWWSAMDGLVRFPLLPPPKQVYKNFEGRYFNVPVLHKPPWTFVEYLNDSFR
VEGGRDDKLINLLADKLHFQFRYIDPPDRTQGSGLDLGSSMQGVLGLIWQREADWFVGD
LSITYERNLVVDFSFLTLVDNEAFLTHAPGRLNEAFSLIRPFHWSVWPLLLITVIFVGPILYIL
VDTTDGHPQGKSMLYWKCVWWSVTVFLQQAAIIPSENNKIRFVAGLFMLSVTYVIGDMY
SASLTSILARPPKEPPINTLKELSEAMRDSGLQLLVEVQSASQAMLENGTGVYEELSQLVT
RQREYLIGSTEKGMQLVRDNKNYAVIGGRETFYYDIKRFGAQHFHLSEKLNTRYSAIAFQR
ACPYRDNFDDVLMRLFEGGILSKITEEEYQKLNDKLMGSEKFDSTSVVIEPVLEGSEPRQE
DDEKQLTIAMSMKTLQGAFYVLAIGSILSGFLLLIEMRSHDKFEKNKRTKRVEAPFVHIRK
VPNKFQNRLYDLKE*
>MsanIRDelta1a
LTIDNDYGWSLGNGSFGGVTGLLQKEEIDFSATGVFIRPDRMSAVDFTVGTVALRTTAIFR
QPSLSSVHNILLLPFTFDVWLGICATLCAFVLTLIFLMRVNNYFVEKKYDTLSIPDIVTLVHG
AICQQGSYNSTSTLGSIRVVVTILFFTSLFIYTSYSASIAALVQSNSNSIKSIKNLAESSMTFS
AQISQYGKYYFEETEDSELRKLYMTKMVPSGNKSFVKAAEGIERIRTEFHGFQVEVMSAY
KIISKQWREEEKCGLGEIQLFKIPLLSIALVKKSGHKDIFKQKLIQQMEVGLNKRIASQWIPP
KPSCGSSSRAKQYISVSVKETYLTLAIFGFGVCISLLIFILEVLQHAWMNRGSKKN*
>MsanIRDelta1b
MQNDWILIDSRRNDSYVLNTSAIALETFEMHLSEAYILPDSNVFLFIDTASGWEIWEGFKV
GKMERIRVNRHGIVTRDEIYFDKNDSISFKSNLRGITLKATTVISEPSKFKGFYPFADTDLDT
FAQMHYDLNLILQDQLNFKIDLGIVNSSGWDMGNASFSGLTSQLQREECDFSGIGAFIRND
RMTVIDYTVGTFYRQAAALFKQPPLSSVHNICILPFNFEVWMVTLFTFIGFTILIAFLSRMT
RRFKENEEEALNVLDSVTIVHGAICQQGYTMNLNAGSIRVAIFVLFLTAVFLFTSYSASIVAL
LQSPSNSIKTINDLVESSMTFSAQDNPYNDVYFGETDDPLLRKLYDKKMKPYGSQKFTLAS
TGIARIRTEFHAFMIDFVSAYKLISQLWREEEKCGISEIQLFKLPMLALAVVKRSGYKDILK
QKLIHQQEVGLKNRIIRRWIPAKPICDSSNRANQFVSVSIKEIYPMLQIYGFGLCISILILFFEI
AYNTYTCRSENLHSNTYFSILRFMYNKR*

File S5. The amino acid sequences of 19 sensory neuron membrane proteins (SNMPs) from 10 Hemiptera species.
>MperSNMP1.1
MGAPTTLTVIGIIFLLFGVFVGWFAFPKMIHKKILESKALNPRSNMRQMWSHPPIYADFKIY
LFNVTNPEEAQKGEKVIIKEVGPYVYQEWKEKENLIDDIDADTVEFSFKNTFVLDEMSTLP
LTGDEIIVMPHLAMIGMVTMTKIMKPAALGLVNKAIPYLYPDQTSAFMMGTANDIMWNG
LDINCTSGEFAAVAICTQIRQNSASLHKISKDHFKFSLFGVKNGTIESNRYTVKRGYTTPATE
VGQVIRFNDKHKQEVWPGEECNKIYGTDTTIFQPFITKDTNLASFSGDVCRSLAPDYVQET
KYNGLNVFEYSAILVKPEEKCFCLNQKKCLKPGALDLTNCSGAPIIATLPHFYKSEEYLNN
VDGLHPDAEKHRIQMYFEPMTGSPLLGYKRLQFNIFLKKESKISVMKTLNEDEKLIPLFW
VEEGIALNKTWTNQIKNKLFLPITIMKYVKYIFVAFGIVFIILAVIVNYNSVKTMEVTPKY
>MperSNMP1.X2
MRQMWSHPPIYADFKIYLFNVTNPEEAQKGEKVIIKEVGPYVYQEWKEKENLIDDIDADT
VEFSFKNTFVLDEMSTLPLTGDEIIVMPHLAMIGMVTMTKIMKPAALGLVNKAIPYLYPDQ
TSAFMMGTANDIMWNGLDINCTSGEFAAVAICTQIRQNSASLHKISKDHFKFSLFGVKNGT
IESNRYTVKRGYTTPATEVGQVIRFNDKHKQEVWPGEECNKIYGTDTTIFQPFITKDTNLA
SFSGDVCRSLAPDYVQETKYNGLNVFEYSAILVKPEEKCFCLNQKKCLKPGALDLTNCSG
APIIATLPHFYKSEEYLNNVDGLHPDAEKHRIQMYFEPMTGSPLLGYKRLQFNIFLKKESKI
SVMKTLNEDEKLIPLFWVEEGIALNKTWTNQIKNKLFLPITIMKYVKYIFVAFGIVFIILAVI
VNYNSVKTMEVTPKY
>SflaSNMP1-like.X1
MGAPTTLTVTGIVFLSMGVFIGWFAFPRMIHNKILESKAINPRSPMRQMWEHPPITADFKIF
LFNVTNPEEAQKGEKVILKEVGPYFYHEWKFKEDLEDDSKSDTVEFSFKNTFIFDEMMTL
PLTGEEIITMPHLAMLGMVTMTKMTKPAAIGLINKAIPLLYPDQTNAFMTATANDIMWNG
LDINCTSSEFPAVAVCTQIRQNSASLHKINNNHFKFSLLGVKNGTVESNRYVVNRGLKMPM
DVGQVIRFNGKHKMDVWPGDECNRIYGTDTTIFQPFISTDTNLASFSGDLCRSLNTDYVQ
ETQYNGLKVFEYTAMLVKPEEKCFCLNKKKCLKHGALDLTNCSGSPIIATLPHFYKSEEYL
KDVDGLSPKDENHRIKMYFEPMTGTPLLGYRRMQFNMFLKKEPKITVMKTLNEEEKLVP
LFWVEEGIALNKTWTSQIKNKLYLPITIVKYAKFVLVALGIVFIILAVVLNYNSVKTMEVTP
NI
>SflaSNMP1-like.X2
MNVPCSVCVVVVNPFVVLSKAINPRSPMRQMWEHPPITADFKIFLFNVTNPEEAQKGEKVI
LKEVGPYFYHEWKFKEDLEDDSKSDTVEFSFKNTFIFDEMMTLPLTGEEIITMPHLAMLG
MVTMTKMTKPAAIGLINKAIPLLYPDQTNAFMTATANDIMWNGLDINCTSSEFPAVAVCTQ
IRQNSASLHKINNNHFKFSLLGVKNGTVESNRYVVNRGLKMPMDVGQVIRFNGKHKMD
VWPGDECNRIYGTDTTIFQPFISTDTNLASFSGDLCRSLNTDYVQETQYNGLKVFEYTAML
VKPEEKCFCLNKKKCLKHGALDLTNCSGSPIIATLPHFYKSEEYLKDVDGLSPKDENHRIK
MYFEPMTGTPLLGYRRMQFNMFLKKEPKITVMKTLNEEEKLVPLFWVEEGIALNKTWTS
QIKNKLYLPITIVKYAKFVLVALGIVFIILAVVLNYNSVKTMEVTPNI
>SflaSNMP1-like.X3
MRQMWEHPPITADFKIFLFNVTNPEEAQKGEKVILKEVGPYFYHEWKFKEDLEDDSKSDT
VEFSFKNTFIFDEMMTLPLTGEEIITMPHLAMLGMVTMTKMTKPAAIGLINKAIPLLYPDQT
NAFMTATANDIMWNGLDINCTSSEFPAVAVCTQIRQNSASLHKINNNHFKFSLLGVKNGTV
ESNRYVVNRGLKMPMDVGQVIRFNGKHKMDVWPGDECNRIYGTDTTIFQPFISTDTNLA

SFSGDLCRSLNTDYVQETQYNGLKVFEYTAMLVKPEEKCFCLNKKKCLKHGALDLTNCS
GSPIIATLPHFYKSEEYLKDVDGLSPKDENHRIKMYFEPMTGTPLLGYRRMQFNMFLKKE
PKITVMKTLNEEEKLVPLFWVEEGIALNKTWTSQIKNKLYLPITIVKYAKFVLVALGIVFIIL
AVVLNYNSVKTMEVTPNI
>ApisSNMP1
MGAPTTLTVIGIIFLLFGVFVGWFAFPKMLNNKILESKSLNPRSNMRQMWSHPPLSADFKI
YLFNVTNPIEAQKGEKVIIKEIGPYVVYHEWKEKENLIDDIDADTVEFSFKNTFVFDEMSTLP
LTGDEIIVMPHLAMIGMVTMTKMMKPAALGLVNKAIPYLYPDQTSAFMMGTANDIMWN
GLDINCTSEEFASVAICSQIRQNSESLHKISKDHFKFSLFGVKNGTIESNRYTVKRGYTSPAT
EVGQVIRFNDKHKMDVWPGDECNKIYGTDTTIFQPFITKDTNLASFSGDICRSLTPDYVQE
TKYNGLNVFEYSAMLVKPEEKCYCLNKKKCLKPGALDLTNCSGAPIIATLPHFYKSEDYL
NNVDGLSPQVEKHKIQMYFEPMTGTPLLGYKRLQFNIFLKRESKINVMKTLNEDEKLIPLF
WVEEGIALNKTWTNQIKNKLYLPITIMKYVKYIFVVFGIVFIILAVVVNYNSVKTMEITPKY
>DnoxSNMP1
MGAPTTLSVIGIIFLLFGVFVGWFAFPKMIHKKILESKSINPRSAMRQMWSHPPIYADFKIY
LFNVTNPEEAQKGEKIIIKEVGPYVVYHEWKEKENLIDDMDADTVEFSFKNTFIFDEMSTLP
LTGDEIIVMPHLAMIGMVTMTKMMKPAALGLVNKAIPFLYPDQTSVFMMGTANDIMWNG
LDINCTSREFAAVAICTQIRQNSASLHKISNEHFKFSLFGVKNGTIESNRYTVKRAYTSPATE
VGQVIRFNDKHKMDVWPGDECNKIYGTDTTIFQPFITKDTNLASFSGDICRSLVPDYVRET
KYNGLNVFEYSAILVKPEEKCFCLNQKKCLKPGALDLTNCSGSPIIATLPHFYKSEDYLNN
VDGLSPNVEKHKIQMYFEPMTGSPLLGYKRLQFNMFLKKESKINVMKTLNEDEKLIPLFW
VEEGIALNKTWTNQIKNKLYLPITIIKYVKYIFVIFGIVFIILAVVINYNSVKTMEVTPKY
>AcraSNMP1
MGSKSLNPRSTMRQMWSHPPISADFKIYLFNVTNPEEAQKGEKVILKEVGPYVVYHEWKE
KENLIDDINEDTVEFSFKNTFVFDEMSTLPLTGDEILVMPHLAMIGMVTMTKMIKPAAIGL
VNKAIPYLYPDQTSAFMMGTANDIMWNGLDINCTSGEFAAVAICSQIRQNSASLHKISKDH
FKFSLFGVKNGTIESNRYTVKRGYKMSPLEVGQVVRFNDKHKMDVWPGEECNRIYGTDT
TIFQPFITPDTNLASFSGDICRSLSPDYLHETKYNGLNVFEYSAVLVKPEEKCFCLNQKKCL
KQGALDLTNCSGAPIIATLPHFYKSEEYLNNVDGLSPEVEKHRIQMYFEPMTGTPLLGYKR
LQFNIFLKKESKISVMKTLNEDEKLIPLFWVEEGVALNKTWTNQIKNKLYLPITIMKYVKYI
FVALGIVFIILAVVVNYNSVKTTEIAPKY
>AgosSNMP1
MGAPTPLTVIGIIFLLFGVFVGWFAFPKMLHKKILESKSLNPRSTMRQMWSHPPISADFKIY
LFNVTNPEEAQKGEKVILKEVGPYVVYHEWKEKENLIDDINEDTVEFSFKNTFVLDEMSTL
PLTGDEILIMPHLAMIGMVTMTKMIKPAAIGLVNKAIPYLYPDQTSAFMMGTANDIMWNG
LDINCTSGEFAAVAICSQIRQNSASLHKISKDHFKFSLFGVKNGTIESNRYTVKRGYKMSPL
EVGQVVRFNDKHKMDVWPGEECNRIYGTDTTIFQPFITPDTNLASFSGDICRSLTPDYLHE
TKYNGLNVFEYSAVLVKPEEKCFCLNQKKCLKQGALDLTNCSGAPIIATLPHFYKSEEYLN
NVDGLSPEVEKHRIQMYFEPMTGTPLLGYKRLQFNIFLKKEPKINVMKTLNEDEKLIPLF
WVEEGVALNKTWTNQIKNKLYLPITIMKYVKYIFVAFGIVFIILAVVVNYNSVKTTEITPKY
>RmaiSNMP1-like
MGAPTTLTVIGIIFLLFGVFVGWFAFPKMLHKKILESKSLNPRSTMRQMWSHPPISADFKIY
LFNVTNPEEAQKGEKIIIKEVGPYVVYHEWKEKENLIDDIDEDTVEFSFKNTFVFDEMSTLP
LTGDEILVMPHLAMIGMVTMTKMMKPAALGLVNKAIPYLYPDQTSAFMTATANDIMWNG

LDINCTSGEFAAVAICSQIRQNSASLHKISKDHFKFSLFGVKNGTIESNRYTVKRGYKMSPL
DVGQVVRFNDKHKMEVWPGDECNRIYGTDTTIFQPFITRDTNLASFSGDICRSLAPDFLQE
TKYNGLNVFEYSAVLVKPEEKCFCLNQKKCLKHGALDLTNCSGAPIIATLPHFYKSEEYLN
NIDGLNPEVEKHRIQMYFEPMTGTPLLGYKRLQFNIFLKKESKISVMKTLNEDEKLIPLFW
VEEGVALNKTWTNQIKNKLYLPITIMKYVKYIFVVFGVVFIILAVVVNYNSVKTMEVTPK
Y
>AlinSNMP1a
MGAPLRLGVAGGALFLFGSVFGFWGFHKFLNSQIAQTVQLKKGNEMRDTWATFPVALEF
KVYLFNLTNPEEVQNGGKPKVQEVGPYFFDEWKSKGNFEDDSAEDTVSFNMKAVWYFQ
KDRSEGLTGDEMITIPHPVVFSMIAQVERDKPGALPMLAKALPALFNNLTSPFIAARAMDI
LFDGLPINCSSKEFGPKAVCTLINANPKGLIKKSPELFLFSFFGPKNGTLDEGRFTVKRGIND
PKEVGLMVKYNNKTKLDVWAGPECNTLSGTDSTIFPPFIDDSEDIVSFSPDLCRSLGAKFR
YKITYKGVPGNHYTADLGDMSANEDEKCYCPTPTTCLKKGAMDITKCAGAPIILTLPHYY
LADPSYLDEVEGLHPEEEKHQIFLNFEPITGTPLGARKRLQFNIKSHPVKKIPFMKSLPTTM
IPLMWIEEGLELDQKFIDILNANLFRVMKIVGVSKWVMMLLGLGMGGFGAFLYYKRKGE
AGQPSEKSPTPKTVQVESISGKF
>AlinSNMP1b
MPSQKSESRSNSESKMSQYPRVSQVSKPTKGSRATSPVFSNLMERMREMPTKIKEAPPRQF
GKFGAAMVAGGVGFGWVAFPYILSFAISKMVNLAPGGEIHDIWKDIPQSLDFNIWIWNVT
NPMEVQNGGKAVLQEVGPYRYIEWKKKVDLIDNPADDEITYSSLNTWYFQKDRSYPLTG
DEIVTIPHLPLMSMLLVAEQDFPPAMMTVLNAAIPRIYGKLDSVFMQIKAKDLLFDGYPID
CTSRDLIGRTVCVAVKANSKPLVKNGRNKYLFSVLGTKNATPEDVRITVKKGTVNTYDIG
KVVKVNGNPMNSVWKDECNVLDGTDATIFPPYRSADNVSIVAYATDICRSIRGTYIGEGSY
NGVRGHQYAVDLGDMSSNPKDVCYCIKKCYKKGTVDLTKCQGAPLVGTLPHFYLADESY
LDGVIGMKPDREKHQITFIMEPITGVPLLARKRFQFNVDMHPIRFVNVTKNIRPTLFPILWV
EEALDLGPELMGFLQARLLTNLTLVDIVKWTLIVVGAGIGIMGIVKHQMEKEQRKKHERG
ASVSPAPSNASQERLVGQSAFRSDSEFSFKSSEMLMDPARLTGASKTTPPPLIPHPHIPTPPQ
VFTLERSLQERLSPEVEGIPPVEVPPSRLSVVTSVTPVEESAPAAGAQPGSKPASGKSKK
>AlinSNMP2a
MMRNGWTSVDLRMGNIHINRVLYLGAFGAVIFIIGLFFATSGTDMMINSKIKKGIVLEEGSE
GLKRFQKTPFPLEFKVFLFNITNTDDVMMGGKPVLTEMGPYTYDLYKEKPELKFLKDGMI
EYNMTYQFHFNAQKSRGSESDMVTGLNVPLLGTATMVEQTFPMGLGFLNNAIPFLFPNIT
DIFVTTTVKDLLFDGILLRCNYTSGPAMPICNGLKGRAPPTIWREEETKNYRFAMFRHKNK
TSEGPYKVKTGKGDVTEVGQIVEYQHRQTLKNWDKNSSCTIIKGTDTTIFGPLKNPHDDL
YIFVPDVCLSFTANYVNTSIQNGIPLNKYFAAEKNMASYSKDPDNLCRCAKDDEGVRHCL
KDGVIDASPCQGAPVIMSNPHFLDADAEYQNAVVGLKPIEEKHKTFVMLEPKTGAPVEGR
KRMQMNLKVKKVNSITLLENVTERIIPLLWIEEGTRLEGPLLQELQKLYHVMGLLGTFSW
VLLVAGLVIMGIAGVLYLKVRHLFCFAGTQIVAPVDSSIGGAQKMNTFGVTNQGSDDYQE
HGYPGTAIYPQLGDGQGKNGDLVHTVAHPQAR
>AlinSNMP2b
MPSLNFVNELLELGFPELLRDNYAMRLNRTVDELLFSGITTHCPPNASLSAATVCSILRHFP
GLKSLQKYPNGDMNVGIMRFKNDTLSDTYEVYRGNHDFDKIGQIVTLNGQQSVDNWYG
DECNKVAGSYGETLLKPFLTEDSTMKVYGSDLCSSLPVGFKETSSYEGVDSFKFGPQKKF
LGSVVDYPENYCYCPGSIDGITLGQGCMKAGAMEFSACQAVPVVLSFPHFYKASSHFQNA

VGGLDPDSDKHESYIHLEPITGIPLKGVKRIQINFQMKGTPAMKITKNARDTLIPFLWVEEV
AALGDDQVNLLKDMLLKMLKILSIVRWVLIAVGSLMVLVGCVMSFLSARKEHRHQY
>AlucSNMP1a
MGAPLRLGVTGGVLFLFGSIFGFWGFNKFLNSQIAQTVQLKKGNEMRDNWAKFPIALEFK
IYLFNLTNPDEVQEGGKPKVQEVGPYFFYEWKSKGKLEDDPSDDTVSFNMKAVWYFQKD
KSEGLTGDEIINIPHPVVFSMIMTVERDKPGALPMLAKALPALFNNLTSPFVSARAMDILFD
GLPINCASKEFGPKAVCTLINANPKGLVKKSPELFLFSFFGPKNGTLDEGRFTVKRGINDPK
EVGMMVKFNNKTKLDVWSTDECNQLTGTDSTIFPPFIDDSEDIVSFSPDLCRSLGAKFRYK
ITYKGVPGNHYTADLGDMSANEDEKCYCPTPTTCLKKGALDITKCAGAPIVLTLPHYYLA
DPSYLDEVEGLHPEEEKHQIFLNFEPITGTPLGARKRLQFNIKSHAVKKIPFMKTLPTTMIP
VMWIEEGVELDQKFIDILNANLFRVMKIVGVSKWVMMLLGIGMGGFGAFLYYKRRGAA
GGSEKPPTPKTVQVESISSGKF
>AlucSNMP1b
MTSHKSESRMSKMSESKMSQFPRSTKLSREQQGSRAKSPVFMNLMERMREMPTKIKEAP
PKKFGKFGAAMVAGGVGFGWVAFPYILSFAISKMVNLAPGGEIHDIWKDIPQSLDFNFWV
WNVTNPMEVQRGAKPVLQEVGPYRYIEWKKKVDLVDNPADDEITYSSLNTWYFQKEKS
YPLTGDEIVTIPHLPIMSMLLVAEQDFPPAMLTLLNAAIPKIYGRMDSIFLNIKVRDLLFDGY
PIDCTSRDLIGRTVCVAVKANSKPLVKNGRNKYLFSVLGTKNGTPEDVRLTVKKGTMNTF
DIGKVVKINGSPLNTVWKDECNVLDGTDATIFPPYRSADNISIVAYATDICRSIRGSYIGEGT
YNGVKGHQYVVNLGDMSKNPKDACFCVKKCYKKGTVDLTKCQGAPLIGTLPHFYLADE
SYLDGVIGLKPEPEKHQITFIMEPITGVPLLARKRFQFNVDMHPVQFVNLTKNLRPTLFPVL
WVEEALDLGPELMGFLQARLLTNLTLVDIVKWTLIVVGCGIGIMGLIKHQMEKEQQKKHE
RGASVSPAPSNASQEQLIGQSAFRSDSEFSFKSNEMLMDPSQLTGMSKTTPPPIIPHPHMPT
PPEVFTLERSLQERLSPEVGGIPPVEIPPSRLSIVRDMSPINESSVPGVPSEPNPVSGKSRKSM
K
>AlucSNMP2a
MQRNGWATVDFKMGNISINRVMYLAGFGAIVFLIGLFFATSGTDMMINAKIKKSIVLEEDS
EGLKRFQKMPFPLEFKVFLFNITNPDDVMMGGKPILTEMGPYTYDLYKEKPDLKFVKDG
MIEYNMTYQFHFNHQKSHGSESDVVTALNVPLLGTAVMVEQTFPMGLGFLNNAIPFLWP
NVTDIFMSVTVADLLFNGVLIQCNYTSGPAMPICNGLKGRAPPTIWREEDTKHFRFAMFRH
KNKTTEGPYKVKTGKDDISEVGQIVEYKHRNTLKNWDKNSSCTVIRGTDTTIFGPPKNPH
DNLYIFVPDVCLSFGASYVNTTVQYGIPLNKYTSDEKNMASAARDPDNLCRCAKDDDGV
RQCLKDGVIDASPCQGAPVIVSNPHFLDADPEYRDGVVGLNAIEDKHKTFVLMEPRTGAP
VEGRKRMQMNLRVKKVASITLLENITERVIPLLWIEEGTKLEGPLLQELQKLYHIVGFMGT
FSWVLLAAGLVILVISGALYLKVRRLFCFSGTQLVAPVDSSGVGAQRMNTFGVTNQGADD
YQEHGYPGATIYPQLGGSQEKNGDMPR
>AlucSNMP2b
MNVGILRFRNDSLSETFEVYRGNKDFNKIGQIVSIDGKRSLNHWYGEGCNKVAGSYDESL
LQPFLTQDSISNVYGSDICTSLPMSVVGSMSYKGVDCLKFSPDKKFLGSVVDYPENFCYCP
GSIEGITLGQSCLRTGAMEFAACQAVPVVLGFPHFYRASSRYQNAVGGLSPHQDKHESFVA
LEPTTGIPIEGAKRIQINFQVKGTPAMTMTKNAPDTLMPFLWLDEQVELGDEQLSMIKDTL
LKMLKIFNIVNWVLIAVGSLMVFVGCLMSFLSARRERSHPD
>MsanSNMP1

MEVGQVVRFNDKHKMDVWPSDECNKIYGTDTTIFPPFITHDTSLASFSGDICRSLNPDFVR
ETKYNGLNVFEYSAMLVKPEEKCYCLNQKKCLKPGALDLTNCSGAPIIATLPHFYKSEDY
LNNVDGLSPDVGKHKVQMYFEPMTGTPLLGYKRMQFNIFLKKEPKINAMKTLSEDEKLI
PLFWIEEGIALNKTWTNQIKNKLFLPITIMKYLKYIFVVVGIVFIILAVVINYNSVKTVEITPK
Y*

File S6. Transmembrane domains prediction of MsanORs.
>MsanOR2 | TM
MTKTPRVAAVFTAPESSEDLTIVDNKLFKAICLHQLLDPTNGSNRYYGLVLMAFMSVSLG
MQIIQLVGLYFAVNDLQRFAFATTTVSNSLLELFKGYVLVANADRLRASLEVARYEFTTCGS
WDQRLVRQSRAVLSTVLRTFTVLSWFTCFIWALAPLFAMDEYLQVTNADGTVSRYRVTIY
NVWLPVPTTVYNTTIVWTLVYAFEVIACFVNVFIWLLFDSYVVTMCFTFNAQFRTVSTSC
MNIGHRDHCFRSPSPHASEGASDDNDILNCYDELINHIKDNQSLIKKCDDFFEIIKPAIIFQII
GGSYSVITLIFLTSLTYLMGFSIISGPVLRSFFGFLSLTFELFLYCYVFNHIETEKCKMNFGLY
SCNWTAMDLKFKKTLLHAMNMNTAHRRVMKVTPMSIINLEMFANVMNMSYSIVSVLLN
SRPQK*
IIIIIIIIIIIIIIIIIIIIIIIIIIIIIIIIIIIIIIIIIMMMMMMMMMMMMMMMMMOOOOOO
OOOMMMMMMMMMMMMMMMIIIIIIIIIIIIIIIIIIIIIIIIIIIIIIIIIIIIIIIIIIIIMMMM
MMMMMMMMMMMMMOOOOOOOOOOOOOOOOOOOOOOOOOOOOOOO
OOOOMMMMMMMMMMMMMMMMMIIIIIIIIIIIIIIIIIIIIIIIIIIIIIIIIIIIIIIIIIIIIIIII
IIIIIIIIIIIIIIIIIIIIIMMMMMMMMMMMMMMMMMOOOOOOOOOOOOOMMM
MMMMMMMMMMMMMMMIIIIIIIIIIIIIIIIIIIIIIIIIIIIIIIIIIIIIIIIIIIIIIIIIIIIIIIIIIIIIIII
IIIIIIIII
>MsanOR4 | TM
SYQAVVKINKMTSIIGKKNYQRKFYQTLMTLAFFLDTSQYRFISRFIMQFYIFDWMVLVSL
AAAFTILEENSTTTLIMELIQYIIVGIYFSLIFVVFIFKKEAIMSNYDCIQTKFIQWSNKRALH
PNAAYKENIKTFKSLSIPLAILSLSIAFGPLVSTFNDIGKLPLDNRAHFVLFWPKIVDTNKPS
MYGIIYTLQVIFTISLYISVLSLNLGFMVFLNELTTQFEMLLDGINDAFKFKMDKQFPSLFID
CIRHHQIIIKFLDDLKSYFKWMILIEIIVLQVILSILIYNLTKVNASIGYKMKVGGSLLFNILPI
CFHCHIGEVVLSLHKRLSNHIYNMTWYDMPNKNKQLIVIMFQRTQRDLTLSSALFSSEKA
SRALISKVIKQVYTILNVLLKT*
IIIIIIIIIIIIIIIIIIIIIIIIIIIIIIIIIIIIIIIIIIMMMMMMMMMMMMMMMMOOOOOOO
OOOOMMMMMMMMMMMMMMMMIIIIIIIIIIIIIIIIIIIIIIIIIIIIIIIIIIIIIMMMMM
MMMMMMMMMMOOOOOOOOOOOOOOOOOOOOOOOOOOOOMMMMM
MMMMMMMMMMMMMMMMMIIIIIIIIIIIIIIIIIIIIIIIIIIIIIIIIIIIIIIIIIIIIIMMMM
MMMMMMMMMMMMMMMOOOOOMMMMMMMMMMMMMMMMIII
IIIIIIIIIIIIIIIIIIIIIIIIIIIIIIIIIIIIIIIIIIIIIIIIIIIIIIIIIIIIIIIIIII
>MsanOR43b-1 | TM
VDATSAFSKLETEKLSHSVCQWKRVIAHMVYNFFQKTGRSDVRRYATTCVEFFSYFELVVI
LIFVIFAFLSVVYSNEDLYNRIYSFSWLCFEANVFAVILIRLYYQSKFRDMYDRSQLIGGTLG
NYRRTLVILVVYYFVTSTALVIVPLLYVISFDSAEMGDPFSFPVADALPKKTGNVTVYVCK
YIVYSFILYAAHLENGFVNGTFIYFTGVVKKYFQILDVEVQEAMVNKDEQKLKIAIKRHQE
LLKLFIDMKTVYDKSILIIIEFCGLFNGFNGYIMIQCIQGVIHPKILGFSIACILASLLTMSIYCI

CGSNIYYLHDGLLKSLFEHNSCYSKNNSFKKLVIMMMTRATVPLEIKAGSIFAINLNLLVKI
LKFTYTVFNLLLTSVNRQIKENSAI*
IIIIIIIIIIIIIIIIIIIIIIIIIIIIIIIIIIIIIIIIIIIIIIIIIIIIIIMMMMMMMMMMMMMMMMMMOOOOOO
OOOOMMMMMMMMMMMMMMMMMMIIIIIIIIIIIIIIIIIIIIIIIIIIIIIIIIIIIMMMMMMMMMM
MMMMMMMMMMOOOOOOOOOOOOOOOOOOOOOOOOOOOOOOOOOMMMMMMM
MMMMMMMMMMMMMMMIIIIIIIIIIIIIIIIIIIIIIIIIIIIIIIIIIIIIIIIIIIIIIIIIIIIIIMMMMMMMMM
MMMMMMMMMMMOOOOOOOOOOMMMMMMMMMMMMMMMMMMMMIIIIIIIIIIIIII
IIIIIIIIIIIIIIIIIIIIIIIIIIIIIIIIIIIIIIIIIIIIIIIIIIIIIIIIIIIIIIIIIIIIIIIIIIIII
>MsanOR43b-2 | TM
MADIVDTFFQYTGWSDDHSFVATYSAILTYSDLAVFLFFLIFTFWSIVYWTEDLSIRIYCFL
WLFIVVHMFVSIYSRLYYQSIFRDMYRHSLIVGLPENYRRKILIVIIYYFIVSNVYVYIPMVY
TIAFDSVQMGDPFTYPFMDVFAVKKPTITVYLCQYIIYAIPVYLTHLEGCLLNTTFMHSTGV
MKRLFQTLDKQVEEAMIIRDEQKLKIAIKQHQELLKFFRKMETVYEKQILLSIEFCGIYIGL
TCFIMIQVIQGVIHPLLLGLCIGSIFEGLASITVYCIFGSIMHDLHDGTLNSLFNQQSCYFRD
KSFKQLVVIMMARVSIPLEFKAGSIFTINLNLFVKILKFTYSVFNLLLTSVN*
IIIIIIIIIIIIIIIIIIIIIIIIIIMMMMMMMMMMMMMMMMMMMMOOOOOOOOOOMMMMMM
MMMMMMMMMMMMMMMMIIIIIIIIIIIIIIIIIIIIIIIIMMMMMMMMMMMMMMMMMMM
MMOOOOOOOOOOOOOOOOOOOOOOOOOOOOOOMMMMMMMMMMMMMMMMMM
MMMMIIIIIIIIIIIIIIIIIIIIIIIIIIIIIIIIIIIIIIIIIIIIIIIIIIIIIIIMMMMMMMMMMMMMMMMMMM
MOOOOOOOOOOMMMMMMMMMMMMMMMMMMMMIIIIIIIIIIIIIIIIIIIIIIIIIIIIIIIIIIIIIIIII
IIIIIIIIIIIIIIIIIIIIIIIIIIIIIIIIIIIIIIIII
>MsanOR46a-1 | TM
MMDIRDDQNNLFNITLAKYMGIYQMLDPQTTRFRGLNVYHIVMIFIILFLCVFAVIINISVV
YYWTDNMLLSIDFIWKGMIMLYACCPIWVIVNYSNDIWDCLSITCYGFTSYSLRYRHILDR
CKERSVFITTAITFLYFTSFVIYIVSSLTLLNDIIPVKNRDGSISNYQHNIINLYLLVSGDTYNA
HYNIFYMLEVLILVFVIIPYCIFDFVTVTLCLAIRCQWQMICTAFESIGHTSLGDNLSLVDCG
EEKKKLPNKHDLIYDELKTIIMNHQAVKKKYDKFLTIFQQALLLQIVVCLTTFIILWICFILV
KINMMFNQKKFFSA*
IIIIIIIIIIIIIIIIIIIIIIIIIIIIIIIIIIIIIIIIIMMMMMMMMMMMMMMMMMMMMMOOOOOOOOOO
OMMMMMMMMMMMMMMMMMMMMMMIIIIIIIIIIIIIIIIIIIIIIIIIIIIIIIIIIIIIIMMMMMMM
MMMMMMMMMMMOOOOOOOOOOOOOOOOOOOOOOOOOOOOOOOOOOOOOOOOO
OOOOOMMMMMMMMMMMMMMMMMMMMIIIIIIIIIIIIIIIIIIIIIIIIIIIIIIIIIIIIIIIIIIIIIIIIIIIIII
IIIIIIIIIIIIIIIIIIIIIIIMMMMMMMMMMMMMMMOOOOOOOOOOOOOOOOOOOOOO
>MsanOR46a-2 | TM
YMGIDFLWKSENTLYVIYKAYFIVRYSKDIWKCLSITRYDFTSFKYQNRHILDYWRKRLTR
LTTIYAIMHLTTVSSFLLISLAFTKYKLPVKNHDGSIGYYRHNVMNLYIIVSDETYNAHYY
MFYIIETLFVSLVTSIFFTFCLLLITLCFSICCQMQVICSAFESVGHKSLRDLDSPIGDDYTAE
NIKIPSNEHDLNYNELKTIINDHQAVMEKYEMFLTLFRRVILLHIFVSSLLAITLLLTIIMSFS
NDDRYKTSEVVIGKLLCCIPSIFFDIFVVSYLFGNIHDHNDSMVFALYGSNWTEMDMKCK
KLVLLTMKLNNA
OOMMMMMMMMMMMMMMMMMMMMMMIIIIIIIIIIIIIIIIIIIIIIIIIIIIIIIIIIIIIIIIIIMMMMM
MMMMMMMMMMMOOOOOOOOOOOOOOOOOOOOOOOOOOOOOOOOOOOOOOOOO
OOMMMMMMMMMMMMMMMMMMMMMMMIIIIIIIIIIIIIIIIIIIIIIIIIIIIIIIIIIIIIIIIIIIIIIIIIIIII
IIIIIIIIIIIIIIIIIIIIIIIIIIIMMMMMMMMMMMMMMMMOOOOOOOOOOOOOOOOOOOMM

MMMMMMMMMMMMMMMMMMIIIIIIIIIIIIIIIIIIIIIIIIIIIIIIIIIIIIIIIIII
>MsanOR64 | TM
MDIWKNKKHLFNIKLSKIITLYQILDPETVKYHGRNIYHIVIACTMLCTSIVLMIGVLSGLN
YFKSNIPLSMNFFFLISFGAYTIYKIWFTIHHSNDIWDYLSITQYDFTSIGNRHRHILDRWRE
RLTWLTNIYAILVLMLTVYFIAMTSAFSEDKSLIKNHDGSIGYYRQNVLNLYLIVSDETYNT
HYYTFYIVEASVFVYLSIYFCLFDILLITLCYSMCCQMQIVCSAFELVGHRSLRDHHSPIDQ
SVGNISTNEHDLIHEELKTIIMDHQLVMKKYEDFVSLFRRMILLHIFVSSLLVIVLIFTITMSF
SSDERFKTSTNAVHKLFVFIPPTLFQIFMVCYLYGSMHNQKDAIIFALYSSNWTEMDMKCK
KLILLTMKLNNANQKK
IIIIIIIIIIIIIIIIIIIIIIIIIIIIIIIIIIIIIIIMMMMMMMMMMMMMMMMMMMMOOOOOOOOOOO
OMMMMMMMMMMMMMMMMMMMMMIIIIIIIIIIIIIIIIIIIIIIIIIIIIIIIIIIIIIIIIMMMMMMM
MMMMMMMMMOOOOOOOOOOOOOOOOOOOOOOOOOOOOOOOOOOOOOOOOOOO
OOMMMMMMMMMMMMMMMMMMMMMMMMMIIIIIIIIIIIIIIIIIIIIIIIIIIIIIIIIIIIIIIIIIIIIIII
IIIIIIIIIIIIIIIIIIIIIIIIIMMMMMMMMMMMMMMMMOOOOOOOOOOOOOOOOOOOMMM
MMMMMMMMMMMMMMMMMMIIIIIIIIIIIIIIIIIIIIIIIIIIIIIIIIIIIIIIIIIIII
>MsanORCO | TM
MGYKKDGLIKDLWPNIRLIQLSGLFISEYYDDYSGLAVLFRKIYSWITTIIIYSQFIFIVMFM
VTKSNDSDQLAAGVVTTLFFTHSMIKFMYFSTGTKSFYRTLSCWNNTSPHPLFAESHSRFH
AKSLSRMRQLLIIVSIVTIFTTISWTTITFFGESVWKVPNPETYNQTMYVPVPRLMLHSWYP
WDSSHGLGYIVAFVLQFYWIFITLSHSNLLELLFSSFLVHACEQLQHLKEILNPLIELSATLD
SSVHNPAEIFRANSAKNQSINGIDRDYNGSYVNEITEYGTKGENESNRKGPNSLTSNQEVL
VRSAIKYWVERHKHVVKYVSLITECYGSALLFHMLVSTVILTILAYQATKINGVNVFAFSTI
GYLMYSFAQIFMFCIHGNELIEESSSVMEAAYGCHWYDGSEEAKTFVQIVCQQCQKPLIVS
GAKFFNVSLDLFASVLGAVVTYFMVLVQLK*
IIIIIIIIIIIIIIIIIIIIIIIIIIIIIIIIIIIIIIIIIIIIIMMMMMMMMMMMMMMMMMMOOOOOOOOOMM
MMMMMMMMMMMMMMMMMMMIIIIIIIIIIIIIIIIIIIIIIIIIIIIIIIIIIIIIIIIIMMMMMMMMMM
MMMMMMMMMMMOOOOOOOOOOOOOOOOOOOOOOOOOOOOOOOOOOOOOOOOO
MMMMMMMMMMMMMMMMMMMMMIIIIIIIIIIIIIIIIIIIIIIIIIIIIIIIIIIIIIIIIIIIIIIIIIIIIIIIIIIIIIIII
IIIIIIIIIIIIIIIIIIIIIIIIIIIIIIIIIIIIIIIIIIIIIIIIIIIIIIIIIIIMMMMMMMMMMMMMMMMMMMMMM
OOOOOMMMMMMMMMMMMMMMMMMMMMMMIIIIIIIIIIIIIIIIIIIIIIIIIIIIIIIIIIIIIIIIIIIIII
IIIIIIMMMMMMMMMMMMMMMMMMMMMOO
